# Wnt-dependent ontogeny of acellular cementum-forming cementoblasts on the tooth root surface

**DOI:** 10.1038/s41467-026-72712-1

**Published:** 2026-05-13

**Authors:** Taishi Komori, Mizuki Nagata, Natnicha Praneetpong, Hanwen Fan, Yuxiao Zhou, Noriaki Ono, Wanida Ono

**Affiliations:** 1 Department of Diagnostic and Biomedical Sciences, The University of Texas Health Science Center at Houston (UTHealth Houston) School of Dentistry, Houston, TX USA; 2 Department of Orthodontics, The University of Texas Health Science Center at Houston (UTHealth Houston) School of Dentistry, Houston, TX USA; 3 Department of Periodontology, Graduate School of Medical and Dental Sciences, Institute of Science Tokyo, Tokyo, Japan; 4 Department of Mechanical Engineering, Texas A&M University, College Station, TX USA; 5 Division of Orthodontics, University of California, San Francisco (UCSF) School of Dentistry, San Francisco, CA USA

**Keywords:** Bone development, Mesoderm

## Abstract

Cementum is a specialized mineralized tissue that covers the tooth root surface and anchors the tooth to the surrounding periodontal ligament. The cervical acellular cementum (AC) is indispensable for periodontal attachment and represents a key target for periodontal regenerative therapies. However, the developmental origin and molecular identity of AC-forming cementoblasts remain poorly understood. Here, through an integrated spatial and single-cell transcriptomic analysis, we identify AC-forming cementoblasts as a distinct population of noncanonical mineralizing cells enriched for cell-matrix organization and Wnt signaling signatures, distinguishing them from cellular cementum-forming cementoblasts localized in the apical portion. Lineage tracing using a *Wnt inhibitory factor 1 (Wif1-creER)* demonstrates that AC-forming cementoblasts originate exclusively from peri-epithelial apical *Wif1*^+^ cells of the elongating tooth root via canonical Wnt signaling activation. Collectively, our findings uncover the unique ontogeny and molecular signature of acellular cementum, providing insights into the formation and maintenance of the periodontal attachment apparatus.

## Abstract (web summary)

Acellular cementum (AC) on the tooth root surface is essential for periodontal attachment. Here, the authors identify the origin and molecular identity of AC-forming cementoblasts arising from peri-epithelial apical cells via Wnt-mediated mechanisms.

## Introduction

Cementum is one of the least characterized mineralized tissues in mammals. It is uniquely localized on the tooth root surface, forming the interface between the tooth and the surrounding periodontal ligament^1–3^. Notably, no analogous mineralized structure exists at bone-bone interfaces, such as cranial sutures. Functionally, cementum is an indispensable component of the periodontal attachment apparatus and serves as a critical target for periodontal regenerative therapies^4^. Of particular importance, cementum prevents ankylosis (fusion) between the tooth and the alveolar bone, thereby maintaining the lifelong machinery that dissipates masticatory forces exerted upon the tooth and transmits them to the bone^5^.

Cementum is broadly classified into two types: acellular cementum (AC) and cellular cementum (CC)^6,7^. AC forms an extremely thin mineralized layer covering the cervical portion of the tooth root, whereas CC constitutes a thicker mineralized structure on the apical portion, containing embedded cementocytes^8^. AC plays an essential role in periodontal attachment, providing the anchoring site for principal periodontal fibers. Loss of AC is closely associated with attachment loss and progression of periodontitis^9^. However, unlike CC, AC shows limited intrinsic regenerative potential following periodontal destruction^10^. Efficient regeneration of AC thus remains a long-standing goal of periodontal therapy. A fundamental limitation to achieving this goal is the lack of understanding of the molecular identity and developmental origin of cementoblasts, particularly those responsible for forming AC.

Cementum originates from neural crest-derived ectomesenchymal cells during tooth root formation. Its formation is initiated immediately after the root dentin deposition through tightly regulated epithelial-mesenchymal interactions^11,12^. Cementoblasts arise from two major sources: the dental follicle (DF) and the apical papilla (AP). Within the DF, parathyroid hormone-related protein (PTHrP)-expressing cells adjacent to the down-growing Hertwig’s epithelial root sheath (HERS) represent a potent source of AC-forming cementoblasts^13,14^. In parallel, C-X-C motif chemokine ligand 12 (CXCL12)-expressing cells in the AP provide a crucial source of cementoblasts along the entire root surface^15^.

Cementoblasts are functionally distinct from, yet molecularly similar to, osteoblasts^16,17^. Both cell types express overlapping markers such as *Col1a1* (type 1 collagen) and *Bglap* (osteocalcin). However, only a few reliable markers differentiate cementoblasts from osteoblasts^18,19^. One of the most robust is *Pthlh* (encoding PTHrP), which is highly expressed in cementoblasts along the root surface but absent in alveolar bone osteoblasts^13,20^. Nonetheless, PTHrP^+^ cells on the root surface are heterogeneous, encompassing cementoblasts associated with both AC and CC, as well as peri-cementoblast cells within the periodontal ligament. While skeletal and stromal cell hierarchies have been well-characterized in bone marrow^21^, the hierarchical organization and lineage relationships of cementoblast-lineage populations remain largely unresolved, representing a major gap in our understanding of cementoblast biology.

In this study, we sought to elucidate the ontogeny and molecular identity of cementoblast lineage cells. First, we mapped the transcriptomic landscape of cementoblasts in murine molars through an integrated spatial and single-cell transcriptomic analysis. Our spatial analysis resolved the periodontium into discrete clusters, including alveolar bone, periodontal ligament, and cementum, allowing the identification of cementoblasts via co-expression of *Pthlh* and *Bglap*. For single-cell analysis, we utilized a *Pthrp-mCherry* reporter knock-in allele to isolate cementoblasts and their precursors. Second, we investigated the developmental origin of cementoblasts through in vivo lineage tracing. Based on our single-cell data, we identified *Wnt inhibitory factor 1* (*Wif1*) as a putative marker of cementoblast precursor cells. To trace their lineage, we generated a tamoxifen-inducible *Wif1*-*creER* knock-in allele, targeting Wif1^+^ peri-epithelial cells located at the apical front of the elongating tooth root. Furthermore, we conditionally inactivated canonical Wnt signaling in *Wif1*^+^ cells to examine its functional requirement in cementum formation.

Together, our findings define the developmental trajectory and molecular features of AC-forming cementoblasts as a distinct population of noncanonical mineralizing cells enriched for cell-matrix organization and Wnt signaling signatures, distinguishing them from CC-forming cementoblasts in the apical portion.

## Results

### PTHrP-expressing cementoblasts on acellular and cellular cementum in mouse molars

**Fig. 1:**
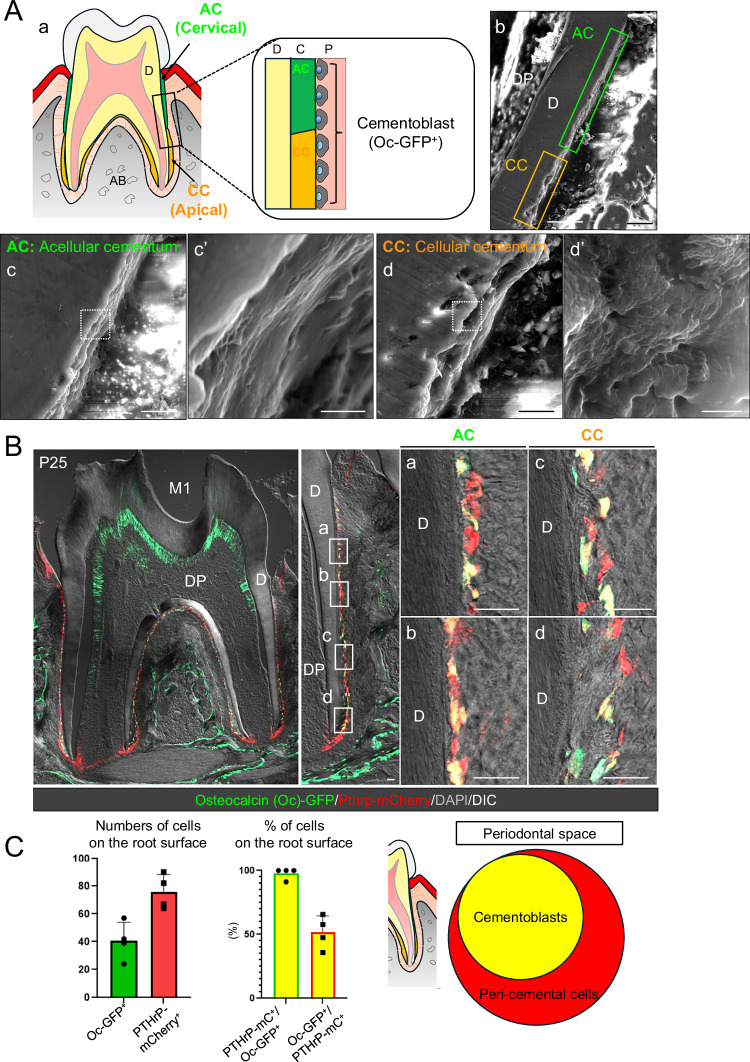
PTHrP-expressing cementoblasts on acellular and cellular cementum in mouse molars. **A** Scanning electron microscopy (SEM) images of the AC and CC. Mandibular first molar (M1) section of C57BL/6J at postnatal day (P) 25. (a) Schematic overview of the tooth root. (b) SEM image of mid-root surface showing the AC-CC transition zone. Green and orange labels/boxes indicate AC and CC regions, respectively. High magnification of AC (c, c′) and CC (d, d′). Dashed boxes indicate regions shown at higher magnification. Scale bars*:* 500 µm (b), 20 µm (c,d), and 10 µm (c’, d’). D dentin, C cementum, P periodontal ligament space, AB alveolar bone, AC acellular cementum, CC cellular cementum. **B** Pthrp-mCherry expression in mandibular first molar (M1) of *Pthrp*^*mCherry/+*^*; Oc-GFP* mice at P25. Enlarged distal-root image (middle). Higher magnification of AC (a, b) and CC (c, d). Green: Oc-GFP, red: Pthrp-mCherry, gray: DAPI/DIC. Scale bars: 25 µm. M1 mandibular first molar, DP dental pulp. **C** Quantification and summary schematic. Bar graphs show the number of Oc-GFP⁺ and Pthrp-mCherry⁺ cells per root surface (left) and the percentages of Pthrp-mCherry⁺ cells among Oc-GFP⁺ cells (right, green line), and Oc-GFP⁺ cells among Pthrp-mCherry⁺ cells (right, red line) (mean ± s.d., *n* = 4 mice per group). Dots represent individual mice. The schematic depicts the relationship between the two populations. Yellow: Oc-GFP⁺Pthrp-mCherry⁺ cementoblasts, Red: Oc-GFP^neg^Pthrp-mCherry^+^ cells. Representative images of at least three independent biological samples are shown in the figures.

We first examined the structural characteristics of the cementum using scanning electron microscopy (SEM) for anatomical reference. In murine molars, AC predominantly covers the cervical portion of the root, whereas CC is mainly distributed in the apical and furcation regions^22^. To visualize the transition from AC to CC, we analyzed the mid-root surface of the mandibular first molar (M1) at postnatal day (P) 25 when tooth root formation is complete (Fig. 1A-a). Distinct structural differences were evident along the root surface (Fig. 1A,b). The cervical cementum exhibited a laminar appearance with vertically oriented, fiber-containing terrains characteristic of AC (Fig. 1A-c-c’), whereas the apical cementum displayed a granular morphology with large hole-like indentations (Fig. 1A-d-d’). These observations indicate that the cementum undergoes a marked structural transition from cervical to apical regions, consistent with the distribution of AC and CC.

The observed structural heterogeneity suggests region-specific cementogenic activity along the root surface. To further define these regions at the cellular level, we analyzed PTHrP expression in cementoblasts using *Osteocalcin (Oc)-GFP; Pthrp*^*mCherry/+*^ double-reporter mice. Oc-GFP labels mineralized matrix-producing cells, including cementoblasts, osteoblasts, and odontoblasts, but not periodontal ligament (PDL) fibroblasts^23^. In contrast, Pthrp-mCherry specifically marks cementoblasts and their precursor cells on the root surface, but not osteoblasts or odontoblasts^13,20^. Thus, Oc-GFP^+^Pthrp-mCherry^+^ double-positive cells identify mature cementoblasts, whereas Oc-GFP^neg^PthrP-mCherry^+^ cells represent their precursors.

At the onset of cementogenesis (P6), Pthrp-mCherry^+^ cells were localized in the dental follicle (DF), negative for Oc-GFP, and distinct from cytokeratin 5 (CK5)^+^ epithelial cells of the HERS (Fig. S1A-a). By P15, when the tooth root was approximately half-formed, a subset of Pthrp-mCherry^+^ cells on the root surface began expressing Oc-GFP, while those near the apical area largely remained Oc-GFP^neg^ (Fig. S1A-b), confirming that our double-reporter system effectively distinguishes cementoblasts from their precursors.

At P25, most Pthrp-mCherry^+^ cells directly adjacent to the cementum surface were Oc-GFP^+^, while surrounding cells included both Oc-GFP⁺ and Oc-GFP^neg^ populations (Fig. 1B, a–d). Notably, Oc-GFP^neg^Pthrp-mCherry^+^ single-positive cells were particularly enriched in the apical region. Quantitative analysis revealed an average of 41 ± 14 Oc-GFP⁺ cells and 76 ± 12 Pthrp-mCherry^+^ cells per root surface, with nearly all Oc-GFP^+^ cells co-expressing Pthrp-mCherry (97.8 ± 4.2%), whereas approximately half of Pthrp-mCherry^+^ cells were Oc-GFP^+^ (51.7 ± 12.9%) (*n* = 4; mean ± s.d.) (Fig. 1C). These findings indicate that Oc-GFP^+^ cementoblasts constitute a substantial subset of the Pthrp-mCherry^+^ population on the root surface. The pattern persisted through 8 weeks of age (Fig. S1A-c).

Collectively, these results demonstrate that the Pthrp-mCherry and Oc-GFP double-reporter system reliably and efficiently identifies cementoblasts associated with both AC and CC, as well as peri-cementoblast populations across distinct stages of tooth root formation.

### Spatial transcriptomic mapping identifies two cementoblast subtypes

To comprehensively define the transcriptomic landscape of cementoblasts, we generated a cellular-resolution spatial transcriptomic map of the murine mandible at P25. We employed the 10X Genomics Visium HD platform, consisting of 2 × 2 µm barcoded grids; all downstream analyses were performed using standard 8 × 8 µm grids bins. Initial quality assessment confirmed that our dataset met high sequencing and spatial integrity standards (Fig. S2A).

**Fig. 2:**
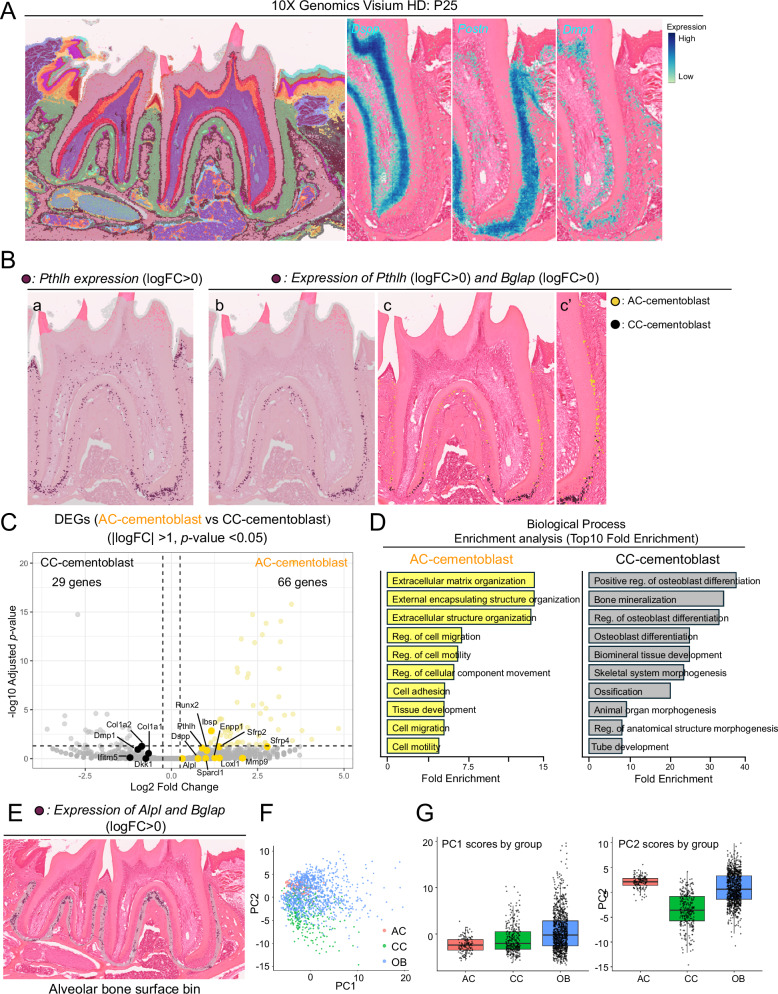
Spatial transcriptomic mapping identifies two cementoblast subtypes. **A** Tissue-level clustering and marker expression in Visium HD (8 × 8 µm bins). Left: Unsupervised clustering resolves the major cell types constituting teeth and surrounding tissues, including odontoblasts (red), dental pulp cells (purple–blue), and PDL fibroblasts (green). Right: representative marker gene expression. *Dspp* marks odontoblasts, *Postn* marks PDL fibroblasts, and *Dmp1* marks cementoblasts and osteoblasts. **B** Cementoblast subsetting and subclustering. (a) *Pthlh* expression (logFC > 0). (b) Co-expression of *Pthlh* and *Ibsp* (logFC > 0) delineates cementoblasts along the root surface. (c, c’) Subclustering of cementoblasts. Yellow: AC-cementoblasts, Black: CC-cementoblasts. (c’) shows a higher-magnification view of (c). **C** Volcano plot showing differentially expressed genes (DEGs) between two cementoblast subtypes. (|log₂FC| > 1; *p* < 0.05) Yellow and gray points indicate genes enriched in AC-cementoblasts and CC-cementoblasts, respectively. Highlighted genes are mineralization-associated genes. Statistical significance was assessed using the 10x Genomics differential expression pipeline based on a negative binomial test, and *p* values were adjusted for multiple comparisons using the Benjamini–Hochberg procedure. **D** Top 10 enriched Gene Ontology (GO) terms stratified by fold enrichment. Yellow and gray bars indicate GO terms enriched in AC-cementoblasts and CC-cementoblasts, respectively. **E** Alveolar bone osteoblast subsetting. Co-expression of *Alpl* and *Bglap* (logFC > 0) within alveolar bone surface bin. **F** PCA plot (PC1 vs PC2) of Visium HD spots classified as AC- and CC- forming cementoblasts and alveolar bone osteoblasts (OB). Red: AC, green: CC, blue: OB. **G** Boxplots of PC1 and PC2 scores for AC, CC, and OB. The center lines indicate the median. The box limits indicate the 25th and 75th percentiles. The whiskers indicate the most extreme data points within 1.5 times the interquartile range. Dots represent individual Visium HD bins. No statistical test was performed; the plots are shown for descriptive visualization of the distribution of PC scores across groups.

The spatial transcriptomic profiles successfully resolved distinct periodontal tissue compartments, including odontoblasts (red), dental pulp cells (purple-blue), and periodontal ligament fibroblasts (green) (Fig. 2A). In contrast, cementoblasts and alveolar bone osteoblasts were initially grouped into unified clusters (brown and magenta), reflecting the overall transcriptomic similarity.

To refine the identification of cementoblasts, we subset the dataset using *Pthlh* and *Bglap* expression, guided by our findings from the *Pthrp-mCherry; Oc-GFP* double-reporter model. Applying a positive expression threshold (logFC > 0) for both *Pthlh* and *Bglap* specifically delineated cementoblasts along the tooth root surface (Fig. 2B-a,b). Subsequent subclustering revealed two spatially distinct cementoblast populations: those adjacent to the cervical (AC-cementoblasts, yellow) and those localized near the apical cellular cementum (CC-cementoblasts, black) (Fig. 2B,c).

Analysis of differentially expressed genes (DEGs) identified 66 and 29 genes that were upregulated in AC-cementoblasts and CC-cementoblasts, respectively (log_2_FC > 1, *p* < 0.05) (Fig. 2C). The 66 DEGs enriched in AC-cementoblasts included *Ibsp*, a known marker for cementoblasts^23,24^, and genes related to extracellular matrix (ECM) organization and cell-matrix interactions, including *Aspn*, *Htra1*, *Thbs2-4*, and *Timp2-3*, as well as those related to fibrous matrix, including *Postn*, *Tnn*, and collagens-encoding genes. By highlighting several genes pertinent to mineralization in the plots to denote potential functional differences between AC and CC, we found that mature osteoblast-related genes including *Ifitm5* and *Dmp1* were relatively enriched in CC-cementoblasts (Fig. 2C). Gene Ontology (GO) enrichment analyses revealed that AC-cementoblasts were enriched for the terms related to cell dynamics and development, including cell migration/motility and tissue development programs, while CC-cementoblasts were enriched for those related to mineralization (Fig. 2D). This could indicate that AC-cementoblasts are primed to orchestrating periodontal attachment-interface remodeling through cell mobilization.

Historically, cementoblasts have often been compared to osteoblasts^16^. To define the cementoblast-osteoblast relationship, we further extracted alveolar bone osteoblasts (OB) by creating a band-like spatial bin along the alveolar bone surface and selecting *Alpl*^*+*^*Bglap*^*+*^ cells (*Alpl*: logFC>0 AND *Bglap*: logFC>0) (Fig. 2E). Principal component analysis (PCA) revealed that AC- and CC-cementoblasts segregated clearly along PC2, indicating their distinct transcriptional identities (Fig. 2F, G red: AC, green: CC). In contrast, OB overlapped substantially with both AC- and CC-cementoblasts (Fig. 2F, G, blue: OB), consistent with the notion that alveolar bone osteoblasts represent a heterogeneous cell population that shares a core matrix-producing program with cementoblasts. Pairwise differential gene expression analysis revealed that AC-cementoblasts were enriched for tooth- and cementum-associated genes, including *Pthlh*, the dental follicle marker *Spon1*, dentin matrix genes such as *Dsp*, and Wnt-related components, including *Wnt5a* and *Fzd1* (Fig. S3). In contrast, alveolar bone OBs were relatively enriched for canonical osteoblast markers (e.g., *Alpl*), extracellular matrix and adhesion-related genes (e.g., *Thbs1*, *Serpine2*, *Ccdc80*, *Col13a1*), as well as remodeling-associated markers (e.g., *Acp5*). Moreover, compared with CC-cementoblasts, alveolar bone OBs showed higher expression of perivascular and basement membrane-associated genes (e.g., *Rgs5*, *Col4a1*, *Olfml2a*) (Fig. S3), reflecting the dynamic bone-surface microenvironment in the alveolar bone. Together, these analyses indicate that AC- and CC-cementoblasts broadly share fundamental matrix-producing features with alveolar bone osteoblasts, while maintaining distinct cementoblast-specific features, including expression of *Pthlh* and Wnt-related components.

Collectively, our cellular-resolution spatial transcriptomic analysis identifies the unique properties and spatial arrangement of AC-forming cementoblasts in murine molars.

### Single-cell transcriptomics defines two cementoblast subtypes and their origin

**Fig. 3:**
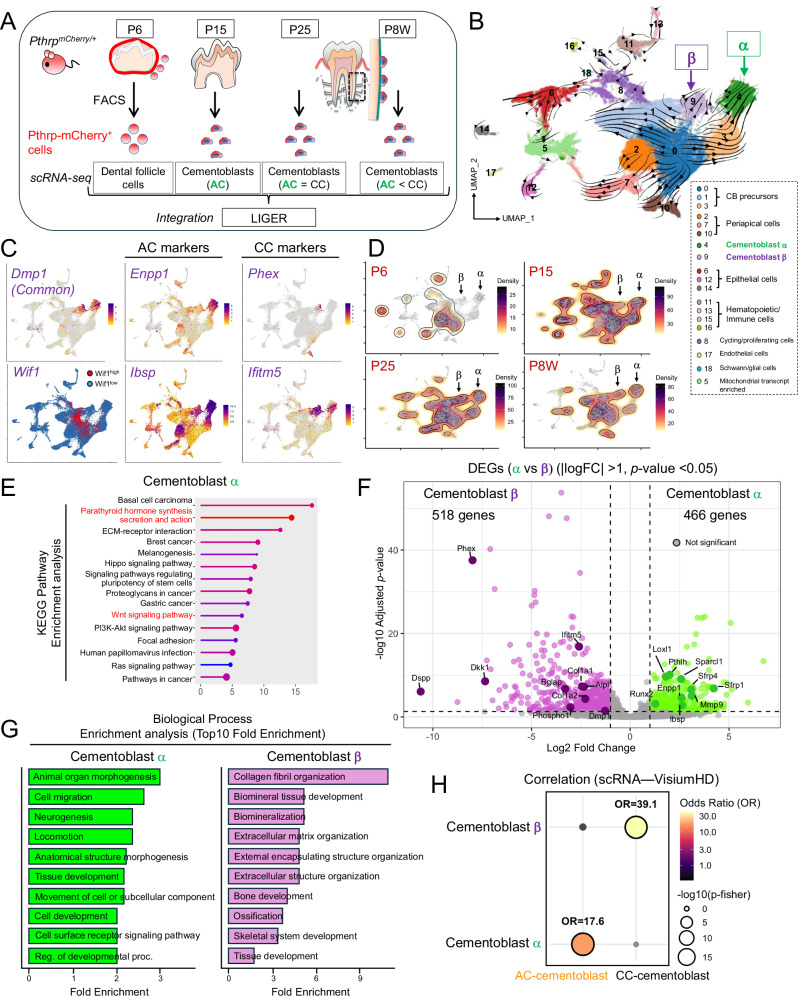
Single-cell transcriptomics defines cementoblast subtypes and their origin. **A** Experimental design. Pthrp-mCherry⁺ cells were FACS-isolated at P15 (AC formation), P25 (onset of CC formation), and 8 weeks (maturation) and integrated with our published P6 dataset (GSE120108) using LIGER. **B** UMAP plot of the integrated clusters with RNA velocity vectors (black arrows) indicating the inferred developmental trajectory from progenitors toward differentiated states. **C** Representative marker gene expression. Feature plots of *Dmp1* (pan-cementoblast), *Enpp1, Ibsp* (AC-associated cementoblast), *Phex*, and *Ifitm5* (CC-associated cementoblast). *Wif1*^*high*^ is highlighted in red (scaled expression ≥1.5). **D** Density plot showing distribution of cells at each time point. P6: enriched for precursors; P15: contributed predominantly to Cementoblastα (AC formation); P25: additionally populated *Cementoblast β* (onset of CC formation); 8 W: contributed comparably to both subtypes. **E** Top 15 enriched KEGG pathway terms of Cementoblast α DEGs. **F** Volcano plot showing DEGs between Cementoblast α and β. (|log₂FC| > 1; *p* < 0.05). Highlighted are the identical mineralization-related gene sets as in the spatial dataset. The DEGs were determined by runPairwiseDEG() in rliger. The default pseudo-bulk method was used, which aggregates cells by biological replicate and applies the DESeq2 Wald test. Adjusted *p* values are shown. **G** Top 10 enriched GO terms of Cementoblast α and *β* stratified by fold enrichment. **H** Jaccard indices comparing single-cell and Visium HD DEG sets between cementoblast subtypes. Cementoblast α and β (single-cell) correlated with AC-cementoblast and CC-cementoblast (spatial), respectively. Statistical significance of overlap was assessed using two-sided Fisher’s exact tests, with *p* values adjusted by the Benjamini-Hochberg method.

Building on the spatial transcriptomic findings, we next investigated the ontogeny and hierarchy of the cementoblast lineage using single-cell transcriptomics. To this end, we utilized the *Pthrp-mCherry* knock-in allele, which marks cementoblasts and their precursor cells along the tooth root across multiple developmental stages. Pthrp-mCherry^+^ cells were isolated by fluorescent-activated cell sorting (FACS) at P15 (completion of AC formation), P25 (onset of CC formation), and 8 weeks (maturation), followed by single-cell RNA-sequencing (scRNA-seq). We then computationally integrated these datasets with a previously published Pthrp-mCherry dataset from P6 [GSE120108]^13^ using LIGER^25,26^ (Fig. 3A). This approach enabled the reconstruction of a developmental trajectory from precursor to mature cementoblasts by capturing transcriptomic snapshots spanning sequential stages of tooth root formation. Because later-stage samples (P25 and P8W) were expected to be enriched for CC-associated cementoblasts, we anticipated that this integration would facilitate the identification of AC-specific transcriptional features.

The integrated dataset revealed substantial heterogeneity within the PTHrP^+^ cementoblast lineage. In total, 29,190 Pthrp-mCherry^+^ cells were analyzed (P6: 2101, P15: 12,926, P25: 8971, 8W: 5192 cells), which were distributed across 19 clusters. These included matrix-producing cementoblasts (Clusters 4, 9; *Dmp1/Enpp1/Ibsp and Dmp1/Ifitm5/Phex*), cementoblast precursors (Clusters 0, 1, 3; *Ogn/Cxcl12/Sfrp1, Pcp4/Vcan/Bambi/ Crabp1, and Alpl/Thbs4/Col16a1*), and periapical cells (Clusters 2, 7, 10; *Nts/Hand2/Alx1, Itgbl1/Htra1/Col18a1, and Mgp/Nrp1/Prrx1*), along with minor populations of epithelial and hematopoietic/immune cells. (Figs. 3B, S4A and Table S1). Notably, *Dmp1*^*+*^ matrix-producing cementoblasts formed two discrete clusters: one expressing known cementoblast markers (*Enpp1*, *Ibsp*) (Cluster 4) and the other expressing mature osteoblast-related markers (*Ifitm5, Phex*) (Cluster 9) (Fig. 3C, center and right panels). For clarity, we designated the *Enpp1*^+^/*Ibsp*^+^ cluster as Cementoblast α and the *Ifitm5*^+^/*Phex*^+^ cluster as Cementoblast β.

RNA velocity analysis using VeloVAE^27^ predicted a putative developmental cell origin in Cluster 0 (blue cluster) (Fig. 3B), which exhibited high expression of *Wnt inhibitory factor 1* (*Wif1*) (Fig. 3C, left bottom panel, Fig. S4B). This suggests that *Wif1⁺* cells may serve as precursor cells that give rise to both Cementoblast α and β subtypes.

To further explore developmental dynamics, we examined the distribution of cells from each time point within the integrated dataset. As expected, P6 cells were composed predominantly of precursor populations with minimal contribution to either Cementoblast α or β (Fig. 3D, upper left). By P15, cells contributed extensively to Cementoblast α, consistent with the timing of AC formation (Fig. 3D, upper right). At P25, cells also contributed substantially to Cementoblast β, reflecting the onset of CC formation (Fig. 3D, lower left). At 8 weeks, cells contributed comparably to both clusters (Fig. 3D, lower right). These temporal shifts delineate a clear developmental trajectory from PTHrP^+^ precursors to mature cementoblasts during root elongation, capturing the ontogenic transition of AC- to CC-forming populations.

We next characterized the molecular distinctions between Cementoblast α and β. KEGG pathway analysis of cluster-specific DEGs (one-vs-all) revealed both shared and unique functional signatures (Figs. 3E and S4C). Both Cementoblast α and β subtypes were enriched for *parathyroid hormone synthesis, secretion and action* (mmu04928), consistent with their shared PTHrP expression. In contrast, only Cementoblast α was enriched for *Wnt signaling pathway* (mmu04310) (Figs. 3E and S4C); for example, *Wnt5a* was elevated in Cementoblast α compared to Cementoblast β (Fig. S4D).

Pairwise DEG analysis identified 466 and 518 genes upregulated in Cementoblast α and β, respectively (log₂FC > 1, *p* < 0.05). The resulting volcano plot highlighted representative DEGs consistent with those identified in the spatial dataset (Fig. 3F). For example, *Ibsp*, *Runx2*, and *Pthlh*, relatively elevated in AC-cementoblasts in the spatial analysis (Fig. 2C), were enriched in Cementoblast α, whereas mineralization-associated genes (*Col1a1*, *Ifitm5*, *Phex*) were enriched in Cementoblast β. GO enrichment results paralleled these findings: mineralization-related terms were prominent in Cementoblast β, but not in α (Fig. 3G).

To quantitatively assess correspondence between spatial and single-cell modalities, we computed Jaccard indices of DEGs from both datasets. Cementoblast α aligned strongly with AC-cementoblasts, whereas Cementoblast β corresponded to CC-cementoblasts (Fig. 3H). Consistent with this, RNAscope analysis further revealed a higher number of *Wnt5a*^+^ cementoblasts in AC regions compared with CC (Fig. S4E).

We next sought to identify lineage-determining cues that direct *Wif1*^*+*^ cells toward AC-cementoblast fates. To gain insight into the transcriptional networks underlying the AC-forming program, we performed a branchpoint-focused analysis of the origin-state population (Cluster 0), which gives rise to both AC- and CC-cementoblasts (Fig. S5A). CellRank-based fate bias analysis revealed AC- and CC-primed subpopulations within Cluster 0 (Fig. S5B). We then conducted branchpoint differential expression (DE) analysis comparing AC- and CC-primed subpopulations. This analysis identified an AC-biased transcriptional module enriched for canonical Wnt/TCF-LEF-associated components and feedback regulators, including *Tcf7l1, Tcf7l2*, and *Notum*. In contrast, CC-biased cells preferentially engaged an Sp7-linked osteogenic transcriptional program (Fig. S5C). To further characterize these regulatory states, we integrated SCENIC regulon activity with the branchpoint analysis. This analysis revealed significant Tcf7l1(+) regulon activity in AC-primed cells, whereas Sp7(+) regulon activity was enriched in CC-primed cells (Fig. S5D). Notably, several TCF7L1 targets overlapped with AC-upregulated branchpoint DEGs, highlighting a concise set of candidate downstream effectors, including *Smoc2, Fbn1, Igf1, Abcc9, Ebf1* (Fig. S5E). For example, *Smoc2* has recently been implicated in WNT ligand trafficking in the mesenchymal stem cell niche of the incisor^28^. In contrast, CC formation appears comparatively less dependent on the TCF7L1/2-associated module and instead is biased toward an Sp7-centered osteogenic program. Together, these findings refine our proposed “binary switch” model by identifying distinct downstream transcriptional modules that execute AC versus CC lineage commitment and highlight TCF7L1 as a key transcriptional effector of β-catenin-signaling in *Wif1⁺* cells.

To examine the human relevance of our findings, we reanalyzed a publicly available single-cell RNA-seq dataset of the periodontium from human third molars [GSE161267]^29^ (Fig. S6A). Although a distinct cementoblast cluster was not clearly resolved, we identified a *PTHLH*^+^ subset within the fibroblast/stromal cluster (Fig. S6B). Notably, a small fraction of these cells exhibited partial co-expression of *ALPL*, *BGLAP*, and Wnt-associated genes (*SFRP2, SFRP4, WNT5A*) (Fig. S6C,D). These observations support the presence of PTHrP^+^ cementoblast-like populations in human molars.

Collectively, our integrative single-cell transcriptomic analysis delineates two transcriptionally and spatially distinct cementoblast subtypes. AC-associated Cementoblast α cells exhibit enhanced Wnt signaling and cell-matrix organization signatures, whereas CC-associated Cementoblast β cells display mineralization-oriented gene expression and likely include late cementoblasts and early cementocytes. Furthermore, *Wif1⁺* peri-epithelial cells emerge as putative precursors of both cementoblast subtypes, illuminating the cellular origin and molecular diversification of cementoblasts during tooth root development.

### Wif1^+^ peri-epithelial apical cells give rise to cementoblasts in tooth root formation

**Fig. 4:**
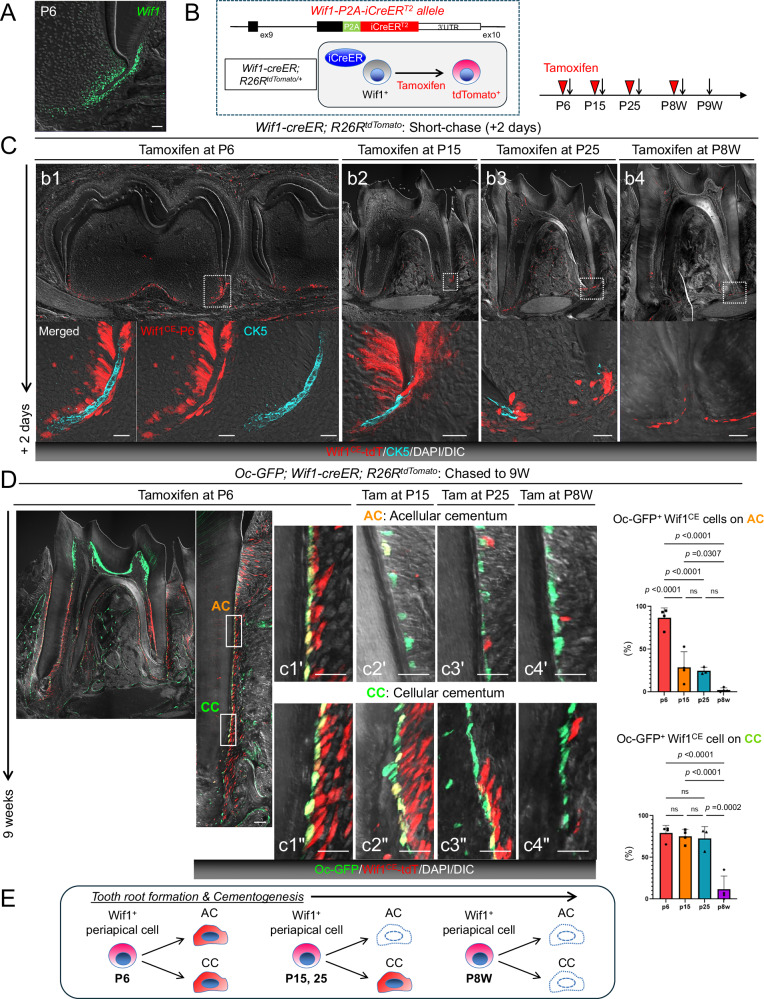
Wif1⁺ peri-epithelial apical cells give rise to cementoblasts in tooth root formation. **A** RNAscope analysis for *Wif1* mRNA expression during tooth root formation. Enlarged images of the HERS surrounding region at P6. Scale bars: 25 µm. Green: *Wif1* mRNA signal. **B** Schematic of *Wif1-iCreER* knock-in allele and in vivo lineage-tracing strategy. **C** Short-chase analysis of *Wif1-creER*^+^ cells. M1 sections from *Wif1*^*creER/+*^*; R26R*^*tdTomato*^ mandibles collected 2 days after tamoxifen injection: P8 (pulsed at P6; Wif1^CE^-P6) (b1), P17 (pulsed at P15; Wif1^CE^-P15) (b2), P27 (pulsed at P25; Wif1^CE^-P25) (b3), P8W+2days (pulsed at P8W; Wif1^CE^-8W) (b4). Dashed boxes indicate regions shown at higher magnification below. Red; Wif1^CE^-tdT, Cyan; CK5, Gray; DIC/DAPI. Scale bars:25 µm. **D** Long-chase analysis of *Wif1-creER*^+^ cells. M1 sections from *Wif1*^*creER/+*^*; R26R*^*tdTomato*^*; Oc-GFP* mandibles chased until 9 weeks of age after tamoxifen injection at P6 (c1’,c1”), P15 (c2’,c2”), P25 (c3’,c3”), and P8W (c4’,c4”). Low magnification image of M1 from Wif1^CE^-P6 (left). Boxed AC and CC regions in the left panel are shown at higher magnification in panels c1’- c4’ and c1”- c4”, respectively. Quantification of Oc-GFP^+^ Wif1^CE^-tdT^+^ on AC (top) and CC (bottom). (mean ± s.d., P6, P15 and P8W; *n* = 4 mice, P25; *n* = 3 mice). Dots represent individual mice. Statistical significance was assessed using one-way ANOVA followed by Tukey’s multiple-comparisons test. Exact adjusted *p* values are shown in the graph. Scale bars: 50 µm (low magnification image), 25 µm (high magnification image). Red: Wif1^CE^-tdT, Green: Oc-GFP. **E** Summary schematic. *Wif1-creER*⁺ peri-epithelial apical cells transiently generate AC-forming cementoblasts at the onset of cementogenesis, a capacity that declines over time, while the potential to generate CC-forming cementoblasts persists until mid-root formation. CK5 cytokeratin 5. Representative images of at least three independent biological samples are shown in the figures.

We next examined the in vivo expression pattern of *Wif1* during tooth root formation using RNAscope, which we identified as a putative marker of common cementoblast precursor cells above. *Wif1* transcripts were detected exclusively in dental follicle (DF) and dental papilla (DP) mesenchymal cells surrounding HERS in the apical region at P3 and P6 (Fig. S7A). Here, we use the term “peri-epithelial” to denote the spatially defined subset of apical mesenchymal cells that reside immediately adjacent to HERS. This peri-epithelial apical expression pattern persisted throughout subsequent stages of tooth root formation (P3-P20, Figs. 4A and S7A). Thus, *Wif1* is specifically expressed in peri-epithelial apical mesenchymal cells adjacent to HERS, suggesting that *Wif1*^+^ cells may give rise to both AC and CC-forming cementoblasts.

To test this hypothesis, we performed in vivo lineage tracing of *Wif1*^*+*^ peri-epithelial apical cells. We generated a tamoxifen-inducible *Wif1-iCreER* knock-in allele targeted to the 3’UTR using CRISPR/Cas9 (Figs. 4B and S7B, hereafter referred to as *Wif1-creER*). We first characterized the allele using a short-chase protocol. In *Wif1*^*creER/+*^*; R26R*^*tdTomato*^ mice pulsed with tamoxifen at P6, *Wif1-creER*-labeled tdTomato^+^ (hereafter, Wif1^CE^-tdT^+^) cells were localized to the apical mesenchyme surrounding cytokeratin 5 (CK5)^+^ HERS, closely mirroring the *Wif1* mRNA expression pattern (Figs. 4C-b1, 4A, and S7A). No tdTomato^+^ cells were observed in the absence of tamoxifen in *Wif1*^*creER/+*^*; R26R*^*tdTomato*^ mice (Fig. S7C), confirming the specificity and minimal background activity of the *Wif1-creER* allele. Additional short-chase analyses of *Wif1*^*creER/+*^*; R26R*^*tdTomato*^ mice pulsed at P15, P25, and 8 weeks (examined at P17, P27, and 9 weeks, respectively) consistently revealed Wif1^CE^-tdT^+^ cells surrounding CK5^+^ epithelial cells in the apical region, specifically, HERS at P17, and epithelial rests of Malassez (ERM) at P27 and 9 weeks (Fig. 4C–b2–4). These results demonstrate that *Wif1-creER* specifically marks peri-epithelial apical mesenchymal cells located at the leading root elongation front.

We next determined the lineage contribution of *Wif1*^+^ peri-epithelial cells to cementoblasts using *Oc-GFP*; *Wif1*^*creER/+*^*; R26R*^*tdTomato*^ mice. Tamoxifen was administered at four developmental stages: P6 (at the onset of cementogenesis), P15 (completion of AC formation), P25 (onset of CC formation), and 8 weeks (maturation), and tissues were analyzed at 9 weeks (designated as Wif1^CE^-P6, Wif1^CE^-P15, Wif1^CE^-P25, Wif1^CE^-8W cells, respectively).

Wif1^CE^-P6 cells exhibited robust cementogenic potential, contributing to the majority of Oc-GFP^+^ cementoblasts on both AC and CC surfaces (AC: 87.7 ± 12.3%; CC: 79.2 ± 9.1%, Fig. 4D-c1’”). In contrast, the contribution of *Wif1*^+^ cells to AC cementoblasts declined sharply when pulsed at later stages (Wif1^CE^-P15: 28.9 ± 18.1%, Wif1^CE^-P25: 24.6 ± 4.3%, Wif1^CE^-8W: 2.3 ± 2.5%; Fig. 4D-c2’–4’). However, *Wif1*^+^ cells continued to contribute robustly to CC cementoblasts through P25 (Wif1^CE^-P15: 75.3 ± 8.4%, Wif1^CE^-P25: 73 ± 13.9%), before markedly decreasing by 8 weeks (Wif1^CE^-8W: 11.8 ± 15.9%; Figs. 4D-c2”-4”). Of note, Wif1^CE^-P6 cells also contributed to odontoblasts of the root dentin (Fig. 4D, left panel), indicating that *Wif1*^*+*^ peri-epithelial cells also include odontoblast precursor cells.

Collectively, these findings indicate that the ability to generate AC-forming cementoblasts is a transient property of *Wif1-creER*^+^ peri-epithelial apical mesenchymal cells at the onset of cementogenesis. This capacity diminishes progressively during the later stages of tooth root formation, whereas their potential to produce CC-forming cementoblasts persists until mid-root formation (Fig. 4E).

### Canonical Wnt signaling dependency of acellular cementum formation by Wif1^+^ cells

**Fig. 5:**
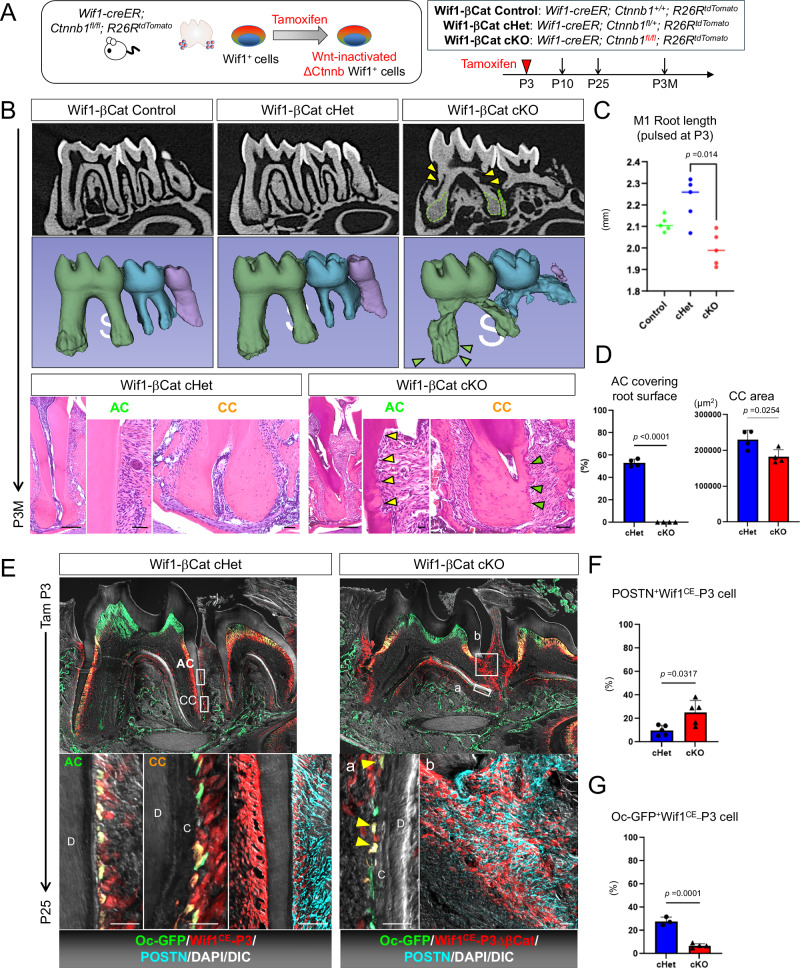
Canonical Wnt signaling dependency of acellular cementum formation by Wif1^+^ cells. **A** Schematic of experiment strategy. Conditional deletion of *Ctnnb1* (β-catenin) in Wif1⁺ peri-epithelial apical cells using *Wif1-creER*. Administration of tamoxifen at P3 (onset of root formation) with analysis at P10, 25, and 3 months of age. **B** 3D-microCT rendering and histology after chasing to 3 months of age (pulsed at P3). Defective AC formation below the cementoenamel junction (CEJ, yellow arrowheads) and CC formation near the apex (green dotted lines/arrowheads) in Wif1-βCat cKO molars. Scale bars:100 µm (low magnification), 50 µm (high magnification). **C** Quantification of M1 root length. (mean ± s.d.; *n* = 5 mice, each group). Statistical significance was assessed using one-way ANOVA followed by Tukey’s multiple-comparisons test. Exact adjusted *p* values are shown in the graph. **D** Quantification of AC coverage per M1 distal root surface (left) and CC area of M1 distal and mesial root. (mean ± s.d.; *n* = 4 mice, each group) Statistical significance was assessed using unpaired two-tailed-tests. Exact adjusted *p* values are shown in the graph. **E**–**G** Wif1^+^ cell fate analysis at P25 (pulsed at P3). Immunofluorescence staining for periostin (POSTN). Sections collected after chasing to P25 (pulsed at P3). **E** Low magnification image of M1 and M2, and enlarged images of AC (left), CC (middle), and PDL space (right) from Wif1-βCat cHet molars. Low magnification image of M1 and M2 from Wif1-βCat cKO molars and enlarged internal (left) and the external root surface (right). Quantification of percentage of POSTN^+^ Wif1^CE^-P3 cells per total Wif1^CE^-P3 cells in PDL space (**F**) (mean ± s.d.; *n* = 5 mice, each group), and percentage of Oc-GFP^+^ Wif1^CE^-P3 per total Wif1^CE^-P3 cells in AC area (**G**). (mean ± s.d.; cHet: *n* = 3 mice, cKO: *n* = 4 mice). Statistical significance was assessed using unpaired two-tailed-tests. Exact adjusted *p* values are shown in the graph. Red; Wif1^CE^-P3, Green; Oc-GFP, Cyan; POSTN. Scale bars: 25 µm. D dentin, C cementum. Representative images from at least three biological replicates are shown in the figures.

Finally, we investigated the molecular mechanism governing the differentiation of *Wif1*^+^ peri-epithelial apical cells into cementoblasts, with particular emphasis on those forming AC along the developing tooth root. Our KEGG pathway enrichment analysis revealed that the Wnt signaling pathway is specifically enriched in Cementoblast α cells associated with AC (Fig. 3E), but not in Cementoblast β cells associated with CC (Fig. S4C). Given that *Wif1* encodes a Wnt-responsive extracellular inhibitor^30^ and that canonical Wnt signaling is critical for osteogenesis, odontogenesis, and tooth root morphogenesis^31^, we examined the requirement of β-catenin in *Wif1*^+^ cells during tooth root formation (Fig. 5A).

To this end, we conditionally deleted *Ctnnb1* (encoding β-catenin) in *Wif1*^*+*^ cells at the onset of tooth root formation by tamoxifen injection at P3. We analyzed *Wif1-creER; Ctnnb1*^*+/+*^*; R26R*^*tdTomato*^ (Wif1-βCat Control), *Wif1-creER; Ctnnb1*^*fl/+*^*; R26R*^*tdTomato*^ (Wif1-βCat cHet), and *Wif1-creER; Ctnnb1*^*fl/fl*^*; R26R*^*tdTomato*^ (Wif1-βCat cKO) littermates at 3 months of age (Fig. 5A). Three-dimensional microCT analyses showed that root and crown morphologies were indistinguishable between Wif1-βCat Control and Wif1-βCat cHet molars (Figs. 5B and S8A), with no significant difference in M1 root length (Fig. 5C). In contrast, Wif1-βCat cKO molars exhibited significantly shorter M1 roots relative to Wif1-βCat cHet molars (Fig. 5C), associated with abnormally developed M2 and M3 (Fig. 5B), indicating that complete, but not partial, loss of β-catenin in *Wif1*^+^ cells impairs tooth root formation.

Histological analyses revealed a prominent structural defect in Wif1-βCat cKO tooth roots, characterized by a “notch” below the cemento-enamel junction, corresponding to defective AC and focal irregularity of the underlying dentin (Fig. 5B, right panel, yellow arrowheads). Dentin and cementum deposition resumed apically beneath the notch, accompanied by robust CC formation near the tooth root apex (Fig. 5B, right panel, green dotted lines and arrowheads). Importantly, dentin architecture appeared largely preserved in Wif1-βCat cKO molars, as indicated by the presence of organized, parallel tdTomato^+^ dentinal tubules (Fig. S9A), suggesting that the cervical cementum defect is unlikely to arise secondarily from impaired dentin formation. In addition, to determine whether the cervical root abnormalities reflected aggressive root resorption, we performed TRAP staining to assess osteoclast activities. No TRAP^+^ multinucleated cells were detected along the cervical root surface corresponding to the AC region in either genotype (Fig. S9B), indicating that the tooth root surface abnormalities observed in Wif1-βCat cKO molars represent a developmental defect in cementum formation rather than active pathological resorption. Quantification confirmed that AC formation was completely abrogated in Wif1-βCat cKO molars (Fig. 5D, % of AC coverage per root surface), whereas CC formation on the external tooth root surface was only moderately reduced (Fig. 5D, CC area). A similar phenotype was also observed in M2 (Fig. S9C). Consistent with the structural role of AC in periodontal attachment, Picrosirius Red staining revealed dense collagen bundles inserting into the AC in Wif1-βCat cHet molars, whereas fiber insertion at the root surface was markedly reduced in Wif1-βCat cKO molars lacking AC (Fig. S9D). These findings demonstrate that loss of β-catenin in *Wif1*^+^ cells selectively disrupts AC formation.

To test whether β-catenin is also required for CC formation, we inactivated *Ctnnb1* in *Wif1*^+^ cells at a later developmental stage (P25), when these cells are already restricted to CC-forming fates (Fig. 4D). *Wif1-creER; Ctnnb1*^*fl/+*^*; R26R*^*tdTomato*^ (Wif1-βCat cHet-P25/Wif1^CE^-βCat^Het^-P25 cells) and *Wif1-creER; Ctnnb1*^*fl/fl*^*; R26R*^*tdTomato*^ (Wif1-βCat cKO-P25/Wif1^CE^-∆βCat-P25 cells) littermate mice pulsed at P25 were analyzed at 3 months. The Wif1-βCat cKO-P25 molars showed normal root and CC morphology (Fig. S8B, C), and Wif1^CE^-∆βCat-P25 cells differentiated normally differentiating into cementocytes within CC (Fig. S8D), indicating that canonical Wnt signaling is dispensable for *Wif1*^+^ cells to differentiate into CC-forming cementoblasts.

We next examined how β-catenin loss alters *Wif1*^+^ cell fate. To this end, we analyzed *Oc-GFP; Wif1-creER; Ctnnb1*^*fl/+*^*; R26R*^*tdTomato*^ (Wif1^CE^-bCat^Het^-P3 cells) and *Oc-GFP; Wif1-creER; Ctnnb1*^*fl/fl*^*; R26R*^*tdTomato*^ (Wif1^CE^-∆bCat-P3 cells) littermates (pulsed at P3) after the chase. We first assessed proliferation by EdU assays. Comparable numbers of EdU^+^ cells were observed between Wif1^CE^-βCat^Het^-P3 and Wif1^CE^-∆βCat-P3 cells after 7 days of chase at P10 (Fig. S8E), indicating β-catenin deletion does not affect the proliferation of apical *Wif1*^+^ cells. When chased to P25, however, striking lineage divergence was observed: in Wif1-βCat cKO molars, many Wif1^CE^-∆βCat-P3 cells aberrantly adopted periostin (POSTN)^+^ periodontal ligament-like fates on the external root surface, coinciding with the near-complete absence of cementum (Fig. 5E, right panel). In contrast, control Wif1^CE^-βCat^Het^-P3 cells properly differentiated into Oc-GFP^+^ cementoblasts and adjacent POSTN^+^ PDL fibroblasts (Fig. 5E, left panel). Quantitative analyses confirmed a significant reduction in Oc-GFP^+^ cementoblast differentiation and a corresponding increase in POSTN^+^ cell differentiation among β-catenin-deficient *Wif1*^+^ cells (Fig. 5F, G). Interestingly, β-catenin-deficient *Wif1*^+^ cells on the furcation side of the root remained capable of forming Oc-GFP^+^ cementoblasts within CC, consistent with the preserved CC formation in Wif1-βCat cKO molars (Fig. 5E, yellow arrowhead).

Together, these findings demonstrate that canonical Wnt/β-catenin signaling is indispensable for the differentiation of *Wif1*^+^ peri-epithelial apical cells into AC-forming cementoblasts, but not for their later differentiation into CC-forming cementoblasts. Thus, *Wif1*^+^ precursors generate two transcriptionally and functionally distinct cementoblast subtypes through differential utilization of a fundamental osteogenic signaling pathway.

## Discussion

Acellular cementum (AC) is indispensable for periodontal attachment, providing a mineralized interface into which Sharpey fibers insert to anchor the tooth securely to the periodontal ligament. Histologically, AC, also referred to as acellular extrinsic fiber cementum, is characterized by its organized collagen fiber insertion, gradual mineralization, and avascular, acellular matrix, features that confer exceptional mechanical anchorage and long-term stability rather than metabolic or reparative functions^9,32–34^. AC-forming cementoblasts secrete extracellular matrix proteins such as bone sialoprotein (BSP, encoded by *IBSP*) and osteopontin (OPN), which facilitate cell adhesion and nucleation of hydroxyapatite to maintain the thin cementum layer essential for periodontal attachment. Despite its importance, the molecular identity, developmental origin, and differentiation requirements of AC-forming cementoblasts have remained poorly understood.

**Fig. 6:**
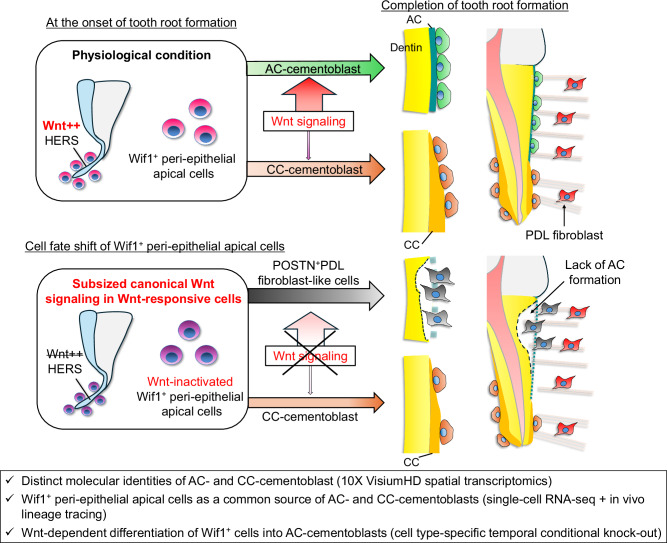
Wnt-dependent ontogeny of acellular cementum-forming cementoblasts on the tooth root surface. *Wif1⁺* peri-epithelial apical cells at the root elongation front serve as cementoblast precursor cells that generate both acellular cementum (AC)- and cellular cementum (CC)-forming cementoblasts. Under physiological conditions, *Wif1⁺* cells differentiate into AC-cementoblasts forming the thin acellular cementum that anchors periodontal fibers. As development proceeds and Wnt activity subsides, these cells adopt a Wnt-independent pathway to generate CC-cementoblasts forming thick cellular cementum with embedded cementocytes. Loss of β-catenin redirects *Wif1⁺* precursors to a POSTN*⁺* fibroblastic-like fate, abolishing AC formation.

Here, we identify AC as a product of a transcriptionally and functionally distinct subset of cementoblasts that can be generated only during the earliest stage of cementogenesis (see the concluding diagram, Fig. 6). By integrating spatial and single-cell transcriptomics, we demonstrate that AC-forming cementoblasts are noncanonical matrix-producing cells enriched in pathways related to cell-matrix organization and Wnt signaling. These cells are molecularly and spatially distinct from cellular cementum (CC)-forming cementoblasts, which predominantly express classical osteogenic programs. The distinct signatures of AC-forming cementoblasts likely reflect their specialization for forming a highly ordered, minimally cellular mineralized layer capable of anchoring periodontal fibers at the cervical root surface.

A key finding of this study is that AC-forming cementoblasts arise from *Wif1*^+^ peri-epithelial apical mesenchymal cells through a canonical Wnt/β-catenin-dependent mechanism during the initial stage of root formation. Previous studies have shown that root-forming progenitor populations, such as *Gli1*^+^ mesenchymal cells, are regulated by Wnt signaling for proper root dentin formation^35,36^. Our findings extend this concept to cementoblast lineages, demonstrating that Wnt/β-catenin signaling also acts as a lineage determinant for AC-forming cementoblasts.

In vivo lineage tracing revealed that *Wif1*^+^ cells constitute a major source of both AC- and CC-forming cementoblasts, with early *Wif1*^+^ cells generating ~88% of AC-forming and ~79% of CC-forming cementoblasts. These contributions to AC-forming cementoblasts far exceed those previously attributed to PTHrP^+^ dental follicle cells (~17%)^13^ or CXCL12^+^ apical papilla cells (~47%)^15^, suggesting that *Wif1*^+^ peri-epithelial apical cells represent a previously unrecognized precursor population that does not simply correspond to the PTHrP^+^ dental follicle or CXCL12^+^ apical papilla cell populations. Given that *Wif1* is adjacent to HERS and that HERS releases Wnt ligands^15^, our findings support a model in which *Wif1*^+^ cells are positioned to robustly respond to Wnt signals emanating from HERS to initiate cementoblast differentiation during root elongation.

Loss-of-function experiments further demonstrated that canonical Wnt signaling is essential for AC formation. Conditional deletion of β-catenin in *Wif1*^+^ cells at the onset of cementogenesis abolished AC development and redirected *Wif1*^+^ cells toward a POSTN^+^ periodontal ligament-like fate, indicating that Wnt/β-catenin signaling acts as a lineage-determining cue. Notably, CC formation was largely preserved even in the absence of β-catenin, and *Wif1*^+^ cells pulsed later in development contributed normally to CC-forming cementoblasts. These findings indicate that canonical Wnt signaling is required specifically for the differentiation of AC-forming cementoblasts, while CC-forming cementoblasts arise through a Wnt-independent pathway. Together, our data reveal a Wnt-dependent bifurcation in cementoblast fate, demonstrating that Wif1^+^ cells act as bipotent cementoblast precursor cells whose differentiation into AC- or CC-forming cells is determined by their Wnt signaling status.

These studies provide a conceptual advance in our understanding of cementum formation by establishing a cellular and molecular framework for how distinct cementoblast types are generated along the root surface. Because AC lacks intrinsic regenerative capacity following periodontal destruction, defining the developmental and signaling requirements for AC-forming cementoblasts offers new potential strategies for regenerative intervention. Targeting canonical Wnt activation in *Wif1*^+^-derived progenitors or related precursor populations may provide a rational approach for selectively promoting AC regeneration to restore periodontal attachment.

In summary, our data delineate a Wnt-dependent ontogeny of AC-forming cementoblasts from *Wif1*^+^ peri-epithelial apical precursor cells. Through spatial, single-cell, lineage-tracing, and genetic analyses, we uncover a previously unrecognized molecular and developmental dichotomy between AC- and CC-forming cementoblasts. Canonical Wnt signaling serves as a molecular switch that directs *Wif1*^*+*^ precursors toward the AC lineage, revealing how a fundamental osteogenic signaling pathway is differently deployed to generate two distinct mineralized tissues on the tooth root. These insights provide a mechanistic basis for the formation, maintenance, and potential regeneration of the periodontal attachment apparatus.

## Methods

### Mouse strains

*Pthrp-mCherry* knock-in reporter mice have been previously described (JAX032872). *Rosa26-CAG-loxP-stop-loxP-*tdTomato (Ai14: *R26R*^*tdTomato*^, JAX007914), *Osteocalcin (3.8 kb)-*GFP (JAX017469), *Ctnnb1*-floxed (JAX004152), and C57BL/6J (JAX000664) mice were acquired from the Jackson laboratory. All procedures were conducted in compliance with the Guidelines for the Care and Use of Laboratory Animals, as approved by the University of Texas Health Science Center at Houston’s Animal Welfare Committee (AWC), protocols AWC-21-0070 and AWC-24-0075. All mice were housed under specific pathogen-free conditions and analyzed in a C57BL/6 background. Mice were housed in individually ventilated cages (Tecniplast, Buguggiate, Italy). Access to water and food (irradiated LabDiet 5053, St. Louis, Missouri) was *ad libitum*. Animal rooms were climate-controlled to provide temperatures of 21 °C ± 1 °C and 30–65% humidity on a 12-h light/dark cycle (lights on at 0600 Central Standard Time). Both male and female mice were used in the study, as no sex bias was observed. For all breeding experiments, *creER* transgenes were maintained in male breeders to avoid spontaneous germline recombination. Mice were identified by micro-tattooing or ear tags. Tail biopsies of mice were lysed by a HotShot protocol (incubating the tail sample at 95 °C for 30 min in an alkaline lysis reagent, followed by neutralization) and used for PCR-based genotyping (GoTaq Green Master Mix, Promega, and PTC Tempo 48/48, Bio-Rad). Perinatal mice were also genotyped fluorescently (using a BLS miner’s lamp) whenever possible. To evaluate cell proliferation, two doses of 5-ethynyl-2’-deoxyuridine (EdU) (Invitrogen A10044) were injected shortly before analysis, at 6 and 3 h prior to sacrifice. Mice were euthanized by over-dosage of carbon dioxide or decapitation under inhalation anesthesia in a drop jar (Fluriso, Isoflurane USP, VetOne).

### Generation of *Wif1-P2A-iCreER* 3’UTR knock-in mice

*Wif1-P2A-iCreER* 3’UTR knock-in mice were generated by the University of Michigan Transgenic Animal Model Core. Briefly, CRISPR/Cas9 was used to insert *iCreER*^*T2*^ immediately upstream of the stop codon in exon 10 of the *Wif1* gene. sgRNA that targets 5’-CCTACCCCACCATCTGAAAC-3’ (PAM CGG) was designed, and on-target activity and off-target risk were evaluated with CRISPOR. A circular donor plasmid (GenScript) carrying *P2A-iCreERT2-bGH poly(A)* was flanked by homology arms adjacent to the cut site (0.33 kb 5’ arm, 1.06 kb 3’ arm). The P2A element enables bicistronic expression of Wif1 protein and iCreER^T2^ under the control of the endogenous *Wif1* regulatory elements. The synthesized sgRNA was complexed with enhanced-specificity Cas9 protein (Sigma-Aldrich, ESPCAS9PRO) to form RNPs. RNPs and the donor plasmid were microinjected into fertilized mouse zygotes and transferred to pseudopregnant B6D2F1/J females (JAX 100006). G0 founder pups were genotyped using primers (Forward: 5’-CCTAGAGGTATAGCTCACATCAGGAG-3’, Reverse: 5’-TCCTGACTTCATCAGAGGTGGCATCC-3’) to detect the correct insertion. Positive founders were crossed to wild-type mice for germline transmission of the *Wif1-P2A-iCreER*^*T2*^*-bGH polyA* knock-in allele. The newly generated *Wif1-P2A-iCreER* knock-in mice are available from the corresponding author upon reasonable request.

### Tamoxifen

Tamoxifen (T5648; Sigma-Aldrich) was dissolved completely in 100% ethanol. Subsequently, a proper volume of sunflower seed oil (Sigma S5007) was added to the tamoxifen-ethanol mixture and rigorously mixed. The tamoxifen-ethanol-oil mixture was incubated at 60 °C in a chemical hood until the ethanol had completely evaporated. The tamoxifen-oil mixture was stored at room temperature until use. Tamoxifen was administered intraperitoneally at 0.25 mg per mouse at P3, P6, and P15, and at 1.0 mg/mouse at P25 and 8 weeks.

### Scanning electron microscopy (SEM)

Mandibles were collected from C57BL/6J mice at P25. After removal of the gingiva and soft tissues, hemimandibles were rinsed in PBS and kept moist on ice. Samples were sectioned in the sagittal plane using a cryostat (CM1950, Leica, Nußloch, Germany) until the cementum layer was clearly visible. The opposite, uncut side of each sample was then embedded in polymethyl methacrylate (PMMA; Ortho-Jet, Lang Dental Manufacturing, Wheeling, US). The cementum surfaces were polished using water-based diamond suspensions with decreasing particle size to achieve a flat and smooth surface, followed by ultrasonic cleaning in deionized water to remove any residual debris. Prior to scanning electron microscopy (SEM), the polished samples were coated with a thin layer of platinum/palladium (Pt/Pd) using a sputter coater (208HRD, Cressington, Watford, UK). SEM imaging was performed on a FERA FIB-SEM system (FERA 3, Tescan, Brno, Czech) operated at 25 kV.

### Histology and immunohistochemistry, and in situ hybridization

Samples were dissected under a stereomicroscope (Nikon SMZ-800) to remove soft tissues and then fixed in 4% paraformaldehyde overnight at 4 °C. They were subsequently decalcified in 15% EDTA for 1–14 days. Decalcified samples were cryoprotected in 30% sucrose/PBS solutions and then in 30% sucrose/PBS:OCT (1:1) solutions, each for at least overnight at 4 °C. Samples were embedded in an OCT compound (Tissue-Tek, Sakura) under a stereomicroscope and transferred on a sheet of dry ice to solidify the compound. Embedded samples were cryosectioned at 14 µm using a cryostat (Leica CM1860) and adhered to positively charged glass slides (Fisherbrand ColorFrost Plus). Cryosections were stored at −20 °C in a freezer until use. Sections were postfixed in 4% paraformaldehyde for 5 min at room temperature.

For immunohistochemistry, sections were blocked with 3% BSA/TBST for 30 min, and incubated with rabbit anti-POSTN polyclonal antibody (1:500, EMD-Millipore, ABT280) and rabbit anti-cytokeratin 5 (CK5) polyclonal antibody (1:200, Abcam, ab24647) overnight at 4 °C, and subsequently with Alexa Fluor 647-conjugated donkey anti-rabbit IgG (A31573) for 3 h at room temperature. For the EdU assay, sections were incubated with Alexa Fluor 647-azide (Invitrogen, A10277) for 30 min at 43 °C using the Click-iT Imaging Kit (Invitrogen, C10337). Sections were further incubated with DAPI (4’,6-diamidino-2-phenylindole, 5 μg/ml, Invitrogen D1306) to stain nuclei.

RNAscope in situ hybridization was performed using the RNAscope Multiplex Fluorescent kit V2 (323110) with the *Wnt5a* probe (428171) according to the manufacturer’s protocol. Briefly, sections were dehydrated with 100% ethanol and treated with hydrogen peroxide (H_2_O_2_) for 10 min at RT. Samples were subsequently treated with RNAscope Protease III for 30 min at 40 °C and washed with distilled water. Sections were treated with each target probe and hybridized for 2 h at 40 °C, followed by signal hybridization by AMP and amplification by HRP-C1 or HRP-C2. The TSA Vivid fluorophore 650 (Tocris Bioscience, 7527) was added to label the HRP probe, and sections were treated with HRP blocker. Stained samples were mounted in TBS using No. 1.5 coverslips (Fisher).

### Imaging

Images for fixed sections were captured by an automated inverted fluorescence microscope with a structured illumination system (Zeiss Axio Observer 7 with ApoTome.3 system) and Zen 3.4 (blue edition) software. The Filter Set 112 SBP was used with excitation (Ex), beam-splitter (Bs), and emission (Em) filter wheel consisting of Ex. 385/30, 469/38, 555/30, 631/33, 735/40, Bs. 405 + 493 + 575 + 654 + 761, Em. 425/30, 514/30, 592/25, 681/45, 788/38 nm. The objectives used were: Fluar 2.5×/0.12, EC Plan-Neofluar 5×/0.16, Plan-Apochromat 10×/0.45, Plan-Apochromat 20×/0.80, EC Plan-Neofluar 40×/0.75. Images were typically acquired as tile scans with a motorized stage and as Z-stacks consisting of five optical sections at 0.25 μm intervals centered on the focal plane, followed by maximum intensity projection (MIP). Differential interference contrast (DIC) was used for objectives with magnifications of 10× or higher. Lookup tables were adjusted separately for each channel of the merged fluorescence images for display purposes, and these adjustments were applied uniformly across the entire field of each channel. No gamma correction nonlinear adjustment, threshold manipulation or selective enhancement/removal of signal was performed. Samples intended for direct comparison were imaged on the same day under identical acquisition settings. Representative images of at least three independent biological samples are shown in the figures. Quantitative image analysis were performed using ImageJ2 v2, GraphPad Prism v10.3.0, and QuPath v0.5.1 as appropriate.

### Cellular-resolution spatial transcriptomics

Cellular-resolution spatial transcriptomics was performed by Visium HD (10X Genomics). Mandibles were collected from C57BL/6J mice at P25. After removing the soft tissues, samples were fixed overnight with RNase-free 4% PFA (15714S; Electron Microscopy Sciences). Samples were decalcified with RNase-free 0.5 M EDTA (AM9260G, Invitrogen) for 3 days. Decalcified samples were cryoprotected in 30% sucrose/PBS solutions and then in 30% sucrose/PBS: OCT (1:1) solutions, each for at least overnight at 4 °C. Samples were embedded in an OCT compound (Tissue-Tek, Sakura) under a stereomicroscope and transferred on a sheet of dry ice to solidify the compound.

Prior to preparing analytical sections for Visium HD, a small pilot section from each sample was processed with the CELLDATA RNAstorm (Biotium) workflow to assess RNA integrity. The isolated RNA was quality-checked using Agilent RNA 6000 Pico kit (#5067-1513) by Agilent Bioanalyzer 2100 (Agilent Technologies, Santa Clara, USA). Only samples with a DV200 value of 50% or higher advanced to library preparation. Subsequently, 10-µm fixed, frozen cryosections were cut and mounted onto Schott Nexterion Slide H-3D Hydrogel Coated Slides (PN-1800434; Schott North America) to minimize the risk of tissue detachment. Sections were H&E-stained using the Visium HD workflow, and bright-field images were acquired using NanoZoomer (Hamamatsu).

The samples were destained and permeabilized according to the protocol (CG000764), and the library preparation followed the protocol (CG000685). The quality of the final libraries was examined using the Agilent High-Sensitivity DNA Kit (#5067−4626) by Agilent Bioanalyzer 2100 (Agilent Technologies, Santa Clara, USA). The qualified libraries were sequenced using the paired-end sequencing on an Illumina NovaSeq X Plus (Illumina, Inc., USA).

Data were processed with Space Ranger v3 (10× Genomics Cloud analysis) using the Mouse (mm10) reference (2020-A) and the corresponding 10× Genomics Mouse probe set. Runs were performed using the HD count pipeline; bright-field images were utilized for tissue detection and alignment. Bin-based analysis at 8 × 8 µm^2^ resolution was used for all downstream analyses. Standard run-level QC metrics were summarized (Fig. S2A). Clustering and downstream analysis were performed using Loupe Browser v8. Using its default graph-based clustering on 8-µm bins, auto-generated clusters already separated major periodontal compartments (odontoblasts, dental pulp, PDL fibroblasts). To focus on cementoblasts, bins along the root surface were feature-filtered for *Pthlh* and *Bglap* (positive expression), extracted as a subset, and re-clustered in Loupe Browser with the same settings to resolve AC-cementoblasts and CC-cementoblasts. The result of pairwise differential expressions was exported directly from Loupe Browser for downstream GO/KEGG analyses. According to the 10× Genomics differential expression pipeline, statistical significance was assessed using a negative binomial test, and *P* values were adjusted for multiple comparisons using the Benjamini-Hochberg procedure.

### Cell preparation

Cells associated with the tooth root and cementum were collected using the following protocol. Gingival tissues of detached mandibles were removed entirely using sharp forceps, and dentoalveolar components, including molars, dental pulps, dental sacs, or periodontal tissues, were carefully resected using a disposable scalpel (No.15, Graham-Field). Molars (M1 and M2) were carefully extracted from sockets in a 35 mm dish containing 3 ml Ca^2+^, Mg^2+^-free Hank’s Balanced Salt Solution (HBSS, Sigma H6648). Cells were dissociated using HBSS containing 2 Wunsch units of Liberase TM (Roche), incubated at 37 °C for 15 min on a shaking incubator (ThermomixerR, Eppendorf). Single-cell suspensions were obtained by rigorous pipetting and filtration through a 70 μm cell strainer (BD) into a 50 ml tube on ice to create a single-cell suspension. Cells were pelleted and resuspended in an appropriate medium for subsequent purposes.

### Single-cell RNA-sequencing (scRNA-seq) analysis of FACS-sorted cells

Pthrp-mCherry⁺ cells were isolated by FACS-sorting from cells pooled from *Pthrp-mCherry* mandibles. Cell sorting was performed using a four-laser BD FACS Aria III (Ex. 407/488/561/633 nm) high-speed cell sorter with a 100-μm nozzle. Pthrp-mCherry⁺ cells were directly sorted into ice-cold Dulbecco’s phosphate-buffered saline (DPBS) /1% FBS. Cell numbers were quantified by Countless II automated Cell Counter (ThermoFisher) before loading onto the Chromium Single Cell 3’ microfluidics chip (10X Genomics Inc., Pleasanton, CA). The cDNA libraries were sequenced using the Illumina NovaSeq 6000. The sequencing data were first preprocessed using the Cell Ranger v6.1 (10× Genomics). Further downstream analysis steps were performed using the rliger v2.2.1.

New datasets from P15, P25, and 8 weeks were computationally integrated with a previously published P6 Pthrp-mCherry dataset (GSE120108) using the rliger package. The downstream analysis steps include normalization, identification of highly variable genes across single cells, scaling based on the number of UMIs, dimensionality reduction (PCA and UMAP), unsupervised clustering, and the discovery of differentially expressed cell-type-specific markers. Marker genes for cluster annotation were identified using runMarkerDEG() in rliger in a one-versus-rest framework, and direct comparisons between selected clusters were performed using runPairwiseDEG(). The default pseudoBulk method was used, which aggregates cells by biological replicate and applies the DESeq2 Wald test. Differentially expressed genes were defined as those with adjusted *P* values ≤ 0.05. The scRNA-seq dataset presented herein has been deposited in the National Center for Biotechnology Information (NCBI)‘s Gene Expression Omnibus (GEO).

We utilized VeloVAE to infer the RNA velocity of Pthrp-mCherry⁺ cells. Briefly, the preprocessing steps include filtering cells and genes based on mRNA counts, constructing a KNN graph using PCA, and calculating moments based on connectivity. By employing velovae.VAE() method, initializing the rate_prior parameter with priors for transcription, splicing, and degradation rates, and setting full_vb=True, we defined a FullVB veloVAE model. The model was trained using the train() method with default parameters. The only hyperparameter used by the FullVB model is the coefficient of the KL divergence between the rate parameter distributions, which is set to 1.0 by default. The RNA velocity embedding computed by the model was then projected onto the UMAP coordinates to plot the velocity vectors that define the direction of cell differentiation in the final velocity stream plot. To compute a global map of cellular fate potential, we performed cell fate mapping inference by carrying out the protocol from scVelo. We used *scv.tl.velocity_graph() to perform basic cell state categorization, followed by* scv.tl.terminal_states() to compute the root cells and endpoints, both using the default settings. These terminal cell state probabilities were plotted using the scv.pl.scatter() function on top of the UMAP coordinates.

To quantify the correspondence between the spatial (Visium HD) and single-cell (scRNA-seq) results, we computed Jaccard indices between DEG sets across modalities (*p*-value < 0.05; log2FC > 0). For the gene universe, we enumerated the genes detected in the Visium HD and the genes detected in the scRNA-seq datasets. The background was defined as the intersection of genes detected in both modalities and was used for all enrichment tests. The significance of overlap was evaluated using Fisher’s exact test on 2×2 contingency tables, and we report odds ratios along with adjusted *p*-values.

### Three-dimensional micro-computed tomography analysis of mouse samples

Mandibles, including molars and incisors, were placed in a 19 mm diameter specimen holder and scanned using a microCT system (µCT100 Scanco Medical, Bassersdorf, Switzerland). Scan settings were as follows: voxel size, 12 µm; 70 kVp; 114 µA; 0.5 mm AL filter; and integration time, 500 ms. Three-dimensional reconstruction and analysis of the μCT images were performed with the Dragonfly 3D world workstation software (Version 2024.1; Object Research Systems Inc., Montreal, Canada) and DICOM files. For the analysis, crown length was defined as the linear distance from the cusp tip to the CEJ along the long axis of the tooth, and root length was defined as the linear distance from the cusp tip to the root apex. When the roots were curved, the measurement followed the long axis of the root in perpendicular planes to ensure anatomical alignment. Measurements were obtained from the mesial and distal roots and averaged per mouse.

### Statistical analysis and reproducibility

Results are presented as mean values ± s.d. Statistical evaluation was conducted based on the One-way ANOVA with Tukey’s multiple-comparisons test or the Mann-Whitney U test. A *p*-value of <0.05 was considered significant. Representative images shown in the figures are from experiments independently repeated at least three biologically independent samples with similar results.

### Reporting summary

Further information on research design is available in the Nature Portfolio Reporting Summary linked to this article.

## Supplementary information

Supplementary Information

Peer Review file

Reporting Summary

## Source data

Source Data

## Data Availability

The data generated in this study are provided in the Source Data file. The raw data generated during and/or analyzed during the current study are available from the corresponding author on reasonable request. The Visium HD and single-cell RNA-seq data have been deposited in the NCBI Gene Expression Omnibus (GEO) under accession numbers GSE326204 and GSE313714. The previously published P6 dataset used in this study is available in GEO under accession code GSE120108. Source data are provided with this paper.
