# Supplementary material for: Wnt-dependent ontogeny of acellular cementum-forming cementoblasts on the tooth root surface
Source: Nat Commun. 2026 May 13;17:6362. doi: 10.1038/s41467-026-72712-1 (PMC13376515; doi:10.1038/s41467-026-72712-1)
Supplement: Supplementary file 1 — Supplementary Information [file 41467_2026_72712_MOESM1_ESM.pdf]

Figure S1

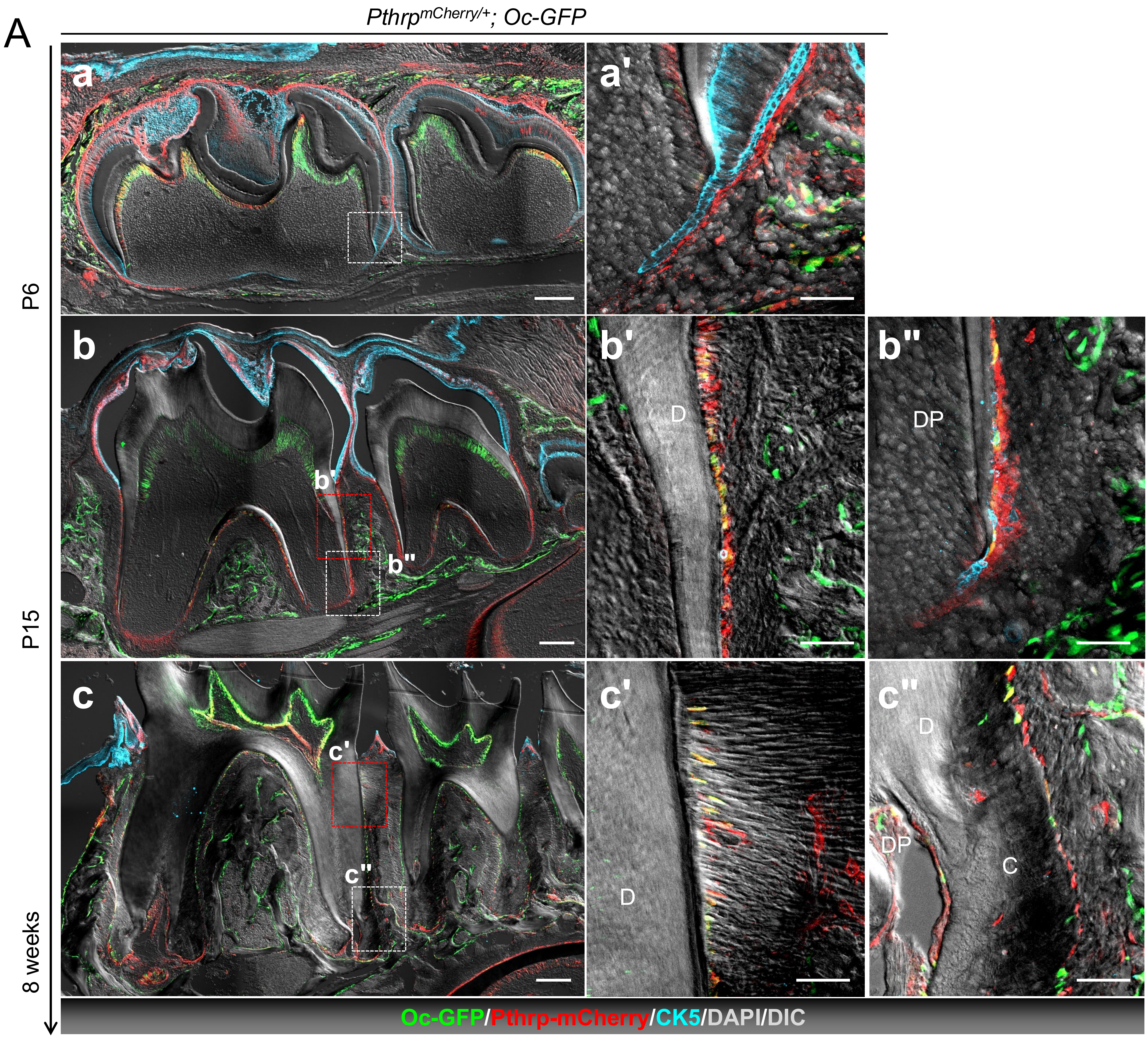

Figure S1, related to Figure 1. PTHrP-expressing cementoblasts on acellular and cellular cementum in mouse molars

Immunofluorescence staining for cytokeratin 5 (CK5) in mandibular molars from *Pthrp*$^{mCherry/+}$*; Oc-GFP* mice. Sections at P6 (a,a'), P15 (b,b',b''), P8W (c,c',c''). Left panels: overviews of mandibular first (M1) and second (M2) molars. Right panels: higher magnification of HERS (a'), AC (b',c'), and CC (b'',c''). Green; Oc-GFP, Red; PTHrP mCherry, Cyan; CK5, Gray; DIC/DAPI. Scale bars; 200 µm (low magnification), 50 µm (high magnification). DP; dental pulp, D; dentin, C; cementum. Representative images of at least three independent biological samples are shown in the figures.

Figure S2

A

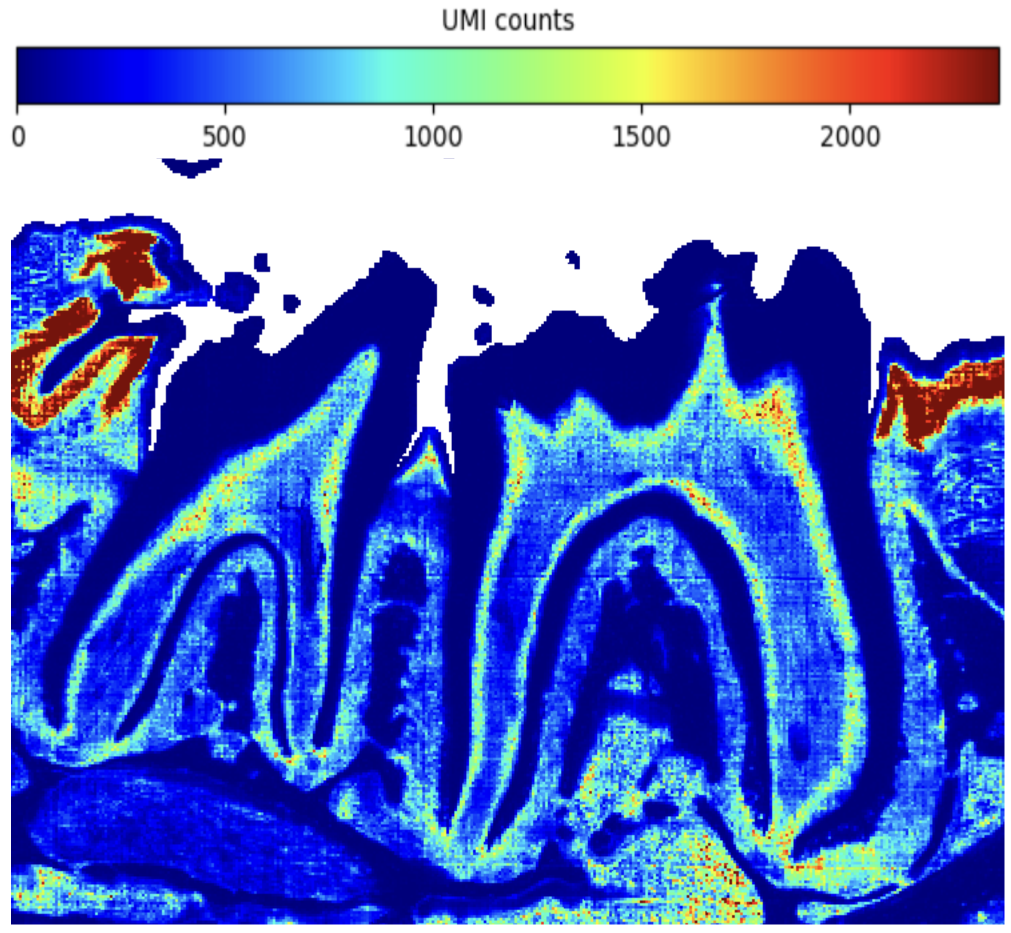

Metric	Value
Bins under tissue	216,197
Mean reads per bin	2,332.4
Mean UMIs per bin	425.9
Total genes detected	18,963
Reads to probe set	99.2%
Reads confident mapping	99.0%
Reads filtered mapping	98.6%
Total reads	504,266,809
Valid barcodes	91.2%
Valid UMIs	100.0%
Sequencing saturation	79.0%

B

AC- vs. CC-cementoblast: Differentially expressed gene (DEG) list

AC-cementoblast

AC gene	log2FC	*p*-value
Aspn	3.541	8.26e-21
Htra1	3.480	1.62e-16
Postn	2.355	1.85e-15
Thbs4	3.164	8.98e-15
Igfbp5	2.746	1.46e-14
Timp3	2.639	2.08e-13
Col3a1	2.030	5.74e-13
Ccdc3	3.153	1.14e-12
Igfbp7	2.417	1.39e-12
Gpx3	1.909	5.42e-10
Sema3c	3.127	6.30e-10
Tnn	1.923	1.51e-09
Crispld2	2.230	2.07e-09
Spon2	2.197	5.11e-09
C1qtnf3	3.115	4.76e-07
Thbs2	1.909	1.77e-06
Mafb	2.623	2.51e-06
Mmp13	2.373	3.64e-06
Fndc1	2.743	7.15e-06
Itm2a	2.579	2.40e-05
Fmod	3.520	2.76e-05
Krt5	3.275	3.68e-05
Col16a1	1.418	8.28e-05
Mgp	2.882	9.03e-05
Aebp1	1.457	9.03e-05
Col12a1	1.403	1.01e-04
Timp2	1.302	1.97e-04
Igsf10	2.023	4.15e-04
Maf	2.207	6.32e-04
Thbs3	2.009	7.07e-04

AC gene	log2FC	*p*-value
Sfrp1	1.698	8.26e-04
Lrrc17	1.686	8.58e-04
Lgals3	4.842	8.58e-04
Col4a1	3.099	0.00111
Ptprf	1.548	0.00114
Dkk3	1.662	0.00114
Selenop	1.920	0.00114
App	1.190	0.00115
Col6a3	1.672	0.00133
Cd74	3.781	0.00133
Ibsp	1.159	0.00147
Apoe	1.562	0.00205
Angptl2	1.722	0.00248
Antxr1	1.437	0.00391
S100a4	2.537	0.00470
Palld	1.695	0.00526
Ctsb	1.595	0.00550
Tpm2	1.257	0.00954
Plxdc1	4.065	0.01076
Dsp	3.505	0.01444
Tagln	1.968	0.02054
Olfml2b	1.154	0.02150
Serping1	1.920	0.02150
Afap1	2.065	0.02153
Fhl1	1.920	0.02188
Entr1	2.717	0.02213
C1ra	2.717	0.02408
Nbl1	1.137	0.02731
Myo1c	1.743	0.03339
Klf7	2.459	0.03397
Efnb2	1.882	0.03397
Pdgfrb	2.257	0.03891
Sphk1	2.579	0.03945
Itgb5	1.307	0.03945
Wls	1.550	0.04424
Id2	1.605	0.04477

CC-cementoblast

CC gene	log2FC	*p*-value
Col11a2	2.718	1.85e-15
Bambi	2.195	2.40e-05
Smpd3	1.864	2.85e-05
Aox3	3.093	4.27e-05
Mamdc2	3.100	2.82e-04
Sgms2	1.414	6.19e-04
Crtac1	3.601	0.00100
Dcn	1.212	0.00110
Pcsk6	2.065	0.00115
Pla2g5	2.490	0.00119
Col22a1	2.387	0.00168
Prss35	2.166	0.00181
Sema3d	1.980	0.00205
Cthrc1	2.328	0.00205
Car3	2.427	0.00208
Nupr1	1.209	0.00446
Slc13a5	2.016	0.00461
Tmem119	1.283	0.00798
Loxl3	2.913	0.00882
Unc5b	1.443	0.01283
Lratd1	2.743	0.02902
Bmp7	1.595	0.03069
Cpe	1.461	0.03666
Prex1	1.529	0.03977
Cfh	1.439	0.04044
Gale	2.681	0.04044
Schip1	2.498	0.04063
Itga10	1.743	0.04334
Gpr153	1.241	0.04874

Figure S2, related to Figure 2. Spatial transcriptomic mapping identifies two cementoblast subtypes

(A,B) Visium HD spatial transcriptomic analysis of P25 mouse mandible. (A): 10X Visium HD data quality. UMI count heatmap (left) and Space Ranger summary metrics (right) for the dataset (8 × 8 μm bins). Key metrics: 216,197 bins under tissue; mean reads/bin 2,332; mean UMIs/bin 426; 18,963 genes detected; sequencing saturation 79%. UMI; unique molecular identifier. (B): The list of DEGs in AC- and CC- cementoblast. (*p*-value < 0.05; log2FC > 1)

Figure S3

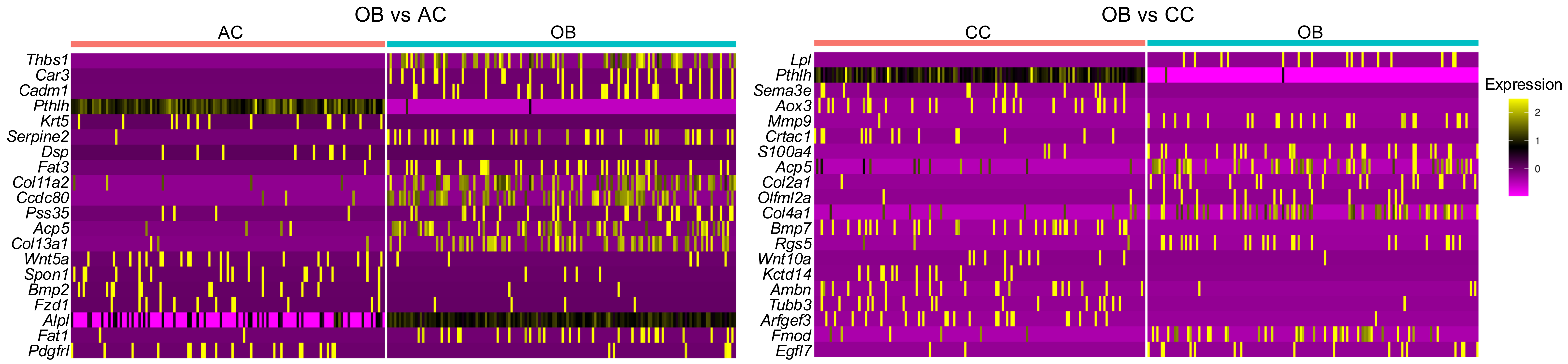

Figure S3, related to Figure 2. Spatial transcriptomic mapping identifies two cementoblast subtypes

Spatial transcriptomic comparison of AC- and CC-forming cementoblasts with alveolar bone osteoblasts. Heatmap of selected differentially expressed genes for OB vs AC comparison (left) and OB vs CC comparison (right).

Figure S4

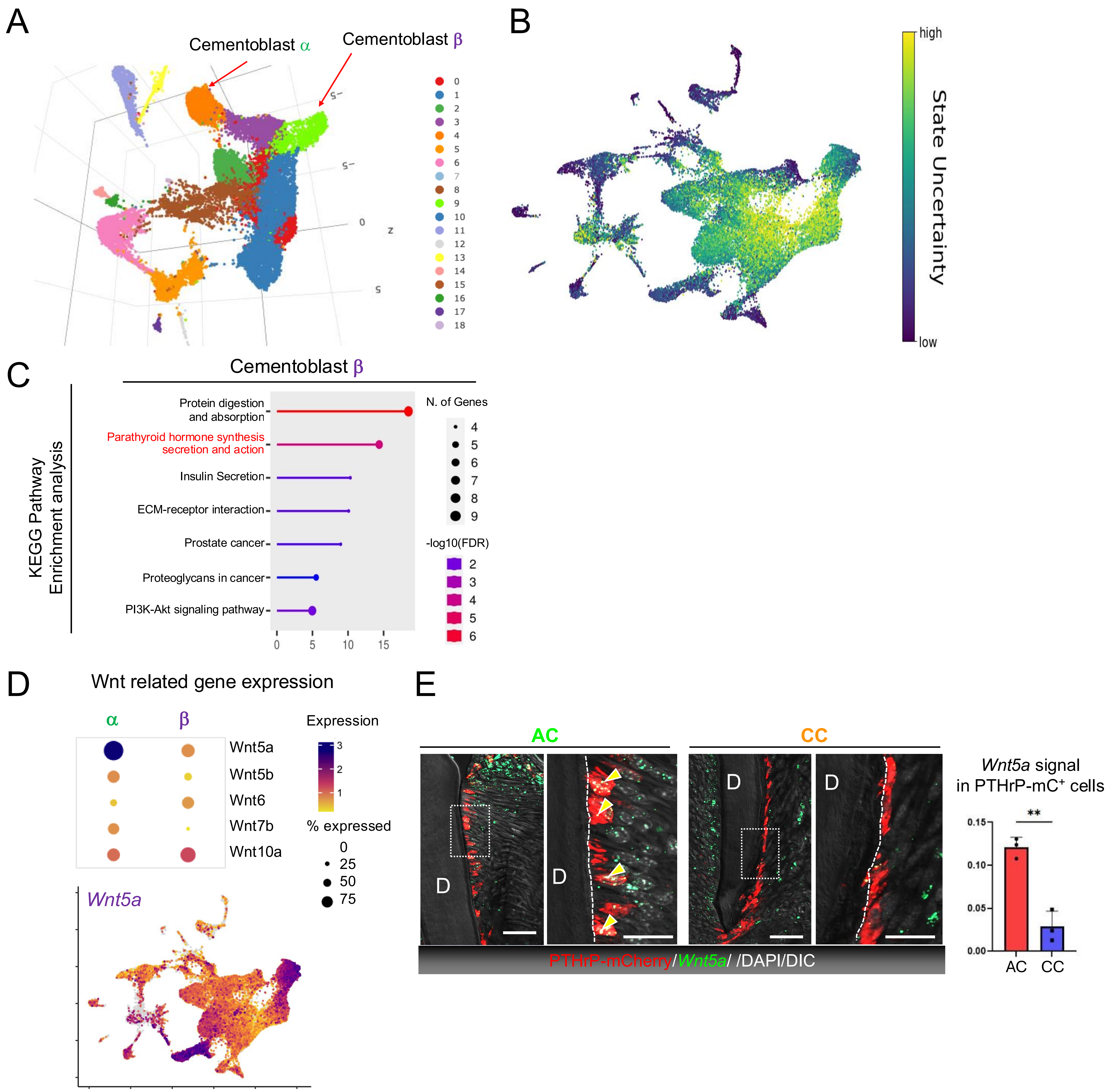

Figure S4, related to Figure 3. Single-cell transcriptomic defines two cementoblast subtypes and their origin

(A) 3D UMAP plot of the integrated single-cell dataset. *Cementoblast α* (Cluster 4, orange) and *Cementoblast β* (Cluster 9, light green) are indicated.

(B) State uncertainty map of the integrated dataset. Cementoblast precursor clusters (0, 1, 3) are enriched for high-uncertainty cells.

(C) KEGG enrichment for Cementoblast *β*-specific DEGs (7 terms significant at FDR < 0.05).

(D) Expression of Wnt-related genes (top) and feature plot of *Wnt5a* (bottom) in the integrated dataset.

(E) RNAscope analysis for *Wnt5a* in AC and CC area of P25 M1, with quantification of *Wnt5a* signal in Pthrp-mCherry^{+} cells. (mean ± s.d.; *n*=3 mice). Red: Pthrp-mCherry, Green: *Wnt5a*, Gray: DIC/DAPI. Scale bars: 50µm (low magnification) and 25 µm (high magnification). D: dentin. ***p*<0.01 (two-tailed Mann–Whitney U test). Representative images of at least three independent biological samples are shown in the figures.

Figure S5

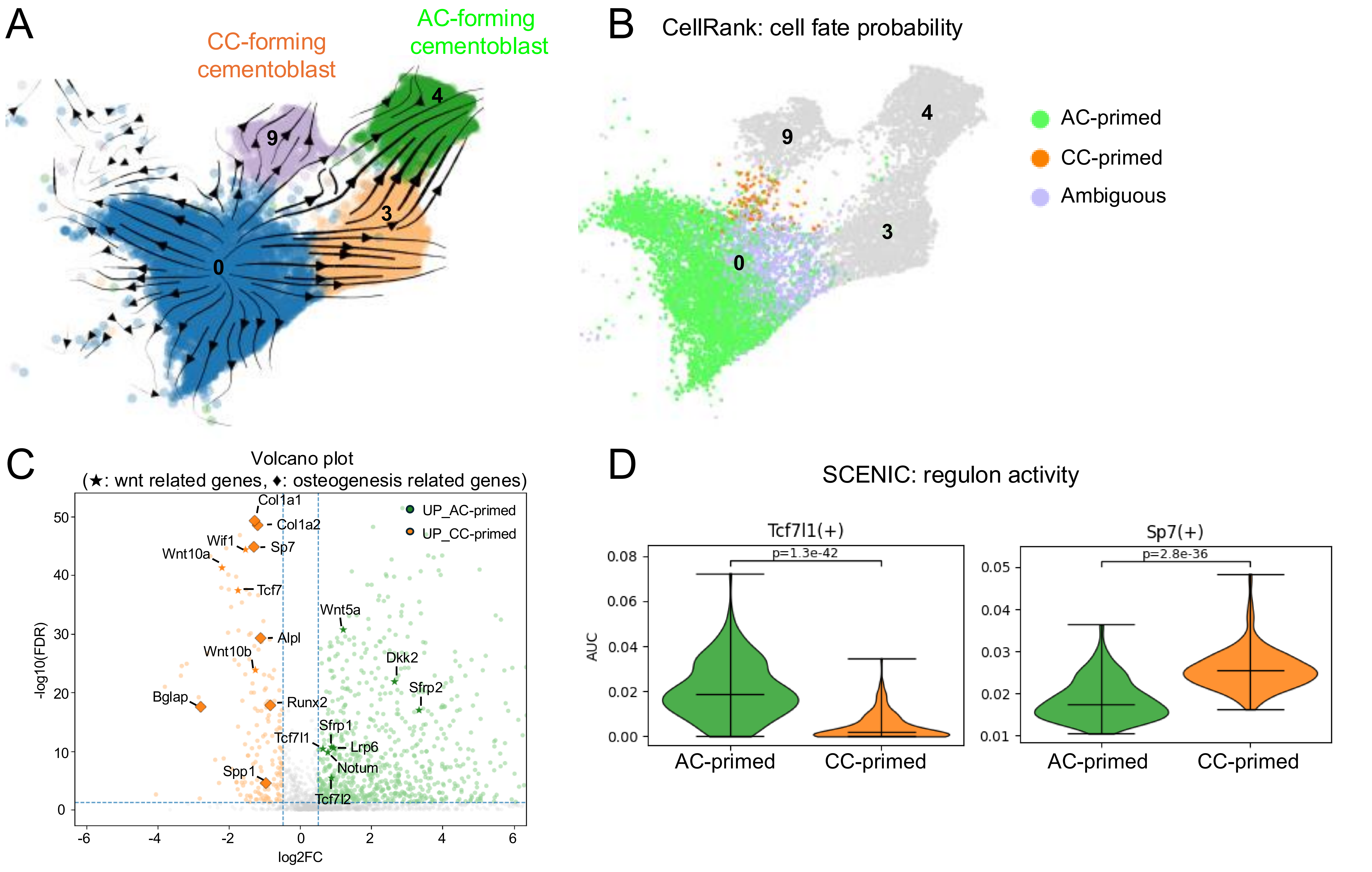

E

Overlap: Tcf7l1 putative targets (top30) ∩ branchpoint DE (top50, AC-up)

gene	regulon_rank	regulon_weight	de_rank	logfoldchanges	pvals_adj	scores
Smoc2	7	1.24	10	5.42	2.1e-41	13.82138729095459
Fbn1	16	1.02	2	4.54	5.8e-49	15.122696876525879
Ebf1	22	0.84	24	5.19	1.4e-35	12.757072448730469
Abcc9	25	0.76	46	2.68	2.3e-29	11.54052734375
Igf1	27	0.69	19	16.33	3.2e-37	13.059803009033203

Figure S5, related to Figure 3. Single-cell transcriptomic defines two cementoblast subtypes and their origin

(A) Integrated RNA velocity vectors and UMAP plot of highlighted Clusters 0, 3, 4, and 9.

(B) CellRank-based fate probabilities computed for Cluster 0, Wif1^{+} origin state, to estimate the probability of commitment toward AC- and CC-forming cementoblasts. Cells in Cluster 0 were categorized as AC-primed, CC-primed, or ambiguous based on fate bias (Δ = p_to_AC − p_to_CC: CellRank-inferred fate probability).

(C) Volcano plot showing DEGs between AC-primed and CC-primed cells. ($|log2FC| > 0.5$, $FDR < 0.05$) The differential expression analysis was performed by comparing equal numbers of cells from the upper and lower tails of the fate-bias score ($n = 200$ per group; AC-biased vs CC-biased). Wnt-related and osteogenesis-related genes are indicated.

(D) SCENIC regulon activity (AUC) in AC- and CC-primed cells.

(E) The overlap between putative Tcf7l1 target genes (top 30 by regulon weight) and AC-upregulated DE genes (top 50) identifies candidate downstream effectors associated with AC priming.

Two-tailed. Two-group comparisons: Mann-Whitney U test; *p*-values are indicated.

Figure S6

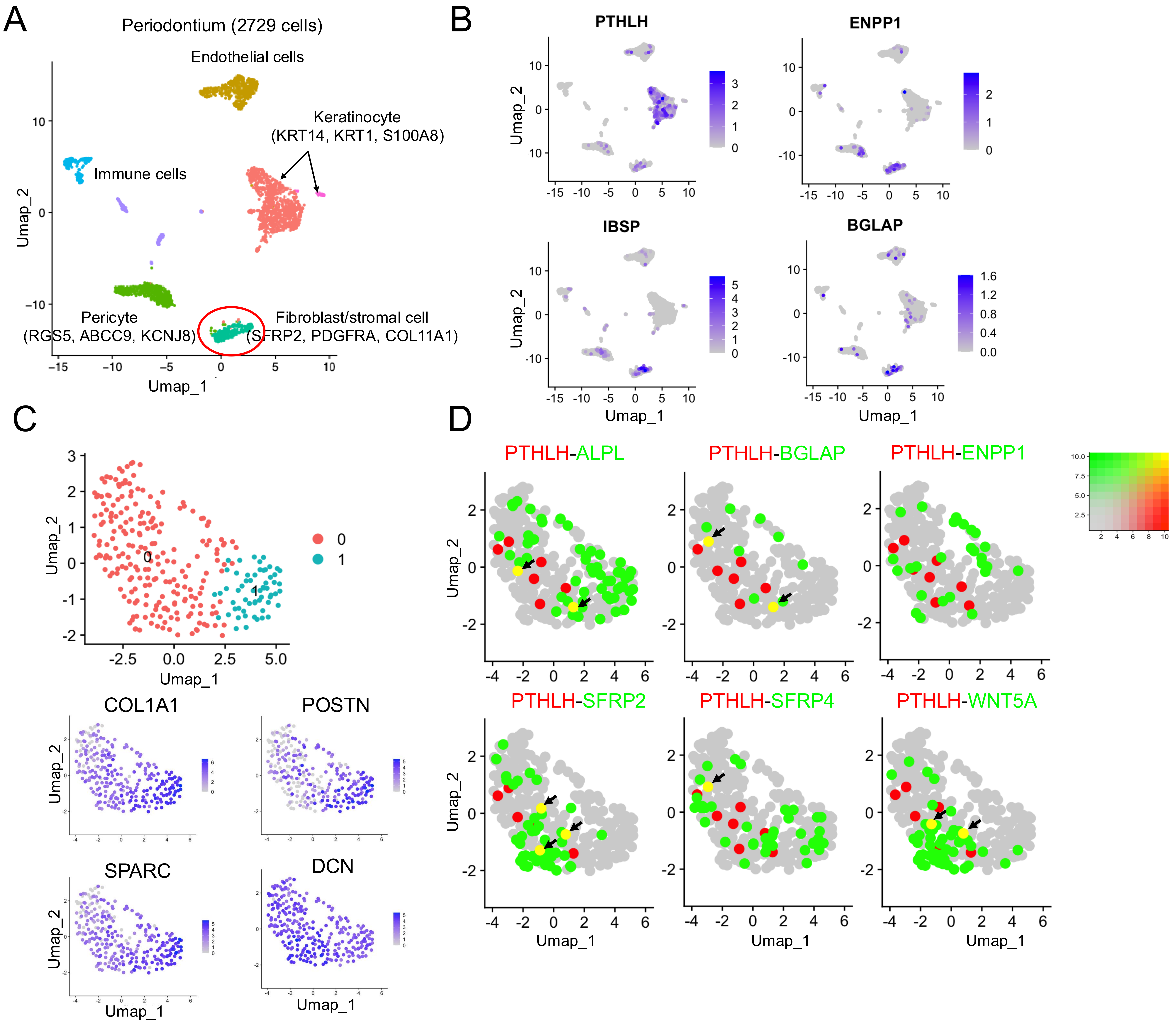

Figure S6, related to Figure 3. Single-cell transcriptomic defines two cementoblast subtypes and their origin

Reanalysis of human periodontium single-cell atlas [GSE161267] to identify a PTHrP^{+} cementoblast subset.

(A) UMAP plot of human periodontium of third molars (2,729 cells).

(B) Feature plots of *PTHLH* (top left), *IBSP* (top right), *BGLAP* (bottom left), *ENPP1* (bottom right).

(C) UMAP plot of the fibroblast/stromal cell subset from (A).

(D) Co-expression of *PTHLH* and *ENPP1* (top left), *BGLAP* (top right), *SFRP2* (bottom left) and *WNT5A* (bottom right). Arrows indicate co-expressing cells.

Figure S7

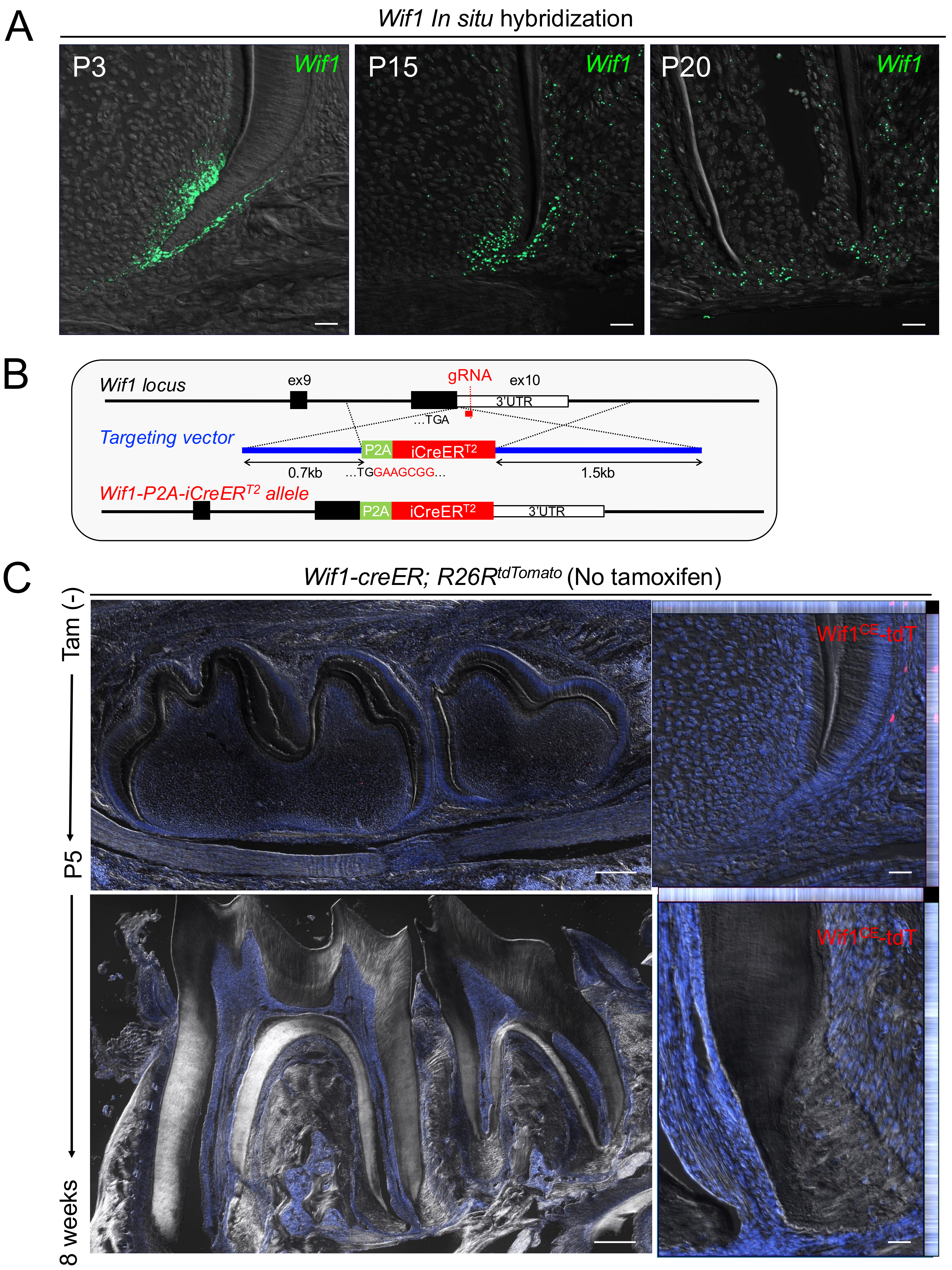

Figure S7, related to Figure 4. Wif1^{+} peri-epithelial apical cells give rise to cementoblasts in tooth root formation

(A) RNAscope analysis for *Wif1* expression during tooth root formation. Enlarged images of HERS surrounding region (P3), apical root front (P15) and root apex (P20). Green: *Wif1* mRNA. Scale bars: 25 µm.
(B) Schematic of the targeting strategy to generate a *Wif1-iCreER* knock-in allele.
(C) No-tamoxifen negative control (*Wif1$^{creER/+}$; R26R^{tdTomato}*). M1 sections collected without tamoxifen at P5 (top) and 8 weeks (bottom). No tdT signal was detected in the peri-epithelial apical mesenchyme, indicating minimal leakiness of the *Wif1-creER* allele. Red: Wif1^{CE}-tdT, Gray: DIC/DAPI. Scale bars; 200 µm (left), 25 µm (right). Representative images of at least three independent biological samples are shown in the figures.

Figure S8

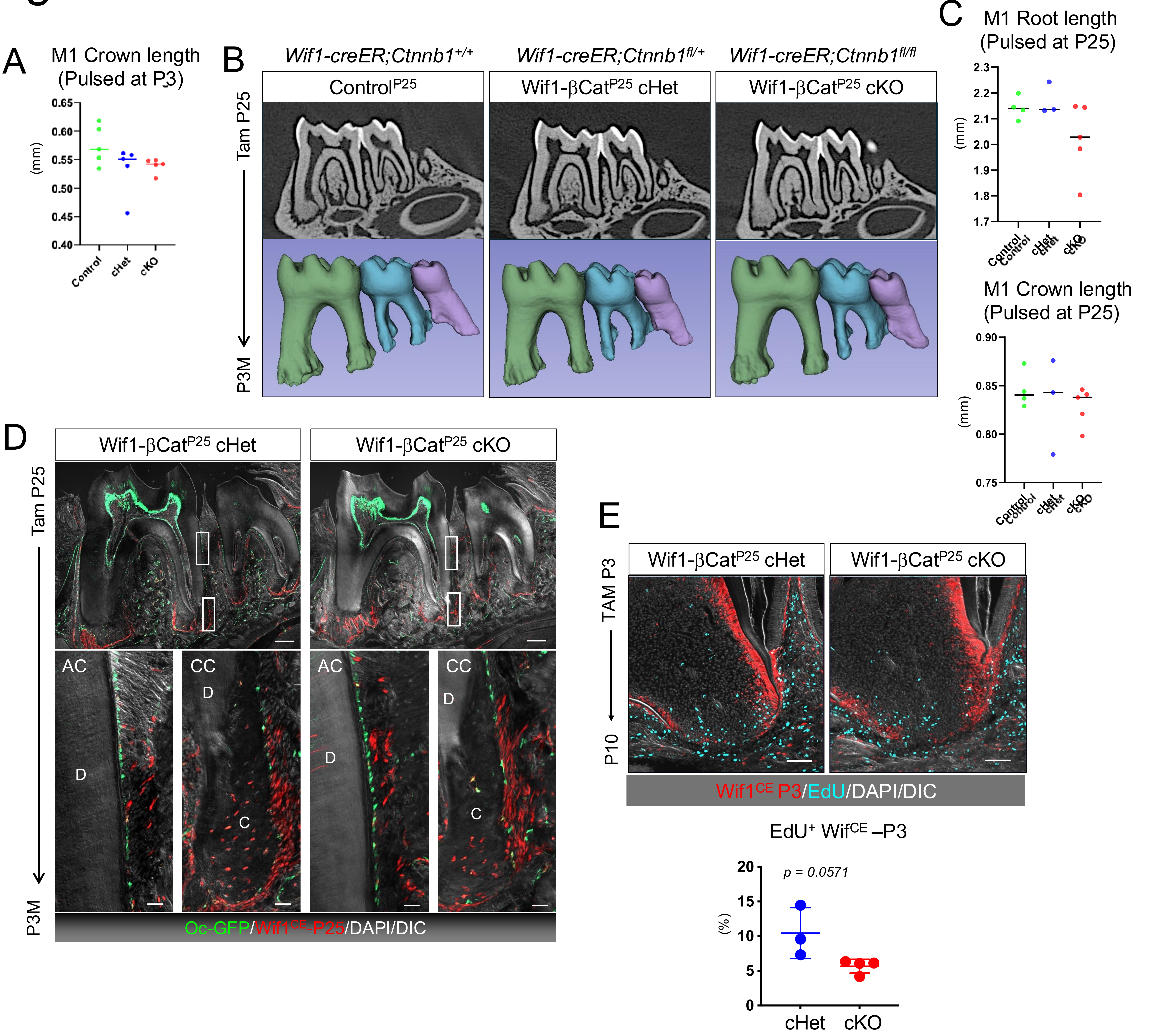

Figure S8, R/T Figure 5. Canonical Wnt signaling dependency of acellular cementum formation by Wif1⁺ cells

(A) Quantification of M1 crown length at 3 months of age (pulsed at P3). (mean ± s.d.; *n*=5 mice, each group).

(B-D) Later-stage canonical Wnt signaling inactivation in *Wif1*⁺ cells. P25-pulsed Wif1-βCat cont-P25, Wif1-βCat cHet-P25 and Wif1-βCat cKO-P25 littermates were chased to 3 months. (B): 3D-microCT cross-sectional slice (top) and 3D-rendered image (bottom) of each group. (C): Quantification of M1 root length and crown length. (mean ± s.d.; Control: *n*=4 mice, cHet: *n*=3 mice, cKO: *n*=5 mice). (D): Lineage tracing. Low magnification image of M1 (top) and enlarged images of AC (left), CC (right) of Wif1-βCat cHet-P25 and Wif1-βCat cKO-P25. Scale bars; 200 µm (low magnification) and 25 µm (high magnification). Red; Wif1^{CE}-P25, Green; Oc-GFP; Gray; DIC/DAPI.

(E) Cell proliferation. EdU assays after 7 days of chase at P10 (pulsed at P3). EdU was administered twice (6 and 3 hrs) before analysis. Quantification of EdU^{+}Wif1^{CE}-tdT^{+} cells per Wif1^{CE}-tdT^{+} cells is shown in lower graph. (mean ± s.d.; *n*=3 mice, each group) Scale bars; 50 µm. Red: Wif1^{CE}-P3, Cyan: EdU; Gray: DIC/DAPI. D: dentin, C: cementum. Two-group comparisons: Mann-Whitney's *U*-test. *n*≥3 groups: one-way ANOVA with Tukey's multiple-comparisons test. Representative images from at least three biological replicates are shown in the figures.

Figure S9

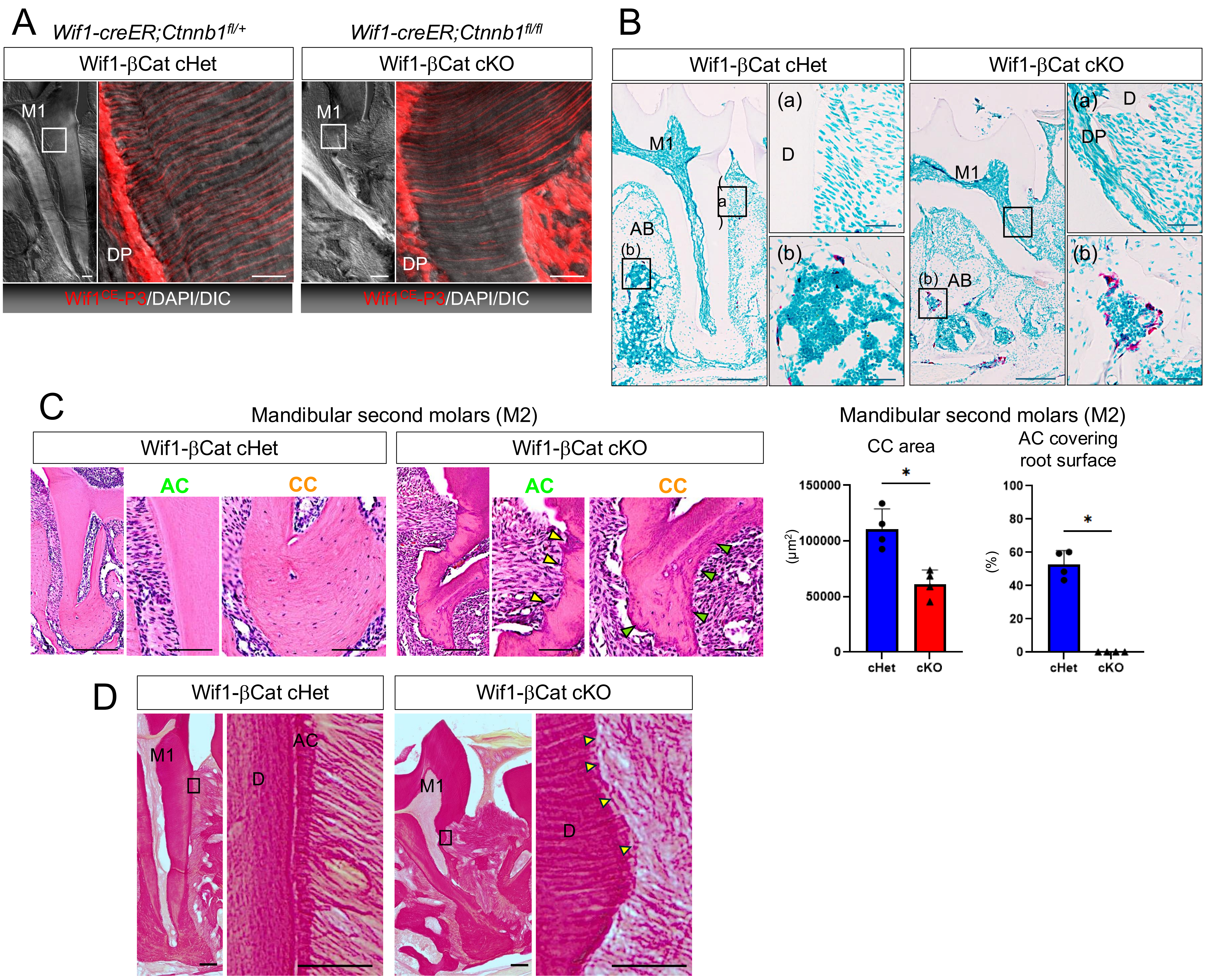

Figure S9, R/T Figure 5. Canonical Wnt signaling dependency of acellular cementum formation by Wif1^{+} cells

First molar (M1) from Wif1-βCat cHet and Wif1-βCat cKO mice pulsed at P3 and analyzed after 3-month of chase.

(A) Dentinal tubule organization, M1. Representative fluorescent images. Boxed regions in the low-magnification images (left) are shown at higher magnification (right) to visualize dentinal tubules labeled by tdTomato. Red; Wif1^{CE}-P3. Scale bars: 100 μm (low magnification) and 25 μm (high magnification). Scale bars: 25μm. DP: dental pulp.

(B) TRAP staining, M1. Representative TRAP staining of mandibular molar sections. Higher magnification images of the cervical root surface (a) and alveolar bone (b). Scale bars: 250μm (low magnification), 50μm (high magnification). M1: first molar, AB: alveolar bone, D: dentin, DP: dental pulp.

(C) Second molar (M2) AC coverage. Right: quantification of AC coverage per M2 mesial root surface (left) and CC area of M2 mesial root. (mean ± s.d.; n=4 mice, each group) Scale bars: 250μm (low magnification), 50μm (high magnification). *p<0.05 (two-tailed Mann–Whitney U test).

(D) Picrosirius Red (PSR) staining, M1. High-magnification views of M1 cementum-PDL interface. cHet: dense PDL fibers (Sharpey's fibers) insert into AC. cKO: AC is absent with markedly reduced fiber insertion. Yellow arrowheads indicate the absence of AC. Scale bars: 100 μm (low magnification) and 25 μm (high magnification). D, dentin; M1, first molar; AC, acellular cementum. Representative images from at least three biological replicates are shown in the figures.

Table S1

Cluster	Annotation	Key markers used for annotation	Top markers
0	Cementoblast precursor	Ogn, Cxcl12, Sfrp1, Igf1, Igfbp4	Ogn, Igfbp4, Igf1, Fbln1, Gpx3, Mfap4
1	Cementoblast precursor	Pcp4, Vcan, Bambi, Crabp1, Id1	Pcp4, Hmgn3, Ptn, Vcan, Bambi, Id1
2	Periapical cells	Hand2, Nts, Alx1, Tnn, Csrp2	Csrp2, Nts, Hand2, Tnn, Lrrc17, Cald1
3	Cementoblast precursor	Alpl, Col16a1, Thbs4, Rora, Col1a1/Col1a2	Col1a2, Col3a1, Sparc, Col1a1, Col16a1, Rora
4	Cementoblast	Dmp1, Enpp1, Ibsp, Wnt5a, Ank	Ibsp, Ank, Timp3, Bmp3, Aqp1, Sparcl1
5	Mitochondrial transcript enriched	mt-Nd4, mt-Atp6, mt-Co3, mt-Co2, mt-Cytb	mt-Nd4, mt-Atp6, mt-Co3, mt-Nd2, mt-Co2, mt-Cytb
6	Epithelial cells	Krt5, Krt14, Trp63, Epcam, Krt17	Krt5, Krt14, Sfn, Dsp, Perp, Fabp5
7	Periapical cells	Itgbl1, Htra1, Col18a1, Col4a1, Serpine2	Htra1, Itgbl1, Wnt5a, Spon1, Col4a1, Col18a1
8	Cycling/proliferating cells	Mki67, Top2a, Stmn1, Hmgb2	H2afz, Hmgb2, Stmn1, Ran, Smc2, Hmgb1
9	Cementoblast	Dmp1, Ifitm5, Bglap, Phex	Col11a2, Col1a1, Smpd3, Ifitm5, Bglap, Sparc
10	Periapical cells	Mgp, Nrp1, Prrx1, Ebf1, Cygb	Mgp, Ebf1, Fbn1, Nrp1, Col4a1, Prrx1
11	Hematopoietic/immune cells	Ptprc, Lyz2, Csf1r, Tyrobp, Fcer1g	Fcer1g, Tmsb4x, Tyrobp, Laptm5, Ctss, Ftl1
12	Epithelial cels	Trp63, Pitx2, Wnt4, Dsc3	Wnt4, Dsc3, Trp63, Fxyd3, Runx1, Pitx2
13	Hematopoietic/immune cells	Ptprc, S100a8, S100a9, Ltf	S100a8, Lcp1, S100a9, Tyrobp, Cd52, Tmsb4x
14	Epithelial cells	Krt5, Krt14, Odam, Krt17	Krt14, Odam, Krt5, Perp, Spint2, Sfn
15	Hematopoietic/immune cells	Hba-a1, Hbb-bs, Alas2	Hba-a1, Hbb-bs, Hba-a2, Hbb-bt, Alas2, Snca
16	Hematopoietic/immune cells	Ptprc, Cd79a, Cd79b, Ebf1, Vpreb3	Cd79b, Cd79a, Ebf1, Vpreb3, Ptprcap, Chchd10
17	Endothelial cells	Pecam1, Cdh5, Kdr, Flt1, Vwf, Egfl7	Egfl7, Pecam1, Cdh5, Ecscr, Col4a1, Ramp2
18	Schwann/glial cells	Plp1, Mpz, Pmp22, S100b	Plp1, Dbi, Itih5, Gpm6b, Cryab, Lgi4
