# Supplementary material for: Wnt-dependent ontogeny of acellular cementum-forming cementoblasts on the tooth root surface
Source: Nat Commun. 2026 May 13;17:6362. doi: 10.1038/s41467-026-72712-1 (PMC13376515; doi:10.1038/s41467-026-72712-1)
Supplement: Supplementary file 3 — Reporting Summary [file 41467_2026_72712_MOESM3_ESM.pdf]

nature portfolio

Corresponding author(s): Wanida Ono

Last updated by author(s): Apr 20, 2026

Reporting Summary

Nature Portfolio wishes to improve the reproducibility of the work that we publish. This form provides structure for consistency and transparency in reporting. For further information on Nature Portfolio policies, see our Editorial Policies and the Editorial Policy Checklist.

Statistics

For all statistical analyses, confirm that the following items are present in the figure legend, table legend, main text, or Methods section.

n/a	Confirmed	
☐	☒	The exact sample size (*n*) for each experimental group/condition, given as a discrete number and unit of measurement
☐	☒	A statement on whether measurements were taken from distinct samples or whether the same sample was measured repeatedly
☐	☒	The statistical test(s) used AND whether they are one- or two-sided<br>*Only common tests should be described solely by name; describe more complex techniques in the Methods section.*
☒	☐	A description of all covariates tested
☐	☒	A description of any assumptions or corrections, such as tests of normality and adjustment for multiple comparisons
☐	☒	A full description of the statistical parameters including central tendency (e.g. means) or other basic estimates (e.g. regression coefficient) AND variation (e.g. standard deviation) or associated estimates of uncertainty (e.g. confidence intervals)
☐	☒	For null hypothesis testing, the test statistic (e.g. *F*, *t*, *r*) with confidence intervals, effect sizes, degrees of freedom and *P* value noted<br>*Give P values as exact values whenever suitable.*
☒	☐	For Bayesian analysis, information on the choice of priors and Markov chain Monte Carlo settings
☒	☐	For hierarchical and complex designs, identification of the appropriate level for tests and full reporting of outcomes
☒	☐	Estimates of effect sizes (e.g. Cohen's *d*, Pearson's *r*), indicating how they were calculated

Our web collection on statistics for biologists contains articles on many of the points above.

Software and code

Policy information about availability of computer code

Data collection	ZEN 3 (blue edition, ZEISS), Space Ranger v3 (10x Genomics Cloud analysis), Cell Ranger 6.1 (10X Genomics), Scanco µCT Evaluation Program (SCANCO Medical)
Data analysis	ZEN 3 (blue edition, ZEISS), ImageJ2 v2, GraphPad Prism v10.3.0, Loupe Browser v8, rliger v2.2.1, Qupath v0.5.1

For manuscripts utilizing custom algorithms or software that are central to the research but not yet described in published literature, software must be made available to editors and reviewers. We strongly encourage code deposition in a community repository (e.g. GitHub). See the Nature Portfolio guidelines for submitting code & software for further information.

Data

Policy information about availability of data

All manuscripts must include a data availability statement. This statement should provide the following information, where applicable:

- Accession codes, unique identifiers, or web links for publicly available datasets
- A description of any restrictions on data availability
- For clinical datasets or third party data, please ensure that the statement adheres to our policy

The spatial transcriptomic analysis and single-cell RNA-seq data presented herein have been deposited in the National Center for Biotechnology Information (NCBI)'s Gene Expression Omnibus (GEO),and are accessible through the GEOSeries accession number, SuperSeries GSE313653 (spatial transcriptomic analysis), and SuperSeries GSE313714 (single-cell RNAseq) including GSM9372961, GSM9372962 and GSM9372963.

Research involving human participants, their data, or biological material

Policy information about studies with human participants or human data. See also policy information about sex, gender (identity/presentation), and sexual orientation and race, ethnicity and racism.

Reporting on sex and gender	N/A
Reporting on race, ethnicity, or other socially relevant groupings	N/A
Population characteristics	N/A
Recruitment	N/A
Ethics oversight	N/A

Note that full information on the approval of the study protocol must also be provided in the manuscript.

Field-specific reporting

Please select the one below that is the best fit for your research. If you are not sure, read the appropriate sections before making your selection.

[x] Life sciences [ ] Behavioural & social sciences [ ] Ecological, evolutionary & environmental sciences

For a reference copy of the document with all sections, see nature.com/documents/nr-reporting-summary-flat.pdf

Life sciences study design

All studies must disclose on these points even when the disclosure is negative.

Sample size	Sample sizes were based on prior experience with similar experiments in the field (Nat Commun 16(1):6061, 2025, Nat Communication 16 (1):5510, 2025) and practical considerations. No statistical method was used to predetermine sample size. The number of biological replicates for each experiment is reported in the figure legends.
Data exclusions	Some data were excluded based on pre-established quality-control criteria and only for technical or health-related reasons unrelated to the experimental intervention. Exclusions included failures in confirming correct genotypes or birth dates, poor tissue quality/processing failures, or spontaneous health issues (e.g., malnutrition). In all analyses, we used littermate controls with corresponding genotypes.
Replication	For all data presented in the manuscript, we examined at least three independent biological samples (three different mice) to ensure the reproducibility. Representative images shown are from at least 3 mice per genotype/condition.
Randomization	Randomization was not applicable because animals were assigned to groups based on genotype (littermates).
Blinding	Investigators were not fully blinded to group allocation during experiments and outcome assessment because group assignment was determined by genotyping prior to the start of experiments and animals were handled accordingly during housing, tissue collection, sample preparation, and data acquisition. However, samples were assigned coded numeric identifiers to reduce the ability to infer genotype from sample IDs during downstream quantification. Quantification (measuring and counting) was performed using the same criteria across all samples without preferential attention to group identity.

Reporting for specific materials, systems and methods

We require information from authors about some types of materials, experimental systems and methods used in many studies. Here, indicate whether each material, system or method listed is relevant to your study. If you are not sure if a list item applies to your research, read the appropriate section before selecting a response.

Materials & experimental systems

n/a	Involved in the study	
[ ]	[x]	Antibodies
[x]	[ ]	Eukaryotic cell lines
[x]	[ ]	Palaeontology and archaeology
[ ]	[x]	Animals and other organisms
[x]	[ ]	Clinical data
[x]	[ ]	Dual use research of concern
[x]	[ ]	Plants

Methods

n/a	Involved in the study	
[x]	[ ]	ChIP-seq
[x]	[ ]	Flow cytometry
[x]	[ ]	MRI-based neuroimaging

Antibodies

Antibodies used	Rabbit anti-POSTN polyclonal antibody (1:500, EMD-Millipore, ABT280) and rabbit anti-cytokeratin 5 (CK5) polyclonal antibody (1:200, Abcam, ab24647)
Validation	All antibodies used in this study are commercially available and were used according to the manufacturers' recommendations. Validation information is provided by the suppliers (datasheets). Specific staining patterns were consistent with expected tissue localization and published reports.

Animals and other research organisms

Policy information about studies involving animals; ARRIVE guidelines recommended for reporting animal research, and Sex and Gender in Research

Laboratory animals	We used genetically modified mice (mus musculus) for this study. Most of the mouse line have been backcrossed to C57BL/6 background. Mice with both sexes were used throughout their lifespan (up to 2 years of age). Mouse strains used in the study were as following: Wif1-P2A-iCreER knock-in mice were generated by CRISPR/Cas9-mediated insertion of iCreERT2 upstream of the Wif1 stop codon, allowing inducible Cre activity under endogenous Wif1 regulatory control. The founders were confirmed by PCR genotyping and bred for germline transmission. Pthrp-mCherry knock-in reporter mice have been previously described (JAX032872). Rosa26-CAG-loxP-stop-loxP-tdTomato (Ai14: R26RtdTomato, JAX007914), Osteocalcin (3.8kb)-GFP (JAX017469) and Ctnnb1-floxed (JAX004152) mice were acquired from the Jackson laboratory. Animal rooms were climate controlled to provide temperatures of 22-23°C, 40-65% of humidity on a 12 h light/dark cycle.
Wild animals	No wild animals were used in this study.
Reporting on sex	Data were not disaggregated by sex. The sample numbers reported in this Reporting Summary include both male and female mice. Sex was recorded for postnatal animals, but data from both sexes were pooled for analysis because the study was not powered or designed to test sex-specific effects.
Field-collected samples	No field collected samples were used in the study.
Ethics oversight	All procedures were conducted in compliance with the Guidelines for the Care and Use of Laboratory Animals approved by the University of Texas Health Science Center at Houston's Animal Welfare Committee (AWC), protocol AWC-21-0070 and AWC-24-0075.

Note that full information on the approval of the study protocol must also be provided in the manuscript.

Plants

Seed stocks	*Report on the source of all seed stocks or other plant material used. If applicable, state the seed stock centre and catalogue number. If plant specimens were collected from the field, describe the collection location, date and sampling procedures.*
Novel plant genotypes	*Describe the methods by which all novel plant genotypes were produced. This includes those generated by transgenic approaches, gene editing, chemical/radiation-based mutagenesis and hybridization. For transgenic lines, describe the transformation method, the number of independent lines analyzed and the generation upon which experiments were performed. For gene-edited lines, describe the editor used, the endogenous sequence targeted for editing, the targeting guide RNA sequence (if applicable) and how the editor was applied.*
Authentication	*Describe any authentication procedures for each seed stock used or novel genotype generated. Describe any experiments used to assess the effect of a mutation and, where applicable, how potential secondary effects (e.g. second site T-DNA insertions, mosiacism, off-target gene editing) were examined.*
