# Supplementary material for: Wnt-dependent ontogeny of acellular cementum-forming cementoblasts on the tooth root surface
Source: Nat Commun. 2026 May 13;17:6362. doi: 10.1038/s41467-026-72712-1 (PMC13376515; doi:10.1038/s41467-026-72712-1)

nature portfolio

Peer Review File

Wnt-dependent ontogeny of acellular cementum-forming cementoblasts on the tooth root surface

Corresponding Author: Dr Wanida Ono

This file contains all reviewer reports in order by version, followed by all author rebuttals in order by version.

Version 0:

Reviewer comments:

Reviewer #1

(Remarks to the Author)
The manuscript, "Wnt-dependent ontogeny of acellular cementum-forming cementoblasts on the tooth root surface," by Dr Ono and colleagues, addresses longstanding and unsettled questions in dental development: What is the origin of the cementoblast cell population that directs cementogenesis on the tooth root surface and are acellular and cellular cementum-associated cementoblasts the same or different cell types? These questions are key for understanding tooth development and potential for periodontal repair and regeneration. The authors use a variety of approaches to address the questions, including transgenic mouse models, lineage tracing, and RNA sequencing. The experimental design and interpretation are appropriate, with some comments below. Writing is clear and excellent, and figures are high quality. Progress is made in addressing the main questions and a substantial quantity of additional data are yielded which are valuable for dental and skeletal researchers, making the study multifaceted and important. Some moderate concerns and minor suggestions are summarized below, which are important to address to clarify the approach and interpretation of the findings.

Moderate concerns

1) Authors do a nice job of comparing cementoblasts of acellular vs. cellular cementum throughout the course of this paper. Historically, cementoblasts have been traditionally compared to osteoblasts (as in the excellent review by Bosshardt, J Dent Res, 2005). It is a bit of a missed opportunity to not update this comparison in this study. Even if osteoblasts have not been reported among the cell populations here, can authors add context to their findings in the two (or more) cementoblast populations vs. osteoblasts in their previous work or in the reports of others?

2) Why do the authors tend to keep using the term “peri-epithelial” cells in the Results and Discussion? Is it to make the point they are not epithelial in lineage and are not part of the wider follicle/PDL cell population? This is a point of minor confusion that could perhaps be clarified for readers to understand the importance of the distinction.

3) The stepwise discoveries that identify Wif1 as a cementoblast-selective factor are logical and clearly explained. However, Wif1 is reportedly expressed in other cells and tissues, potentially limiting its usefulness as a selective marker or molecular target for selectively affecting cementoblasts. Some publications suggest Wif1 in epithelium and the authors’ own results here suggest odontoblast/dentin are affected by using Wif1-Cre.

4) Is it possible the cells analyzed by scRNA-seq around the apical root included late cementoblasts/early cementocytes? Dmp1 and Phex expression may suggest this possibility, though there are some different accounts for Dmp1- and Phex-expressivity in acellular vs. cellular cementum cells in the literature.

5) The strategy here depends heavily on Pthlh/Pthrp as an accurate marker for acellular and cellular cementum cementoblasts and is derived from prior work from the group. Can the authors comment on the degree of specificity of Pthlh/Pthrp in cementoblasts and not in surrounding cells that can potentially contaminate cell analyses, e.g. PDL fibroblasts or pulp/odontoblast cells? Is there a possibility of missing some portion of cementoblasts that are weak or negative for Pthlh/Pthrp?

6) According to authors, the phenotype of the Wif1-Cre:Ctnnb1 conditional KO mice indicates defects in dentinogenesis. In that case, extreme caution must be used because any dentin defect can cause secondary developmental defects in

cementum formation that may not reflect direct effects on cementoblasts, but rather defects in the dentin scaffold onto which cementum is deposited.

7) The appearance of Wif1-Cre:Ctnnb1 conditional KO mouse molars (especially first molars) in Figure 5B strongly resembles cervical root resorption, which can accompany acellular cementum defects. Because the age here is advanced, it is difficult to confirm if this is a developmental phenotype in dentin and cementum or aggressive resorption of roots. Developmental defects can look like thin dentin and cementum, lack of cementum by histology stains, and PDL detachment due to poor integration of Sharpey's fibers; however, this root surface appears to be scalloped, reminiscent of resorption. This is important to clarify to guide the interpretation.

Minor suggestions

1) The "coronal" portion of the root is described throughout the manuscript as the location of acellular cementum. I think this may cause confusion. I understand what the authors intend- this type of cementum is located in the coronal direction on the tooth, but it is not actually coronally located, which would typically mean covering the crown. Because there are forms of coronal cementum that actually do cover tooth crowns in some species (e.g. horses, cows, elephants, and other grass-eating species), I think this could be misinterpreted. Oftentimes, acellular cementum is described as located on the cervical ("neck") portion of the tooth or tooth root and I think this is a more accurate description.

2) Introduction – page 3 – line 55: The authors describe that acellular cementum lacks "intrinsic regenerative potential," which I think is confusing or slightly inaccurate. Many papers describe repair cementum in the region of acellular cementum, thought to be produced by cementoblasts. While repair cementum is often described as more cellular in appearance and with unclear bonding to dentin and PDL, it does show repair capacity for the tissue.

3) Figure 1: This SEM exploration of acellular vs. cellular cementum structure is a bit superficial and does not break new ground- these types of images and descriptions have appeared in many past papers. It is a nice way to ease into the more substantial portions of the paper and introduce non-dental readers to the anatomy and appearance, but does not add much overall to the advances of the study.

4) Results – page 10 – line 258: HERS is mentioned in the context of 9 week old animals (unless I am misunderstanding), but HERS would no longer be present at this age that is well past the root development stage. There may be epithelial rests of Malassez, but not HERS proper. Please clarify.

5) Figure 1A and C: The tooth schematic does not illustrate acellular vs. cellular cementum very clearly, or just cementum from dentin and PDL, for that matter.

Reviewer #2

(Remarks to the Author)
This study provides fundamental insights into acellular cementum (AC) development, essential for periodontal attachment. Using integrated spatial/single-cell transcriptomics and Wif1-creER lineage tracing, the authors: (1) identify AC-forming cementoblasts as a unique noncanonical mineralizing population distinct from cellular cementum (CC)-forming cementoblasts, characterized by specific cell-matrix and Wnt signaling signatures; (2) define their exclusive origin from Wif1+ peri-epithelial apical precursors via canonical Wnt activation; and (3) establish canonical Wnt as a molecular switch directing Wif1+ precursors toward the AC lineage, revealing how this pathway diversifies to generate distinct root mineralized tissues (AC vs. CC). Significantly, this work resolves the long-debated origin of AC cementoblasts, establishes a crucial developmental/molecular dichotomy between AC and CC formation, and proposes a novel targeted strategy (e.g., activating Wnt in progenitors) to regenerate AC and restore periodontal attachment lost to disease, addressing a key clinical challenge. This study is recommended for publication.

Reviewer #3

(Remarks to the Author)
In this study, Komori et al. employed spatial transcriptomics, single-cell RNA sequencing, lineage tracing, and genetic loss-of-function analyses to identify Wif1^{+} peri-epithelial apical mesenchymal cells as a primary source of acellular cementum (AC)-forming cementoblasts. The authors demonstrated that specification of this lineage is strictly dependent on canonical Wnt/β-catenin signaling during early root development, and described the molecular and developmental distinction between AC-forming and cellular cementum (CC)-forming cementoblasts. Based on these findings, they proposed that targeting this Wnt-dependent pathway could represent a potential strategy for regenerating AC to restore periodontal attachment.

Major points:
1. Mechanistic insights remain to be further explored. The authors proposed Wnt signaling as a binary switch but failed to delineate the downstream transcriptional network executing the AC program. The key target genes of β-catenin-mediated signaling in Wif1^{+} cells remain unidentified. Furthermore, the nature of the proposed "Wnt-independent" pathway for CC formation is unexplored, at least it should be discussed.
2. Functional validation needs to be further improved. The phenotypic analysis following β-catenin deletion is morphology-oriented and quite descriptive (eg. absence of AC, cell fate shift). Besides M1 molar, M2/M3 molars also developed abnormally and should be described. In addition, biomechanical assessment of the periodontium (e.g., tensile strength, tooth

mobility) could be carried out in the mutant models to prove that AC absence directly compromises periodontal attachment function.
3. The work is exclusively based on mouse developmental models. The direct relevance to human AC biology, homeostasis, and the pathological/regenerative process during adult periodontitis is uncertain. It is suggested to supplemented experiments of human samples to enhance the clinical application value.

Minor points:
1. The vertical axis of the column chart is inconsistent among different figures. A consistent and appropriate scale is recommended.
2. For single-cell RNA-seq analysis, specific marker genes used to annotate each cluster in feature plots or dot plots should be clearly stated.
3. For scRNA-seq analysis in Figure 3, three key improvements are needed: i) Expression scale bars are missing; ii) Specific cell clusters/subtypes should be clearly defined in Figure 3B; iii) In Figure 3C, Wif1 expression used a different color scheme without any scale bar, better change to the same color scheme as other genes within this panel.
4. The AC was an avascular, cell-free matrix tissue. However, nuclear signals are perceptible within the mineralized structure in some immunofluorescence images (e.g., Fig. S3E). These signals should be further verified.

Version 1:

Reviewer comments:

Reviewer #1

(Remarks to the Author)
The revised manuscript, “Wnt-dependent ontogeny of acellular cementum-forming cementoblasts on the tooth root surface,” by Dr. Ono and colleagues, represents a substantial and impressive advance over the original submission. The authors have done a spectacular job addressing all reviewer comments with thoroughness, rigor, and clarity. The study tackles longstanding and fundamental questions in periodontal and dental developmental biology—specifically, the origin of cementoblast populations responsible for cementogenesis on the tooth root surface and the distinction between acellular and cellular cementum–associated cementoblasts. These questions are central to advancing our understanding of tooth development and have important implications for periodontal repair and regenerative strategies. Through an elegant combination of transgenic mouse models, lineage tracing, and RNA sequencing, the authors provide well-supported insights that significantly refine the field’s conceptual framework and help define critical remaining gaps in periodontal biology. The experimental design is excellent, the data are carefully interpreted, and the conclusions are well justified by the results. Data now include comparisons to osteoblasts, additional mechanistic-minded analysis of sequencing data, additional analyses of dentin and potential for root resorption in one of the models, and extrapolation to human data. The manuscript is clearly written, the figures are of high quality, and the responses to prior critiques have strengthened both the presentation and the scientific impact of the work. Overall, this revised version is a multifaceted, rigorous, and important contribution that will be of broad interest to dental, periodontal, and skeletal biology researchers.

Reviewer #3

(Remarks to the Author)
The authors have nicely addressed all my concerns. The manuscript is now ready to be accepted for publication.

Open Access This Peer Review File is licensed under a Creative Commons Attribution 4.0 International License, which permits use, sharing, adaptation, distribution and reproduction in any medium or format, as long as you give appropriate credit to the original author(s) and the source, provide a link to the Creative Commons license, and indicate if changes were made.
In cases where reviewers are anonymous, credit should be given to 'Anonymous Referee' and the source.
The images or other third party material in this Peer Review File are included in the article's Creative Commons license, unless indicated otherwise in a credit line to the material. If material is not included in the article's Creative Commons license and your intended use is not permitted by statutory regulation or exceeds the permitted use, you will need to obtain permission directly from the copyright holder.
To view a copy of this license, visit https://creativecommons.org/licenses/by/4.0/

Response to Reviewers

We thank the reviewers for their positive assessment and constructive criticisms. Below, we have addressed all the reviewers' points with additional data.

The newly added data include the analysis of alveolar bone osteoblasts on our 10X Visium HD spatial transcriptomics and their comparison to cementoblasts (**Rev.1**), the assessment of osteoclasts as a potential cause of cervical root abnormalities (**Rev.1**), the in-depth analysis (branchpoint, SCENIC) of our integrated scRNA-seq data to define the lineage-determining cues toward acellular cementum-forming cementoblast fate (**Rev.3**), and the analysis of the human periodontal scRNA-seq data from third molars to confirm the presence of PTHrP^{+} cementoblasts (**Rev.3**).

Please see below for point-by-point responses to each reviewer's comments. The revised portions of the main text have been highlighted in red letters. We believe that our manuscript has now been substantially improved.

Reviewer #1:

The manuscript, "Wnt-dependent ontogeny of acellular cementum-forming cementoblasts on the tooth root surface," by Dr Ono and colleagues, addresses longstanding and unsettled questions in dental development: What is the origin of the cementoblast cell population that directs cementogenesis on the tooth root surface and are acellular and cellular cementum-associated cementoblasts the same or different cell types? These questions are key for understanding tooth development and potential for periodontal repair and regeneration. The authors use a variety of approaches to address the questions, including transgenic mouse models, lineage tracing, and RNA sequencing. The experimental design and interpretation are appropriate, with some comments below. Writing is clear and excellent, and figures are high quality. Progress is made in addressing the main questions and a substantial quantity of additional data are yielded which are valuable for dental and skeletal researchers, making the study multifaceted and important. Some moderate concerns and minor suggestions are summarized below, which are important to address to clarify the approach and interpretation of the findings.

We appreciate the reviewer's very positive assessment of our study.

Moderate concerns

1) Authors do a nice job of comparing cementoblasts of acellular vs. cellular cementum throughout the course of this paper. Historically, cementoblasts have been traditionally compared to osteoblasts (as in the excellent review by Bosshardt, J Dent Res, 2005). It is a bit of a missed opportunity to not update this comparison in this study. Even if osteoblasts have not been reported among the cell populations here, can authors add context to their findings in the two (or more) cementoblast populations vs. osteoblasts in their previous work or in the reports of others?

We thank the reviewer for this excellent suggestion. We agree with the reviewer that the updated comparison between cementoblasts and osteoblasts will enhance the significance of our study.

To define the cementoblast-osteoblast relationship, we performed further analyses of our 10X VisiumHD dataset shown in the original **Figure 2**. Notably, our spatial dataset includes both cementoblasts and alveolar bone osteoblasts within the same tissue section. To achieve this goal, we created a band-like spatial bin on the alveolar bone surface to extract *Alpl*$^{+}$*Bglap*$^{+}$ alveolar bone osteoblasts (OB) (*Alpl*: logFC>0 AND *Bglap*: logFC>0) (**Figure R1-1**, included as **new Figure 2E**). Subsequently, we performed PCA and

pairwise differential expression analyses across AC-cementoblast, CC-cementoblast, and alveolar bone OB (**Figure R1-2**, included as **new Figure 2F and Supplemental Figure S3).**

- **Figure R1-1 (included as new Figure 2E,F)**

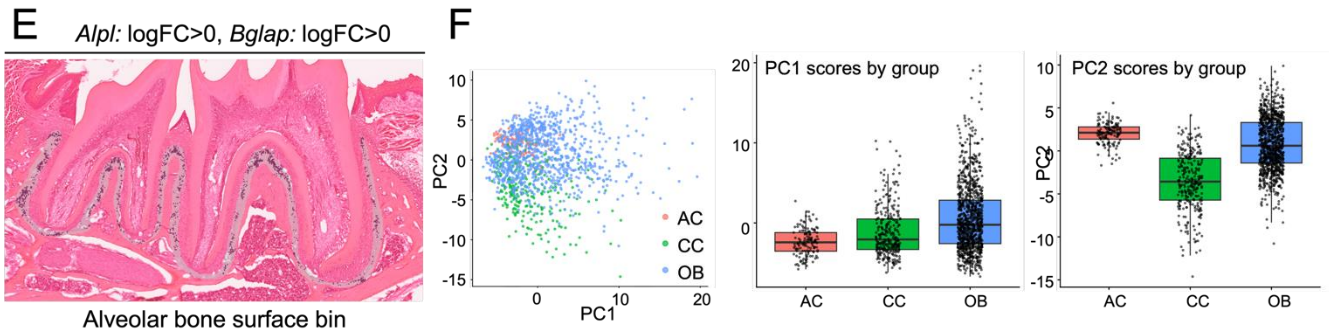

- **Figure R1-2 (included as new Supplemental Figure 3)**

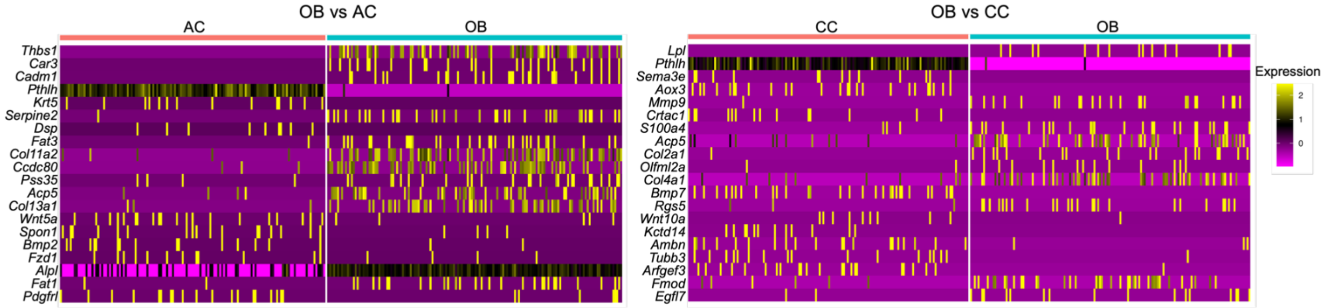

As shown in **Figure R1-1 (Figure 2E,F)**, acellular cementum (AC)-cementoblasts (red) and cellular cementum (CC)-cementoblasts (green) segregated clearly in the principal component analysis (PCA) plot, particularly on the second component (PC2), as expected from our results shown in **Figure 2B-D**. In contrast, alveolar bone osteoblasts (OB, blue) overlapped substantially with both AC-cementoblasts and CC-cementoblasts, supporting the notion that alveolar bone osteoblasts represent a heterogeneous cell population that shares fundamental matrix-producing programs with cementoblasts.

As shown in **Figure R1-2** (**Supplemental Figure 3**), pairwise differentially expressed gene (DEG) analysis revealed that AC-cementoblasts were enriched for tooth/cementum-associated features, including *Pthlh*, dental follicle-marker *Spon1*, dentin matrix genes such as *Dsp*, as well as Wnt-related components such as *Wnt5a* and *Fzd1*. In contrast, alveolar bone OBs were relatively enriched for canonical osteoblast markers (e.g., *Alpl*), as well as ECM/adhesion and remodeling-associated genes (e.g., *Thbs1*, *Serpine2*, *Ccdc80*, *Col13a1*), as well as resorption/remodeling markers (e.g., *Acp5*). Further, compared to CC-cementoblasts, alveolar bone OBs were enriched for perivascular/basement membrane-associated genes (e.g., *Rgs5*, *Col4a1*, *Olfml2a*), reflecting an active bone-surface microenvironment in the alveolar bone.

Overall, these additional analyses revealed that AC and CC-cementoblasts broadly share fundamental matrix-producing features with alveolar bone osteoblasts, while also possessing unique cementoblast-specific features, including expression of *Pthlh* and Wnt-related components. In addition, we have now cited the Bosshardt JDR review in the revised manuscript.

- **The following paragraph was added to the Results (Line 171-190):**

Historically, cementoblasts have often been compared to osteoblasts (Bosshardt et al., JDR 2005). To define the cementoblast-osteoblast relationship, we further extracted alveolar bone osteoblasts (OB) by creating a band-like spatial bin along the alveolar bone surface and selecting Alpl$^+$Bglap$^+$ cells (Alpl: logFC>0 AND Bglap: logFC>0) (Fig.2E). Principal component analysis (PCA) revealed that AC- and CC-

cementoblasts segregated clearly along PC2, indicating their distinct transcriptional identities (Fig.2F, red: AC, green: CC). In contrast, OB overlapped substantially with both AC- and CC-cementoblasts (Fig. 2F, blue: OB), consistent with the notion that alveolar bone osteoblasts represent a heterogeneous cell population that shares a core matrix-producing program with cementoblasts. Pairwise differential gene expression analysis revealed that AC-cementoblasts were enriched for tooth- and cementum-associated genes, including Pthlh, the dental follicle marker Spon1, dentin matrix genes such as Dsp, and Wnt-related components, including Wnt5a and Fzd1. In contrast, alveolar bone OBs were relatively enriched for canonical osteoblast markers (e.g., Alpl), extracellular matrix and adhesion-related genes (e.g., Thbs1, Serpine2, Ccdc80, Col13a1), as well as remodeling-associated markers (e.g., Acp5). Moreover, compared with CC-cementoblasts, alveolar bone OBs showed higher expression of perivascular and basement membrane-associated genes (e.g., Rgs5, Col4a1, Olfml2a), reflecting the dynamic bone-surface microenvironment in the alveolar bone. Together, these analyses indicate that AC- and CC-cementoblasts broadly share fundamental matrix-producing features with alveolar bone osteoblasts, while maintaining distinct cementoblast-specific features, including expression of Pthlh and Wnt-related components.

2) Why do the authors tend to keep using the term "peri-epithelial" cells in the Results and Discussion? Is it to make the point they are not epithelial in lineage and are not part of the wider follicle/PDL cell population? This is a point of minor confusion that could perhaps be clarified for readers to understand the importance of the distinction.

We intended to use "*peri-epithelial*" to describe a defined subset of dental follicle cells that resides immediately adjacent to Hertwig's epithelial root sheath (HERS). These cells are not epithelial in lineage. We have clarified the terminology for the first use in the Results section.

- **The following underlined sentence was added to the Result section (Line 295-296):**

Wif1 transcripts were detected exclusively in dental follicle (DF) and dental papilla (DP) mesenchymal cells surrounding Hertwig's epithelial root sheath (HERS) in the apical region at P3 and P6 (Fig.S4A). Here, we use the term "peri-epithelial" to denote the spatially defined subset of apical mesenchymal cells that reside immediately adjacent to HERS.

3) The stepwise discoveries that identify Wif1 as a cementoblast-selective factor are logical and clearly explained. However, Wif1 is reportedly expressed in other cells and tissues, potentially limiting its usefulness as a selective marker or molecular target for selectively affecting cementoblasts. Some publications suggest Wif1 in epithelium and the authors 'own results here suggest odontoblast/dentin are affected by using Wif1-Cre.

The reviewer is correct that *Wif1* is expressed in multiple cells and tissues at different developmental stages. It is, therefore, essential to validate *Wif1* expression in the developing tooth *in vivo*.

Our data, shown in **Figure 4A** and **Figure S7A**, clearly demonstrate that *Wif1* mRNA expression is highly restricted to apical mesenchymal cells surrounding HERS but is undetectable within HERS itself at the onset of cementogenesis at P6, supporting the utility of *Wif1* as a marker for cementoblast precursors. Further, we demonstrated that *Wif1-creER* does not mark any $CK5^+$ dental epithelial cells at any time point of P6, P15, P25, or P8W after the short-chase (**Figure 4C**), further demonstrating the mesenchyme-specificity of our *Wif1-creER* allele.

However, as shown in **Figure R1-3**, the reviewer is also correct that the descendants of *Wif1-creER*$^+$ cells (Wif1CE-tdT$^+$ cells) contribute to odontoblasts, in addition to cementoblasts, while epithelial tissues remained unlabeled.

Figure R1-3 (for reviewers only)

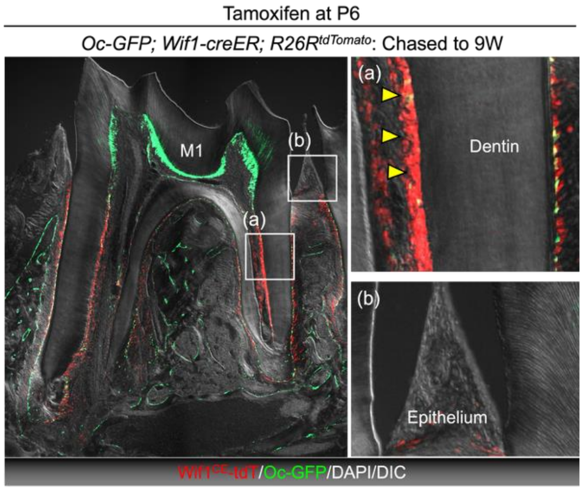

In the revised manuscript, we clarified that Wif1^{CE}-tdT^{+} cells also contributed to odontoblasts.

- **The following sentence was added to the Results (Line 326-328):**

Of note, Wif1^{CE}-P6 cells also contributed to odontoblasts of the root dentin (Fig.4D, left panel), indicating that Wif1^{+} cells also include odontoblast precursor cells.

4) Is it possible the cells analyzed by scRNA-seq around the apical root included late cementoblasts/early cementocytes? Dmp1 and Phex expression may suggest this possibility, though there are some different accounts for Dmp1- and Phex-expressivity in acellular vs. cellular cementum cells in the literature.

Pthrp-mCherry^{+} cells isolated at later time points (i.e., P15, P25, 8W) for scRNA-seq likely include late cementoblasts and early cementocytes, as indicated by *Dmp1* and *Phex* expression (**Figure 2C,D**).

Notably, our scRNA-seq data demonstrate that *Dmp1* and *Phex* are differentially expressed in acellular cementum (AC)- and cellular cementum (CC)-cementoblasts. *Dmp1* is expressed by both AC- and CC-associated cementoblasts (*Cementoblast α and β*); whereas *Phex* is expressed only by CC-associated cementoblasts (*Cementoblast β*). In the literature, *Dmp1* has been reported to be mainly expressed in cellular cementum and cementocytes; however, low or focal expression has also been reported in acellular cementum. *Phex* has been reported to be expressed by mature cementoblasts and or cementocytes; however, it has not been reported to be expressed in acellular cementum.

Therefore, our study defines for the first time the differential expression of *Dmp1* and *Phex* in AC- and CC-cementoblasts.

- **The following underlined sentence was added to the Result section (Line 284-285):**

AC-associated Cementoblast α cells exhibit enhanced Wnt signaling and cell-matrix organization signatures, whereas CC-associated Cementoblast β cells display mineralization-oriented gene expression <u>and likely include late cementoblasts and early cementocytes.</u>

5) The strategy here depends heavily on Pthlh/Pthrp as an accurate marker for acellular and cellular cementum cementoblasts and is derived from prior work from the group. Can the authors comment on the degree of specificity of Pthlh/Pthrp in cementoblasts and not in surrounding cells that can potentially contaminate cell analyses, e.g. PDL fibroblasts or pulp/odontoblast cells? Is there a possibility of missing some portion of cementoblasts that are weak or negative for Pthlh/Pthrp?

We appreciate the reviewer's important point regarding the specificity of *Pthlh*/PTHrP to cementoblasts. As shown in **Figure 1B**, while Pthrp-mCherry expression is highly specific to the tooth root surface, Pthrp-mCherry^{+} cells on the root surface include not only Oc-GFP^{+} cementoblasts (~52%), but also Oc-GFP^{negative} peri-cemental cells (~48%), as shown in **Figure 1C**. Therefore, while Oc-GFP^{+}Pthrp-mCherry^{+} "*double-positive*" cells are highly specific to acellular and cellular cementum cementoblasts, Oc-GFP^{neg}Pthrp-mCherry^{+} "*single-positive*" cells include at least in part those coinciding with PDL fibroblasts or other types of cells. This does not interfere with our goal, as we utilized single-cell RNA-seq (**Figure 3**) to sort out non-cementoblast Pthrp-mCherry^{+} cells for subsequent analyses.

Regarding the reviewer's second point regarding the possibility of PTHrP^{low} cementoblasts, it is unlikely, as essentially all Oc-GFP^{+} cementoblasts on the root surface are Pthrp-mCherry^{+} (~98%). We emphasize this efficiency in the revised manuscript.

- **The following underlined words were added to the Result section (Line 134-136):**

Collectively, these results demonstrate that the Pthrp-mCherry and Oc-GFP double-reporter system reliably <u>and efficiently</u> identifies cementoblasts associated with both AC and CC, as well as peri-cementoblast populations across distinct stages of tooth root formation.

6) According to authors, the phenotype of the Wif1-Cre:Ctnnb1 conditional KO mice indicates defects in dentinogenesis. In that case, extreme caution must be used because any dentin defect can cause secondary developmental defects in cementum formation that may not reflect direct effects on cementoblasts, but rather defects in the dentin scaffold onto which cementum is deposited.

To determine whether the observed defects in cementum formation are secondary to dentin defects, we examined the dentin architecture of the Wif1-βCat cKO molars. As discussed above, *Wif1-creER*-marked (Wif1^{CE}-tdT^{+}) cells at P3 contribute to odontoblasts of the root dentin. As a result, tdTomato reliably labels dentinal tubules, allowing us to assess dentinal tubule organization of the Wif1-βCat cKO molars.

- **Figure R1-4 (included as new Supplementary Figure 9A)**

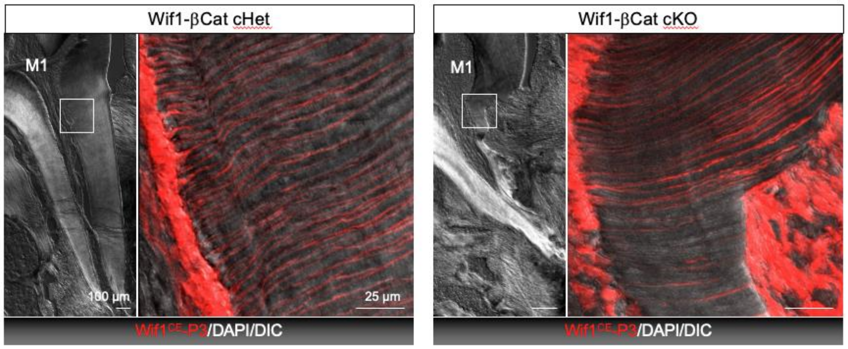

As shown in **Figure R1-4**, dentinal tubule organization appeared largely normal in cKO molars compared with cHet controls, despite a focal dentin irregularity at the cervical "notch" region. Therefore, defects in cementum formation are unlikely to be secondary to underlying dentin defects in Wif1-βCat cKO molars.

- **The following underlined sentence was added to the Result section (Line 360-363):**

Histological analyses revealed a prominent structural defect in Wif1-βCat cKO tooth roots, characterized by a "notch" below the cemento-enamel junction, corresponding to defective AC formation and focal irregularity of the underlying dentin (Fig. 5B, right panel, yellow arrowheads). Dentin and cementum deposition resumed apically beneath the notch, accompanied by robust CC formation near the tooth root apex (Fig. 5B, right panel, green dotted lines and arrowheads). Importantly, dentin architecture appeared largely preserved in Wif1-βCat cKO molars, as indicated by the presence of organized, parallel tdTomato⁺ dentinal tubules (Fig.S7A), suggesting that the cervical cementum defect is unlikely to arise secondarily from impaired dentin formation.

7) The appearance of Wif1-Cre:Ctnnb1 conditional KO mouse molars (especially first molars) in Figure 5B strongly resembles cervical root resorption, which can accompany acellular cementum defects. Because the age here is advanced, it is difficult to confirm if this is a developmental phenotype in dentin and cementum or aggressive resorption of roots. Developmental defects can look like thin dentin and cementum, lack of cementum by histology stains, and PDL detachment due to poor integration sharper's fibers; however, this root surface appears to be scalloped, reminiscent of resorption. This is important to clarify to guide the interpretation.

To determine if the cervical root abnormalities occurred due to aggressive cervical root resorption, we performed TRAP staining to assess the presence of active root-resorbing multinucleated osteoclasts. Importantly, as shown in **Figure R1-5**, we observed no TRAP-positive multinucleated cells along the cervical root surface corresponding to the AC region in both Wif1-βCat cHet and Wif1-βCat cKO molars. In contrast, robust TRAP-positive cells were detected in the alveolar bone region within the same sections, serving as an internal positive control for staining quality.

These findings argue against aggressive cervical root resorption, supporting our interpretation that the tooth root surface abnormalities of Wif1-βCat cKO molars reflect a developmental defect in cementum formation rather than active pathological resorption.

- **Figure R1-5 (included as Supplemental Figure 9B)**

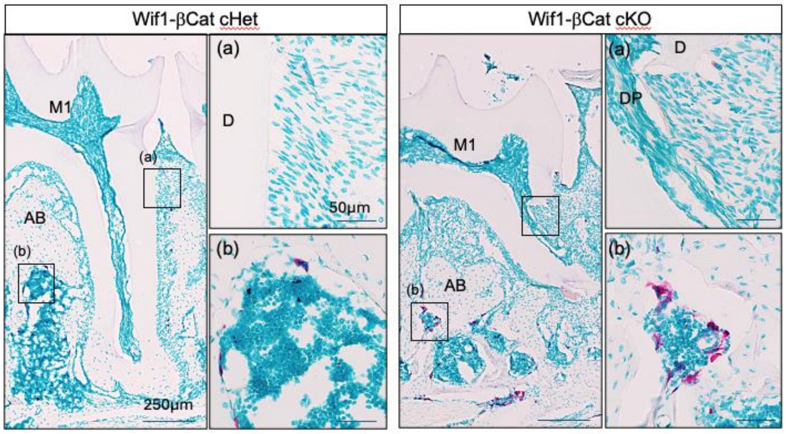

- **The following sentences were added to the Result section (Line 363-368):**

In addition, to determine whether the cervical root abnormalities reflected aggressive root resorption, we performed TRAP staining to assess osteoclast activities. No TRAP⁺ multinucleated cells were detected along the cervical root surface corresponding to the AC region in either genotype (Fig.S7B), indicating that the tooth root surface abnormalities observed in Wif1-βCat cKO molars represent a developmental defect in cementum formation rather than active pathological resorption.

Minor suggestions

1) The “coronal” portion of the root is described throughout the manuscript as the location of acellular cementum. I think this may cause confusion. I understand what the authors intend- this type of cementum is located in the coronal direction on the tooth, but it is not actually coronally located, which would typically mean covering the crown. Because there are forms of coronal cementum that actually do cover tooth crowns in some species (e.g. horses, cows, elephants, and other grass-eating species), I think this could be misinterpreted. Oftentimes, acellular cementum is described as located on the cervical (“neck”) portion of the tooth or tooth root and I think this is a more accurate description.

We sincerely thank the reviewer for this extremely insightful comment. We have replaced the term “*coronal portion*” with “*cervical portion*” throughout the manuscript (**Lines 27, 51, 101, 105, 109, 154, 427**).

2) Introduction – page 3 – line 55: The authors describe that acellular cementum lacks “intrinsic regenerative potential,” which I think is confusing or slightly inaccurate. Many papers describe repair cementum in the region of acellular cementum, thought to be produced by cementoblasts. While repair cementum is often described as more cellular in appearance and with unclear bonding to dentin and PDL, it does show repair capacity for the tissue.

We appreciate the reviewer’s insightful remark. We have replaced “*lacks*” with “*shows limited*” for accuracy.

- **The following underlined words were modified in the Result section (Line 55):**

However, in contrast to cellular cementum (CC), AC shows limited intrinsic regenerative capacity following periodontal destruction.

3) Figure 1: This SEM exploration of acellular vs. cellular cementum structure is a bit superficial and does not break new ground- these types of images and descriptions have appeared in many past papers. It is a nice way to ease into the more substantial portions of the paper and introduce non-dental readers to the anatomy and appearance, but does not add much overall to the advances of the study.

We appreciate the reviewer’s assessment. We understand that acellular and cellular cementum have been characterized previously using SEM. As the reviewer noted, **Figure 1** is included to recapitulate and highlight the anatomical locations and ultrastructural appearances of AC and CC. We believe this introductory figure provides an essential context for the following spatial and single-cell analyses for non-dental readers. We therefore opted to keep **Figure 1** in the main figure.

- **The following underlined sentence was added to the Result section (Line 100):**

We first examined the structural characteristics of the cementum using scanning electron microscopy (SEM) for anatomical reference.

4) Results – page 10 – line 258: HERS is mentioned in the context of 9 week old animals (unless I am misunderstanding), but HERS would no longer be present at this age that is well past the root development stage. There may be epithelial rests of Malassez, but not HERS proper. Please clarify.

We have now clarified the term in the revised manuscript by restricting “HERS” to the developmental stage (P17) and instead using “rests of Malassez (ERM)” at later stages (P25 and 9 weeks).

- **The following underlined sentence was modified in the Result section (Line 311-312):**

Additional short-chase analyses of Wif1creER/+; R26RtdTomato mice pulsed at P15, P25, and 8 weeks (examined at P17, P27, and 9 weeks, respectively) consistently revealed Wif1^{CE}-tdT^{+} cells surrounding CK5^{+}

epithelial cells in the apical region, specifically, HERS at P17, and epithelial rests of Malassez (ERM) at P27 and 9 weeks (Fig.4C-b2-4).

5) Figure 1A and C: The tooth schematic does not illustrate acellular vs. cellular cementum very clearly, or just cementum from dentin and PDL, for that matter.

We have revised the tooth schematic in **Figure 1A** to more clearly delineate the dentin, cementum, and PDL, and to highlight the distinct locations of AC and CC along the root surface.

- **Revised Figure 1A**

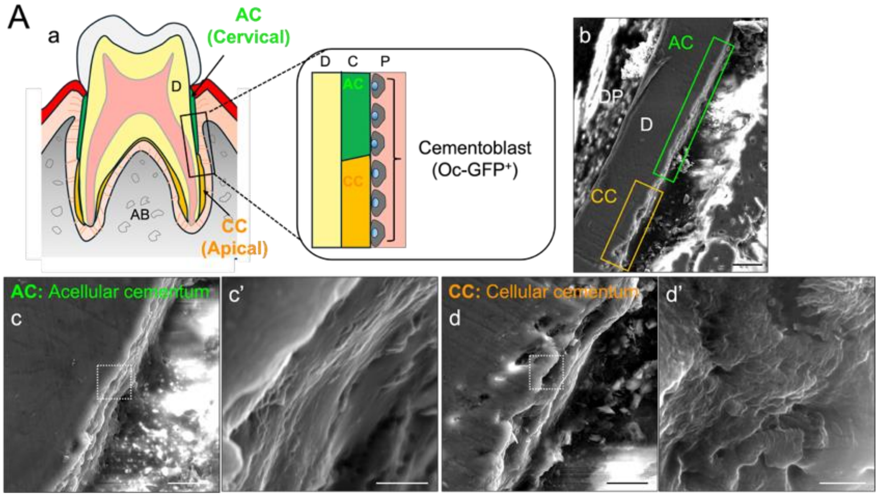

Reviewer #2:

This study provides fundamental insights into acellular cementum (AC) development, essential for periodontal attachment. Using integrated spatial/single-cell transcriptomics and Wif1-creER lineage tracing, the authors: (1) identify AC-formingc ementoblasts as a unique noncanonical mineralizing population distinct from cellular cementum (CC)-forming cementoblasts, characterized by specific cell-matrix and Wnt signaling signatures; (2) define their exclusive origin from Wif1+ peri-epithelial apical precursors via canonical Wnt activation; and (3) establish canonical Wnt as a molecular switch directing Wif1+ precursors toward the AC lineage, revealing how this pathway diversifies to generate distinct root mineralized tissues (AC vs. CC). Significantly, this work resolves the long-debated origin of AC cementoblasts, establishes a crucial developmental/molecular dichotomy between AC and CC formation, and proposes a novel targeted strategy (e.g., activating Wnt in progenitors) to regenerate AC and restore periodontal attachment lost to disease, addressing a key clinical challenge. This study is recommended for publication.

We appreciate the reviewer's very positive assessment of our study.

Reviewer #3:

In this study, Komori et al. employed spatial transcriptomics, single-cell RNAsequencing, lineage tracing, and genetic loss-of-function analyses to identify Wif1^{+}peri-epithelial apical mesenchymal cells as a primary source of acellular cementum (AC)-forming cementoblasts. The authors demonstrated that specification of this lineage is strictly dependent on canonical Wnt/β-catenin signaling during early root development, and described the molecular and developmental distinction between AC-forming and cellular cementum (CC)-forming cementoblasts. Based on these findings, they proposed that targeting this Wnt-dependent pathway could represent a potential strategy for regenerating AC to restore periodontal attachment.

Major points:

1. Mechanistic insights remain to be further explored. The authors proposed Wnt signaling as a binary switch but failed to delineate the downstream transcriptional network executing the AC program. The key target genes of β-catenin-mediated signaling in Wif1^{+} cells remain unidentified. Furthermore, the nature of the proposed "Wnt-independent" pathway for CC formation is unexplored, at least it should be discussed.

We appreciate the reviewer’s suggestion to further explore mechanistic insights into the AC-forming transcriptional program. To address this, we performed further in-depth analyses on our integrated scRNA-seq dataset presented in **Figure 3**.

According to RNA velocity analysis by VeloVAE, Wif1^{+} cells in Cluster 0 undergo bifurcated single-cell trajectories toward AC-cementoblast/Cementoblast α (Cluster 4) and CC-cementoblast/Cementoblast β (Cluster 9) (**Figure 3B**). To focus on the cementoblast lineage, we extracted the relevant clusters: Cluster 0 (Wif1+), Cluster 3 and 4 (AC lineage), and Cluster 9 (CC lineage) for subsequent analyses (**Figure R3-1A, new Supplemental Figure S5**).

Subsequently, we examined the branch point within the Wif1^{+} origin-state cluster (Cluster 0), which gives rise to both AC lineage (toward Cluster 4) and CC lineage (toward Cluster 9) (**Figure R3-1A**). Using CellRank-inferred fate probabilities toward AC versus CC lineages, we identified AC- and CC-biased cells within Cluster 0 (AC-primed and CC-primed; **Figure R3-1B**).

- **Figure R3-1 (included as Supplemental Figure S5)**

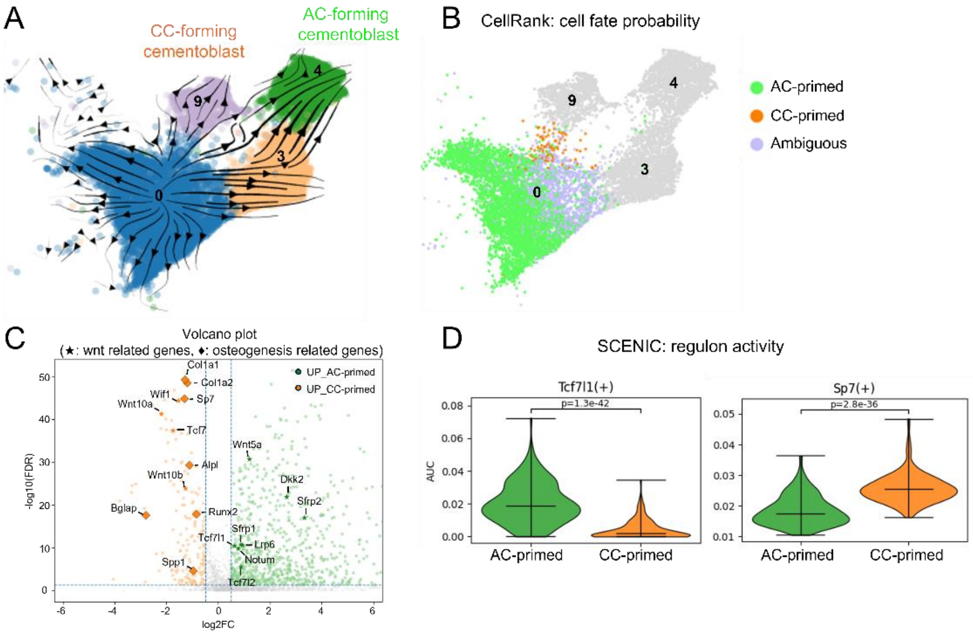

E

Overlap: Tcf7l1 putative targets (top30) ∩ branchpoint DE (top50, AC-up)

gene	regulon_rank	regulon_weight	de_rank	logfoldchanges	pvals_adj	scores
Smoc2	7	1.24	10	5.42	2.1e-41	13.82138729095459
Fbn1	16	1.02	2	4.54	5.8e-49	15.122696876525879
Ebf1	22	0.84	24	5.19	1.4e-35	12.757072448730469
Abcc9	25	0.76	46	2.68	2.3e-29	11.54052734375
Igf1	27	0.69	19	16.33	3.2e-37	13.059803009033203

We next performed **a branchpoint differential expression analysis** using a balanced approach by comparing equal numbers of cells from the upper and lower tails of the fate-bias score (Δ = p_AC - p_CC, CellRank-inferred fate probability; *n*=200 cells per group). This analysis revealed distinct programs associated with early commitment. AC-biased cells were enriched for canonical Wnt/TCF-LEF-associated components and feedback regulators (e.g., *Tcf7l1/2* and *Notum*; **Figure R3-1C**, green), whereas CC-biased cells preferentially expressed an osteogenic-like program including Sp7 (**Figure R3-1C**, orange)

To further delineate the downstream transcriptional network, we **integrated SCENIC regulon activity with the branchpoint analysis**. We detected significantly higher Tcf7l1(+) regulon activities in AC-primed cells, and higher Sp7(+) regulon activities in CC-primed cells (**Figure R3-1D**). This establishes Tcf7l1-centered and Sp7-centered transcriptional modules as candidate regulators of AC and CC commitment, respectively. Notably, some of the putative TCF7L1 targets overlapped with AC-upregulated branchpoint differentially expressed genes (DEGs), pointing to a concise set of candidate effectors (*Smoc2, Fbn1, Igf1, Abcc9, Ebf1*; **Figure R3-1E**). Of these identified factors, *Smoc2* has been recently implicated in WNT ligand trafficking in the mesenchymal stem cell niche of the rodent incisor (Chen Z et al., *Nat Commun.* 2025). Therefore, these analyses identify TCF7L1 as a key target of β-catenin-signaling in Wif1□ cells.

Finally, regarding the reviewer's point regarding the "Wnt-independent" pathway for CC formation, our data support the notion that CC commitment is comparatively less dependent on the canonical TCF7L1/2-associated module and instead is biased toward an Sp7-linked osteogenic transcriptional program.

- **The following paragraph was added to the Results (Line 253-273):**

We next sought to identify lineage-determining cues that direct Wif1$^+$ cells toward AC-cementoblast fates. To gain insight into the transcriptional networks underlying the AC-forming program, we performed a branchpoint-focused analysis of the Wif1$^+$ origin-state population (Cluster 0), which gives rise to both AC- and CC-cementoblasts (Fig.S5A). CellRank-based fate bias analysis revealed AC- and CC-primed subpopulations within Wif1$^+$ Cluster 0 (Fig.S5B). We then conducted branchpoint differential expression (DE) analysis comparing AC- and CC-primed Wif1$^+$ subpopulations. This analysis identified an AC-biased transcriptional module enriched for canonical Wnt/TCF-LEF-associated components and feedback regulators, including Tcf7l1, Tcf7l2, and Notum. In contrast, CC-biased cells preferentially engaged an Sp7-linked osteogenic transcriptional program (Fig.S5C). To further characterize these regulatory states, we integrated SCENIC regulon activity with the branchpoint analysis. This analysis revealed significantly Tcf7l1(+) regulon activity in AC-primed Wif1$^+$ cells, whereas Sp7(+) regulon activity was enriched in CC-primed Wif1$^+$ cells (Fig.S5D). Notably, several TCF7L1 targets overlapped with AC-upregulated branchpoint DEGs, highlighting a concise set of candidate downstream effectors, including Smoc2, Fbn1, Igf1, Abcc9, and Ebf1 (Fig.S5E). For example, Smoc2 has recently been implicated in WNT ligand trafficking in the mesenchymal stem cell niche of the incisor. In contrast, CC formation appears less dependent on the TCF7L1/2-associated module and is instead biased toward an Sp7-centered osteogenic program. Together, these findings refine our proposed "binary switch" model by identifying distinct downstream transcriptional modules that execute AC versus CC lineage commitment, and by highlighting TCF7L1 as a key transcriptional effector of β-catenin signaling in Wif1□ cells.

2. Functional validation needs to be further improved. The phenotypic analysis following β-catenin deletion is morphology-oriented and quite descriptive (eg. absence of AC, cell fate shift). Besides M1 molar, M2/M3 molars also developed abnormally and should be described. In addition, biomechanical assessment of the periodontium (e.g., tensile strength, tooth mobility) could be carried out in the mutant models to prove that AC absence directly compromises periodontal attachment function.

We appreciate the reviewer's remark. Following the reviewer's suggestion, we have now performed the following additional analyses.

First, we have expanded our analysis beyond M1 and described the phenotypes of the second molars (M2) in Wif1-βCat cKO mice. Similar to M1, AC coverage was completely absent on the M2 root surface, while the CC area was reduced in Wif1-βCat cKO compared with cHet controls (**Figure R3-2, new Supplemental Figure 7C**).

- **Figure R3-2 (included as new Supplemental Figure 9C)**

M2 root surface (3M, tamoxifen at P3)

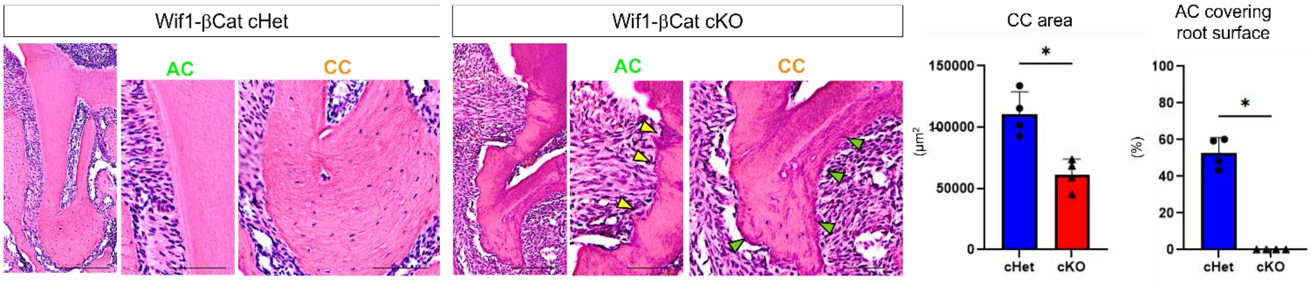

Second, to provide evidence that the absence of acellular cementum (AC) is associated with compromised periodontal attachment in Wif1-βCat cKO molars, we have performed Picrosirius Red (PSR) staining to visualize PDL collagen fiber insertion and added the statement. (**Figure R3-3, new Supplemental Figure 7D**).

- **Figure R3-3 (included as new Supplemental Figure 9D)**

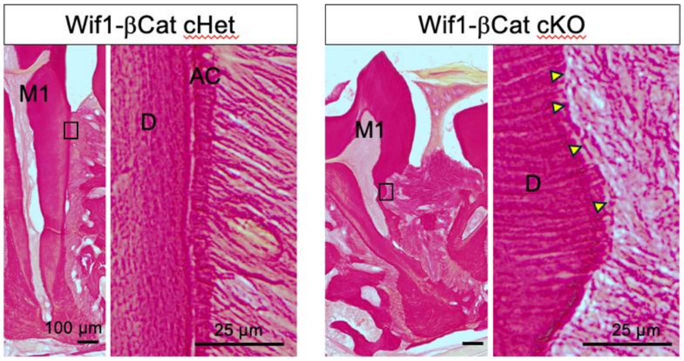

As shown in **Figure R3-3**, the root surface of Wif1-βCat cKO molars was completely absent with acellular cementum and periodontal ligament fiber insertion.

Additionally, we would like to respectfully point out that there is no established modality for biochemical assessment of the periodontium to evaluate periodontal attachment function. There is no established method to measure tensile strength or tooth mobility in vivo. One possible approach is to use atomic force microscopy (AFM) to measure mechanical properties using tissue sections; however, this requires unfixed, non-decalcified sections of murine molars, for which the protocol has not yet been established in the field. We feel that this is beyond the scope of our current study. We appreciate the reviewer's suggestion for future research.

- **The following underlined sentence was added to the Results (Line 352-353):**

In contrast, Wif1-βCat cKO molars exhibited significantly shorter M1 roots relative to Wif1-βCat cHet molars (Fig.5C), associated with abnormally developed M2 and M3 (Fig.5B), indicating that complete, but not partial, loss of β-catenin in Wif1^{+} cells impairs tooth root formation.

- **The following underlined sentence was added to the Results (Line 371-374):**

A similar phenotype was also observed in M2 (Fig.S7C). Consistent with the structural role of AC in periodontal attachment, Picrosirius Red staining revealed dense collagen bundles inserting into the AC in Wif1-βCat cHet molars, whereas fiber insertion at the root surface was markedly reduced in Wif1-βCat cKO molars lacking AC (Fig.S7D).

3. The work is exclusively based on mouse developmental models. The direct relevance to human AC biology, homeostasis, and the pathological/regenerative process during adult periodontitis is uncertain. It is suggested to supplemented experiments of human samples to enhance the clinical application value.

We thank the reviewer for raising this important point regarding the translational relevance of our mouse developmental findings to human acellular cementum (AC) biology, homeostasis, and periodontal regeneration. We agree that adding the human context significantly strengthens the significance of this work.

To address this, we leveraged a publicly available scRNA-seq dataset of human third molars and reanalyzed a representative human tooth/periodontium single-cell atlas (GSE161267). This atlas was designed to profile the major cell populations of the dental pulp and periodontium. Periodontal cells were obtained by scraping the apical two-thirds of tooth roots without extensive collagenase digestion. Therefore,

this atlas is expected to be enriched for PDL-associated populations, rather than selectively enriched for cementum-associated cementoblasts.

- **Figure R3-4 (included as Supplemental Figure 6)**

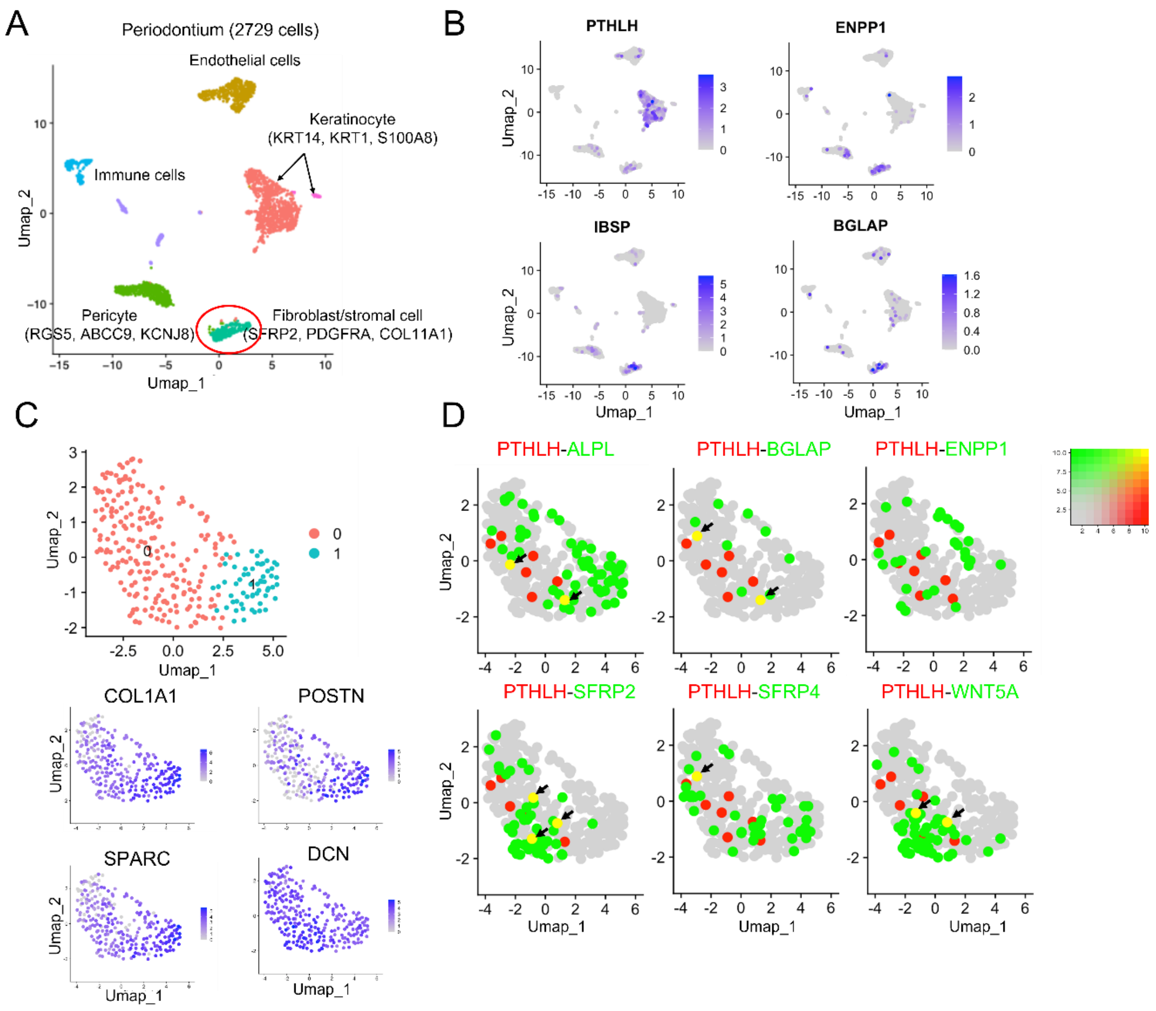

As shown in **Figure R3-4**, this human third molar scRNA-seq data included heterogeneous cell populations, including keratinocytes, endothelial cells, immune cells, pericytes, fibroblasts/stromal cells (**Figure R3-4A**). Our re-analysis did not resolve a discrete cementoblast cluster. However, we found that cells in the fibroblast/stromal cell cluster expressed *PTHLH*, along with cementoblast markers including *ENPP1*, *IBSP*, and *BGLAP* (**Figure R3-4B**). We focused on the fibroblast/stromal cell cluster for further analyses (**Figure R3-4C**). A small fraction of these cells co-expressed *PTHLH* with genes highlighted in our study, including *ALPL*, *BGLAP*, *SFRP2*, and *WNT5A* (**Figure R3-4D**). These findings confirm the existence of PTHrP$^+$ cementoblast-like populations in human molars.

- **The following paragraph was added to the Discussion (Line 274-280):**

To examine the human relevance of our findings, we reanalyzed a publicly available single-cell RNA-seq dataset of the periodontium from human third molars (Fig.S6A). Although a distinct cementoblast cluster was not clearly resolved, we identified a PTHLH$^+$ subset within the fibroblast/stromal cluster (Fig.S6B). Notably, a small fraction of these cells exhibited partial co-expression of ALPL, BGLAP, and Wnt-associated genes (SFRP2, SFRP4, WNT5A) (Fig.S6C,D). These observations support the presence of PTHrP$^+$ cementoblast-like populations in human molars.

Minor points:

1. The vertical axis of the column chart is inconsistent among different figures. A consistent and appropriate scale is recommended.

We have revised all bar graphs reported as percentages to use a consistent *y*-axis scale with a maximum of 100%. This change has been applied across the relevant panels (e.g., **Figure 5D, 5F, 5G**).

- **Revised Figure 5**

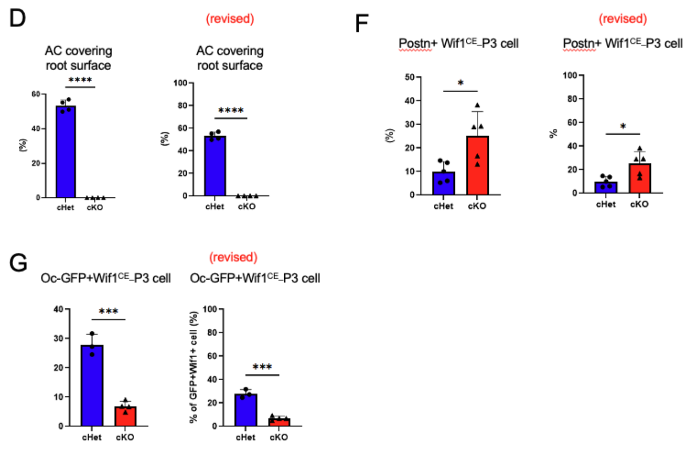

2. For single-cell RNA-seq analysis, specific marker genes used to annotate each cluster in feature plots or dot plots should be clearly stated.

Following the reviewer's suggestion, we have revised the main text to list the cluster identities and the key marker genes used for annotation. In addition, we provide a summary table listing cluster annotations and representative marker genes (**Supplementary Table S1**), which clarifies the basis for cluster labeling.

- **The following underlined words were added to the Results (Line 212-216):**

These included matrix-producing cementoblasts (Clusters 4, 9; Dmp1/Enpp1/Ibsp and Dmp1/Ifitm5/Phex), cementoblast precursors (Clusters 0, 1, 3; Ogn/Cxcl12/Sfrp1, Pcp4/Vcan/Bambi/ Crabp1, and Alpl/Thbs4/Col16a1), and periapical cells (Clusters 2, 7, 10; Nts/Hand2/Alx1, Itgbl1/Htra1/Col18a1, and Mgp/Nrp1/Prrx1), along with minor populations of epithelial and hematopoietic/immune cells. (Fig.3B, Fig.S3A, Table.S1).

- **New Supplemental Table S1**

Cluster	Annotation	Key markers used for annotation	Top markers
0	Cementoblast precursor	Ogn, Cxcl12, Sfrp1, Igf1, Igfbp4	Ogn, Igfbp4, Igf1, Fbln1, Gpx3, Mfap4
1	Cementoblast precursor	Pcp4, Vcan, Bambi, Crabp1, Id1	Pcp4, Hmgn3, Ptn, Vcan, Bambi, Id1
2	Periapical mesenchymal cells	Hand2, Nts, Alx1, Tnn, Csrp2	Csrp2, Nts, Hand2, Tnn, Lrrc17, Cald1
3	Cementoblast precursor	Alpl, Col16a1, Thbs4, Rora, Col1a1/Col1a2	Col1a2, Col3a1, Sparc, Col1a1, Col16a1, Rora
4	Cementoblast	Dmp1, Enpp1, Ibsp, Wnt5a, Ank	Ibsp, Ank, Timp3, Bmp3, Aqp1, Sparcl1
5	Mitochondrial transcript enriched	mt-Nd4, mt-Atp6, mt-Co3, mt-Co2, mt-Cytb	mt-Nd4, mt-Atp6, mt-Co3, mt-Nd2, mt-Co2, mt-Cytb
6	Epithelial cells	Krt5, Krt14, Trp63, Epcam, Krt17	Krt5, Krt14, Sfn, Dsp, Perp, Fabp5
7	Periapical mesenchymal cells	Itgbl1, Htra1, Col18a1, Col4a1, Serpine2	Htra1, Itgbl1, Wnt5a, Spon1, Col4a1, Col18a1
8	Cycling/proliferating cells	Mki67, Top2a, Stmn1, Hmgb2	H2afz, Hmgb2, Stmn1, Ran, Smc2, Hmgb1
9	Cementoblast	Dmp1, Ifitm5, Bglap, Phex	Col11a2, Col1a1, Smpd3, Ifitm5, Bglap, Sparc
10	Periapical mesenchymal cells	Mgp, Nrp1, Prrx1, Ebf1, Cygb	Mgp, Ebf1, Fbn1, Nrp1, Col4a1, Prrx1
11	Hematopoietic/immune cells	Ptprc, Lyz2, Csf1r, Tyrobp, Fcer1g	Fcer1g, Tmsb4x, Tyrobp, Laptm5, Ctss, Ftl1
12	Epithelial cels	Trp63, Pitx2, Wnt4, Dsc3	Wnt4, Dsc3, Trp63, Fxyd3, Runx1, Pitx2
13	Hematopoietic/immune cells	Ptprc, S100a8, S100a9, Ltf	S100a8, Lcp1, S100a9, Tyrobp, Cd52, Tmsb4x
14	Epithelial cells	Krt5, Krt14, Odam, Krt17	Krt14, Odam, Krt5, Perp, Spint2, Sfn
15	Hematopoietic/immune cells	Hba-a1, Hbb-bs, Alas2	Hba-a1, Hbb-bs, Hba-a2, Hbb-bt, Alas2, Snca
16	Hematopoietic/immune cells	Ptprc, Cd79a, Cd79b, Ebf1, Vpreb3	Cd79b, Cd79a, Ebf1, Vpreb3, Ptprcap, Chchd10
17	Endothelial cells	Pecam1, Cdh5, Kdr, Flt1, Vwf, Egfl7	Egfl7, Pecam1, Cdh5, Ecscr, Col4a1, Ramp2
18	Schwann/glial cells	Plp1, Mpz, Pmp22, S100b	Plp1, Dbi, Itih5, Gpm6b, Cryab, Lgi4

3. For scRNA-seq analysis in Figure 3, three key improvements are needed: i) Expression scale bars are missing; ii) Specific cell clusters/subtypes should be clearly defined in Figure 3B; iii) In Figure 3C, Wif1 expression used a different color scheme without any scale bar, better change to the same color scheme as other genes within this panel.

We have revised Figure 3 in accordance with the reviewer's suggestion. First, we annotated **Figure 3B** with the corresponding cluster categories to clarify the identities of clusters in the UMAP plot. Second, we added expression scale bars to the feature plots in **Figure 3C,D**. Third, since our "Wif1^{high}" visualization is a threshold-based classification rather than continuous expression intensity, we maintained it as a discrete high/low display and added an explicit legend to this panel.

- **Revised Figure 3B-D**

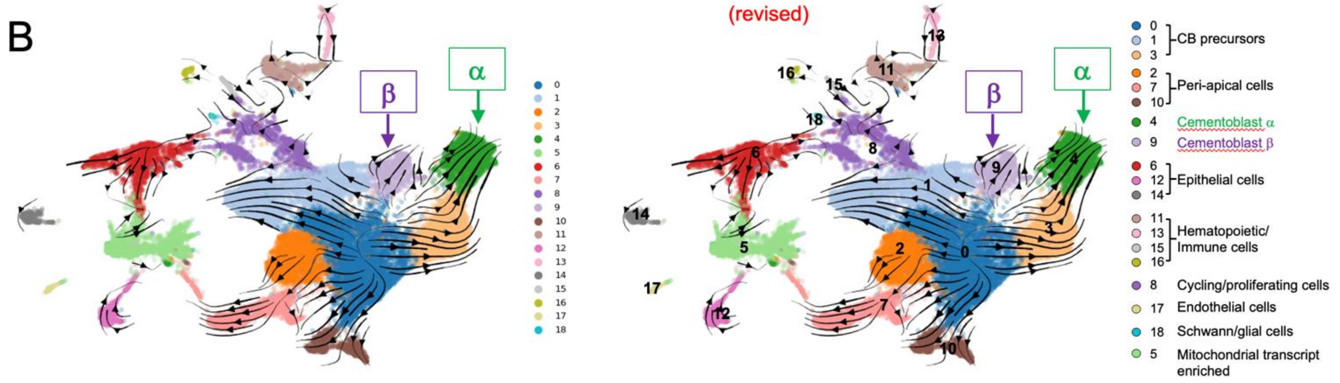

C

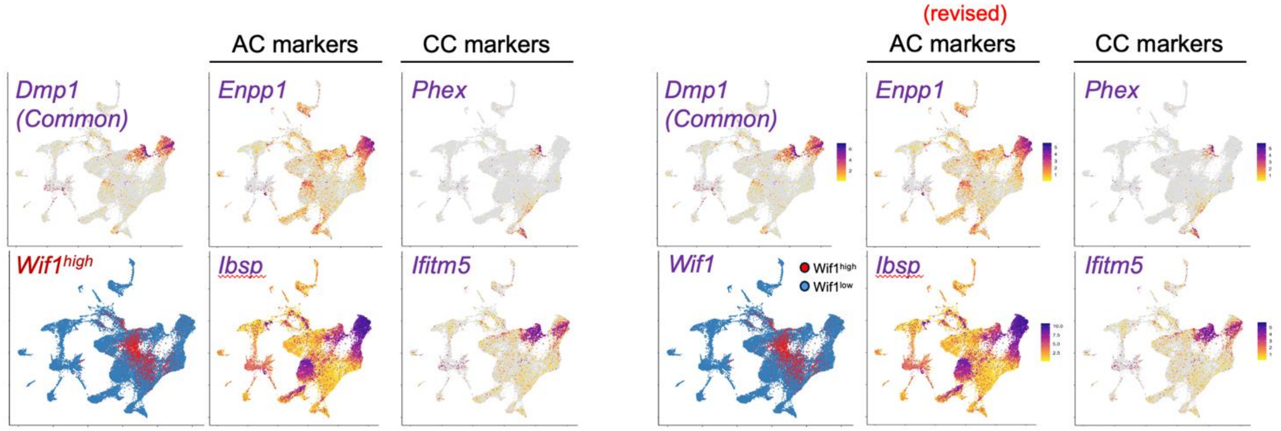

D

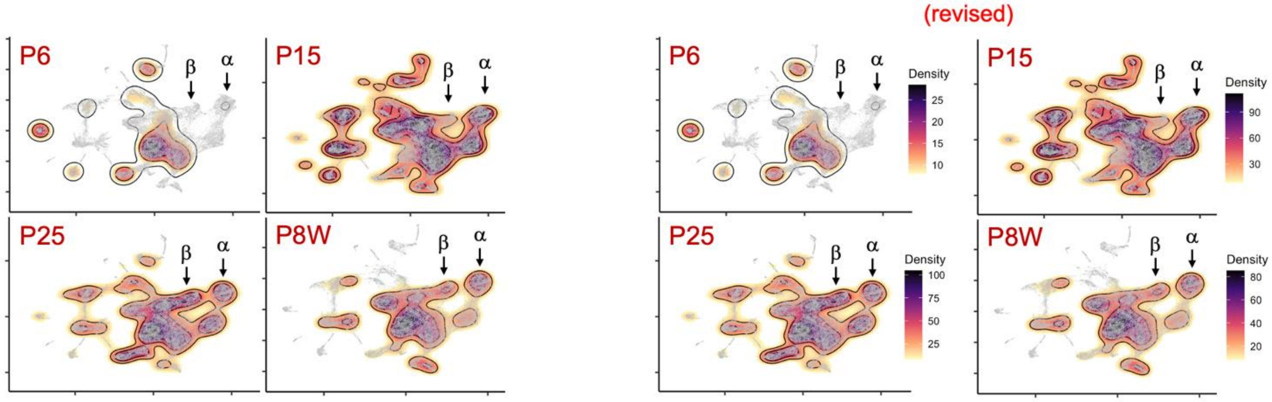

4. The AC was an avascular, cell-free matrix tissue. However, nuclear signals are perceptible within the mineralized structure in some immunofluorescence images (e.g.,Fig. S3E). These signals should be further verified.

Acellular cementum (AC) is often described as an “avascular, cell-free” tissue; however, “acellular” specifically refers to the absence of cementocytes embedded within the cementum matrix (i.e., lack of cementocyte lacunae), rather than the absence of cells on the cementum surface. Since cementoblasts are expected to exist on the root surface, nuclei can be visible adjacent to AC, particularly at the cementum-PDL interface.

In **Figure S3E**, the nuclear signals noted by the reviewer correspond to cells on or near the root surface, such as cementoblasts and adjacent periodontal cells. These are not nuclei embedded within the AC matrix. To avoid confusion, we have revised the figure and the figure legend. We have added annotations to more clearly delineate the AC matrix from the adjacent cellular layer and clarify the surface of the cementum and cementocyte lacunae.

- **Revised Figure 1**

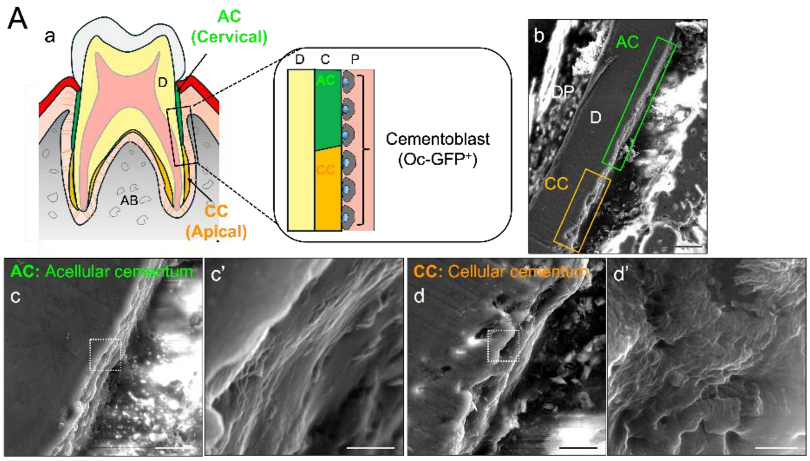

- **Revised Supplemental Figure 3E**

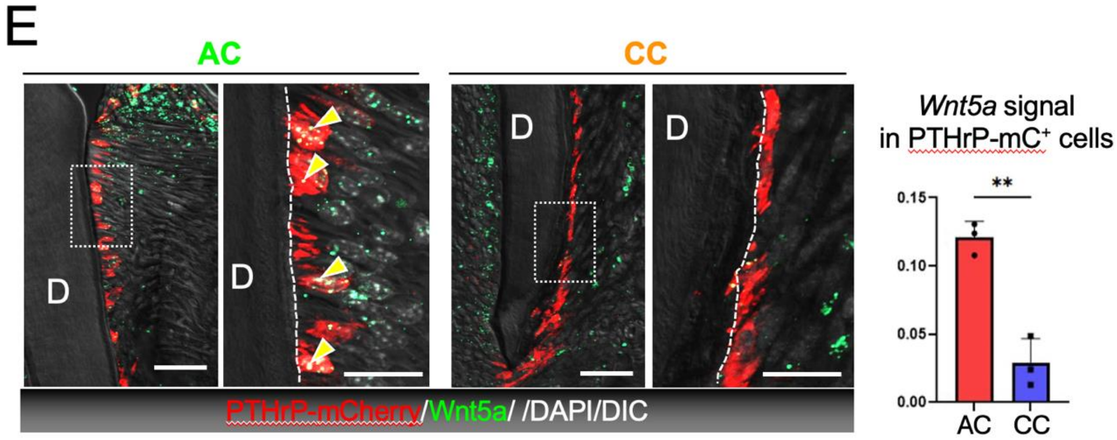

Supplement: Supplementary file 2 — Peer Review file [file 41467_2026_72712_MOESM2_ESM.pdf]
